# *In Silico* Characterization of *bla*_NDM_-Harboring Conjugative Plasmids in Acinetobacter Species

**DOI:** 10.1128/spectrum.02102-22

**Published:** 2022-10-27

**Authors:** Biao Tang, Chenyu Wang, Dongchang Sun, Hui Lin, Jiangang Ma, Hengzhao Guo, Hua Yang, Xiaobin Li

**Affiliations:** a State Key Laboratory for Managing Biotic and Chemical Threats to the Quality and Safety of Agro-products & Institute of Agro-product Safety and Nutrition, Zhejiang Academy of Agricultural Sciences, Hangzhou, Zhejiang, China; b College of Biotechnology and Bioengineering, Zhejiang University of Technology, Hangzhou, China; c Department of Radiation Oncology, Zhuhai People’s Hospital (Zhuhai Hospital affiliated with Jinan University), Zhuhai, Guangdong, China; d Zhuhai Precision Medical Center, Zhuhai People's Hospital (Zhuhai Hospital affiliated with Jinan University), Zhuhai, Guangdong, China; University of Pittsburgh School of Medicine

**Keywords:** *Acinetobacter*, New Delhi metallo-β-lactamase, plasmid, conjugative

## Abstract

New Delhi metallo-β-lactamase (NDM)-producing clinical strains in Acinetobacter spp. have been recently reported in many countries and have received considerable attention. The vast majority of *bla*_NDM_ cases occur on conjugative plasmids, which play a vital role in disseminating *bla*_NDM_. To characterize the conjugative plasmids bearing *bla*_NDM_ genes in Acinetobacter spp., we analyzed the variants of *bla*_NDM_, conjugative transfer regions, genetic contexts of *bla*_NDM_, and the phylogenetic pattern of the 62 predicted *bla*_NDM_-positive plasmids, which were selected from 1,191 plasmids of Acinetobacter species from GenBank. We identified 30 conjugative plasmids from the 62 *bla*_NDM_-harboring plasmids in Acinetobacter species, with the *oriT* sites similar to plasmid pNDM-YR7 in our study, genes coding for relaxases of the MOB_Q_ family, genes encoding type IV coupling proteins (T4CPs) of the TrwB/TraD subfamily, and VirB-like type IV secretion system (T4SS) gene clusters. The genome sizes of all 30 pNDM-YR7-like plasmids ranged from 39.36 kb to 49.65 kb, with a median size of 44.56 kb. The most common species of Acinetobacter containing the *bla*_NDM_-positive conjugative plasmids was A. baumannii, followed by Acinetobacter lwoffii and Acinetobacter indicus. Notably, pNDM-YR7 is the first report on a *bla*_NDM_-positive conjugative plasmid in Acinetobacter junii. Moreover, all 30 *bla*_NDM_-positive conjugative plasmids in Acinetobacter species were found to contain genetic contexts with the structure IS*Aba14*-*aph(3′)-VI*-IS*Aba125*-*bla*_NDM_-*ble*. Our findings provide important insights into the phylogeny and evolution of *bla*_NDM_-positive plasmids of Acinetobacter species and further address their role in acquiring and spreading *bla*_NDM_ genes in Acinetobacter species.

**IMPORTANCE** Conjugative plasmids harboring the *bla*_NDM_ gene play a vital role in disseminating carbapenem resistance. In this study, we first report a conjugative plasmid, pNDM-YR7, in Acinetobacter junii. Based on the genomic characteristics of the *bla*_NDM_-positive pNDM-YR7, we performed *in silico* typing and comparative analysis of *bla*_NDM_-positive plasmids using the 1,191 plasmids of Acinetobacter species available in the NCBI RefSeq database. We analyzed the characteristics of *bla*_NDM_-positive plasmids, including the variants of *bla*_NDM_, genetic features associated with *bla*_NDM_, conjugative transfer regions, and the phylogenetic pattern of the *bla*_NDM_-positive plasmids. All 30 *bla*_NDM_-positive conjugative plasmids were found to contain an IS*Aba14*-*aph(3′)-VI*-IS*Aba125*-*bla*_NDM_-*ble* region. This study provides novel insights into the phylogeny and evolution of *bla*_NDM_-harboring conjugative plasmids and contributes to the repertoire of knowledge surrounding *bla*_NDM_-positive plasmids in the genus Acinetobacter.

## INTRODUCTION

New Delhi metallo-β-lactamase (NDM), belonging to the class B β-lactamases, can hydrolyze almost all β-lactam antibiotics (including carbapenems) with the exception of monobactams (1). NDM-1 was first reported in a Klebsiella pneumoniae strain, 05-506, isolated from a Swedish patient who was admitted to the hospital in New Delhi, India, in 2008 (2). Since then, NDM-1 and its variants have been reported in various species of *Enterobacteriaceae*, Pseudomonas, and Acinetobacter (3). To date, at least 28 variants of NDM have been reported (3, 4). NDM-1 and its variants are continuously spreading worldwide (3, 5), and NDM-producing pathogens in both humans and the environment have created a major therapeutic challenge for clinicians and have received considerable attention (6, 7).

In 2010, *bla*_NDM-1_-positive Acinetobacter baumannii was found in India, which was the first report of NDM-producing clinical strains in Acinetobacter species in the world (8). Since then, the emergence of NDM-producing Acinetobacter species has been reported in many countries of the world (9–11). In China, four A. baumannii isolates with *bla*_NDM-1_ were identified in four different provinces in 2010, which was the first report of *bla*_NDM-1_-positive clinical isolates in China (12). Since then, *bla*_NDM-1_ has been reported many times in several strains of Acinetobacter species (such as Acinetobacter lwoffii, Acinetobacter junii, and Acinetobacter pittii) from clinical, environmental, and farm animal samples in China (12–15).

Plasmids, especially conjugative plasmids, remain important microbial components that mediate horizontal gene transfer (HGT) and play a vital role in the dissemination of antimicrobial resistance genes (ARGs) (16–19). The replication region of the plasmid is required for the survival of a plasmid, and it consists of an origin of replication, genes encoding a replication initiator (Rep), and regulatory factors (20, 21). Most plasmids in A. baumannii encode Rep proteins belonging to the Rep_3 superfamily and replicase_PriCT family (21). The conjugative plasmids typically contain conjugative transfer regions in their genomes, consisting of the origin of transfer (*oriT*) region, relaxase gene, type IV coupling protein (T4CP) gene, and gene cluster for the bacterial type IV secretion system (T4SS) apparatus (22). Bacterial conjugation is initiated by the recognition and cleavage of the *oriT* site by the relaxase with the help of auxiliary DNA-binding proteins, forming a nucleoprotein complex called the relaxosome consisting of single-stranded DNA (ssDNA), relaxase, and auxiliary DNA-binding proteins (17). Then, the relaxosome is recruited by T4CP and subsequently transferred from the donor strain into the recipient strain via T4SS (23). NDM has been identified in various species of *Enterobacteriaceae* due to localization of *bla*_NDM_ on conjugative plasmids, enabling transfer and rapid dissemination of multidrug resistance (3, 24). However, there has been no systematic analysis of *bla*_NDM_-harboring conjugative plasmids in Acinetobacter species. With the increase in the amount of whole-genome/plasmid sequencing data, there is a need for large-scale analysis of *bla*_NDM_-positive plasmids in Acinetobacter species.

In this study, based on the genomic characteristics of the *bla*_NDM-1_-positive plasmid pNDM-YR7 from A. junii strain YR7, we performed *in silico* typing and comparative analysis of *bla*_NDM_-positive plasmids of Acinetobacter species using the plasmids of Acinetobacter species available in the NCBI RefSeq database. We analyzed the characteristics of *bla*_NDM_-positive plasmids of Acinetobacter species, including the variants of *bla*_NDM_ genes, genetic features associated with *bla*_NDM_, conjugative transfer regions, and the phylogenetic pattern of the *bla*_NDM_-positive plasmids. This study provides important insights into the phylogeny and evolution of *bla*_NDM_-positive plasmids of Acinetobacter species and further addresses their role in the acquisition and spread of *bla*_NDM_ genes in Acinetobacter species.

## RESULTS

### Description of phenotypic antimicrobial resistance.

**FIG 1:**
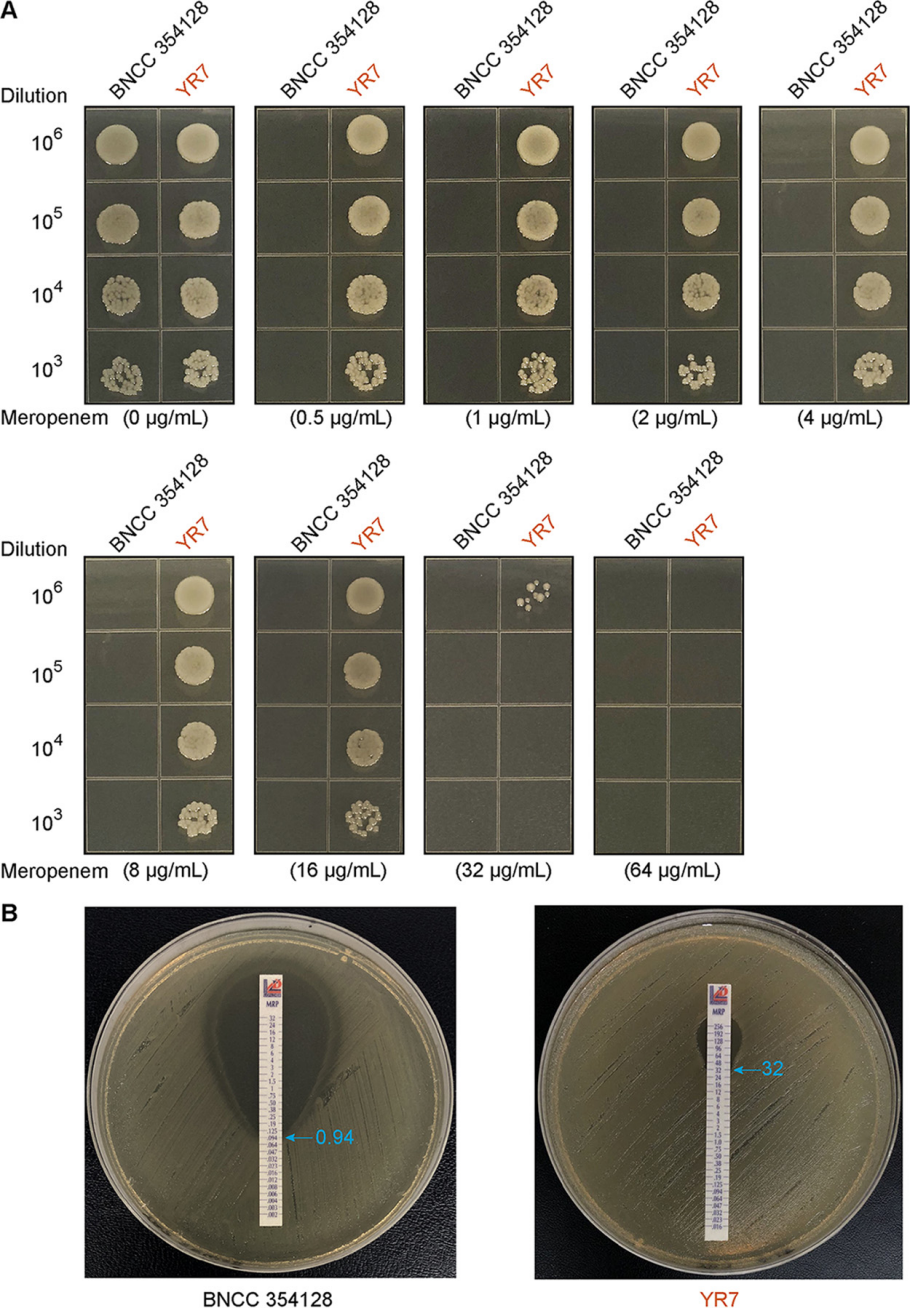
The insusceptibility of strain YR7 to meropenem. (A) Bacterial viability of strain YR7 on MHA plates containing various levels of meropenem. To determine the bacterial viability of strain YR7, the mid-log-phase cultures in serial dilution were spotted on MHA plates supplemented with meropenem at various levels (0.5, 1, 2, 4, 8, 16, 32, and 64 μg/mL) and maintained for 18 h at 37°C. The density of strain YR7 suspensions was adjusted to a 0.5 McFarland standard (1 × 10^8^ CFU/mL). Eight microliters of the diluted suspension with approximately 1 × 10^6^, 1 × 10^5^, 1 × 10^4^, 1 × 10^3^, and 1 × 10^2^ CFU/mL was inoculated on prepared MHA plates. The type strain BNCC 354128 of A. junii was used as the negative control. (B) Application of the Etest to compare the MIC values of meropenem in the A. junii strains BNCC 354128 and YR7.

A. junii YR7 (see Fig. S1 in the supplemental material) was positive for the *bla*_NDM_ gene, which was confirmed by carbapenem inactivation method (CIM) testing (Fig. 1). To better understand its antibiotic resistance phenotype, the susceptibility of strain YR7 to 14 antimicrobial agents was assessed. Antimicrobial susceptibility testing results showed that A. junii YR7 exhibited high MICs of ampicillin (256 μg/mL), amoxicillin-clavulanic acid (512/256 μg/mL), ceftiofur (64 μg/mL), ceftazidime (>256 μg/mL), florfenicol (128 μg/mL), tetracycline (32 μg/mL), spectinomycin (64 μg/mL), and meropenem (64 μg/mL) and was intermediate to ofloxacin (4 μg/mL) but remained susceptible to colistin (1.0 μg/mL), sulfisoxazole (0.5 μg/mL), trimethoprim-sulfamethoxazole (0.125/2.4 μg/mL), gentamicin (0.25 μg/mL), and enrofloxacin (0.06 μg/mL). Unlike strain BNCC 354128 (negative control), which grew on Mueller-Hinton agar (MHA) plates with no more than 0.5 μg/mL meropenem (Fig. 1A), strain YR7 consistently exhibited appreciable growth on MHA plates with up to 32 μg/mL meropenem (Fig. 1A). This is generally consistent with the fact that strain YR7 showed a high MIC of meropenem (32 μg/mL) in the Etest assays (Fig. 1B). Notably, the corresponding gene was confirmed to be *bla*_NDM_ positive by PCR and Sanger sequencing. Southern blotting was also performed to confirm the presence of *bla*_NDM_ on the plasmid (Fig. S2A).

### Genomic analysis of A. junii strain YR7.

The genome of A. junii YR7 comprises a chromosome with 3,392,646 bp (GenBank accession number CP059558) and one plasmid named pNDM-YR7 with 45,911 bp (GenBank accession number CP059559). Plasmid size was confirmed using an S1-nuclease pulsed-field gel electrophoresis (S1-PFGE) method (Fig. S2B). The GC content of the plasmid pNDM-YR7 was 38.82%, which is consistent with that in the chromosome (38.60%). The ResFinder results indicated that A. junii YR7 carried four acquired ARGs encoding resistance to aminoglycosides [*aph(3′)-VI*], tetracycline [*tet*(39)], florfenicol (*floR*), and carbapenems (*bla*_NDM-1_), which were all located on the plasmid, while no ARG was found to be located on the chromosome.

### General characteristics of *bla*_NDM_-positive plasmids in Acinetobacter species.

**FIG 2:**
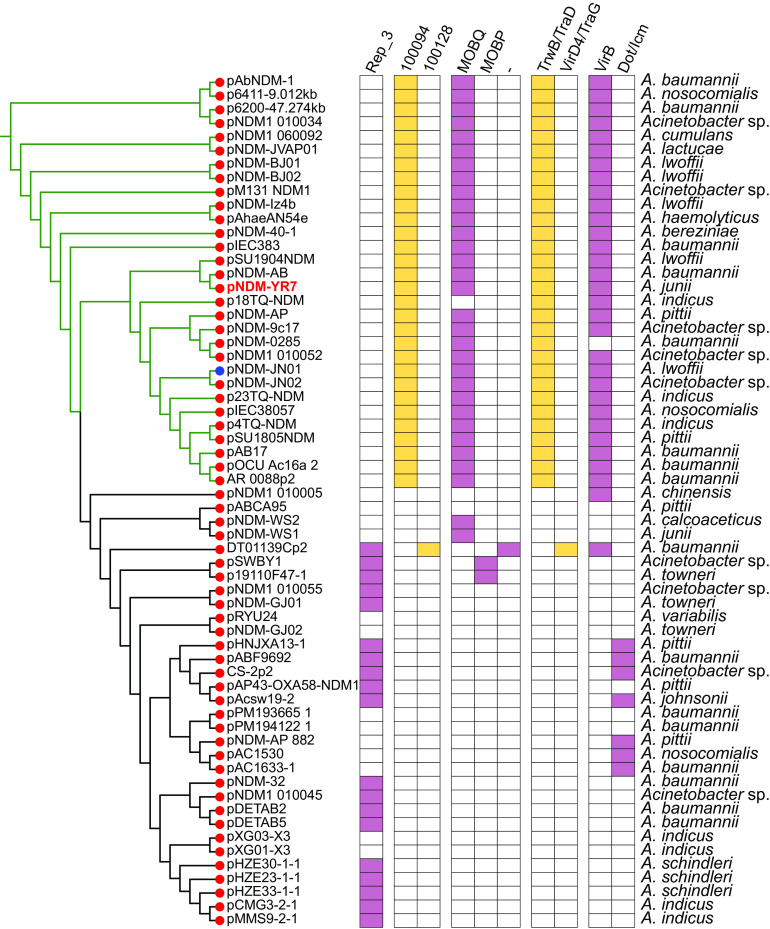
Characteristics of the 62 *bla*_NDM_-positive plasmids from 1,191 plasmids of Acinetobacter species from the GenBank RefSeq database. The five categories of information presented in this figure include the phylogenetic tree of 62 *bla*_NDM_-positive plasmids of Acinetobacter species, Rep genes, variants of *bla*_NDM_ genes, conjugative transfer modules (including *oriT*, relaxase, T4CP, and T4SS), and the taxonomy of species of host strains. Details of variants of *bla*_NDM_ genes are marked by the circles with different colors at the end of the branch of the phylogenetic tree with red for *bla*_NDM-1_ and blue for *bla*_NDM-14_. 100094 and 100128 were accession numbers of oriTDB (https://bioinfo-mml.sjtu.edu.cn/oriTDB/).

To characterize the *bla*_NDM_-positive plasmids distributed in Acinetobacter species, we selected 1,191 plasmids of Acinetobacter species from the NCBI RefSeq database for comparative analysis. A total of 237 plasmids bearing β-lactamase genes were identified from the 1,191 Acinetobacter plasmids using ResFinder local version software. Among the 237 plasmids containing β-lactamase genes, 62 were further identified as *bla*_NDM_-positive plasmids containing 62 *bla*_NDM_ genes (Table S2), including 53 *bla*_NDM_-positive plasmids distributed in 16 species of the Acinetobacter genus and nine *bla*_NDM_-positive plasmids belonging to Acinetobacter species (Fig. 2 and Fig. S3). The most common species bearing the *bla*_NDM_-positive plasmids was Acinetobacter baumannii (16 *bla*_NDM_-positive plasmids), followed by Acinetobacter indicus (7 *bla*_NDM_-positive plasmids), Acinetobacter pittii (6 *bla*_NDM_-positive plasmids), and Acinetobacter lwoffii (5 *bla*_NDM_-positive plasmids) (Fig. 2 and Fig. S3). For A. junii, two *bla*_NDM_-positive plasmids were found, including the plasmid pNDM-YR7 from A. junii YR7 (Fig. 2 and Fig. S3).

### Variants of *bla*_NDM_ genes in the *bla*_NDM_-positive plasmids in Acinetobacter species.

Among the 62 *bla*_NDM_-positive plasmids in Acinetobacter species, 62 *bla*_NDM_ genes belonged to two kinds of variants of *bla*_NDM_, including *bla*_NDM-1_ and *bla*_NDM-14_ (Fig. 2). Between the two variants of *bla*_NDM_, *bla*_NDM-1_ was found to be the more dominant (61 *bla*_NDM-1_ genes in 61 plasmids) (Fig. 2 and Table S2). In addition, the plasmid pNDM-JN01 (NZ_KM210086) from A. lwoffii strain JN49-1 was found to carry the *bla*_NDM-14_ variant.

### Genetic diversity of the *bla*_NDM_-positive plasmids in Acinetobacter species.

To obtain a comprehensive overview of *bla*_NDM_-positive plasmids, we constructed phylogenetic trees of all 62 *bla*_NDM_-positive plasmids in Acinetobacter species (Fig. 2). Based on the phylogenetic patterns, replication initiation protein (Rep) genes, and conjugative transfer modules of the plasmids, the 62 *bla*_NDM_-positive plasmids were classified into two main clades.

**FIG 3:**
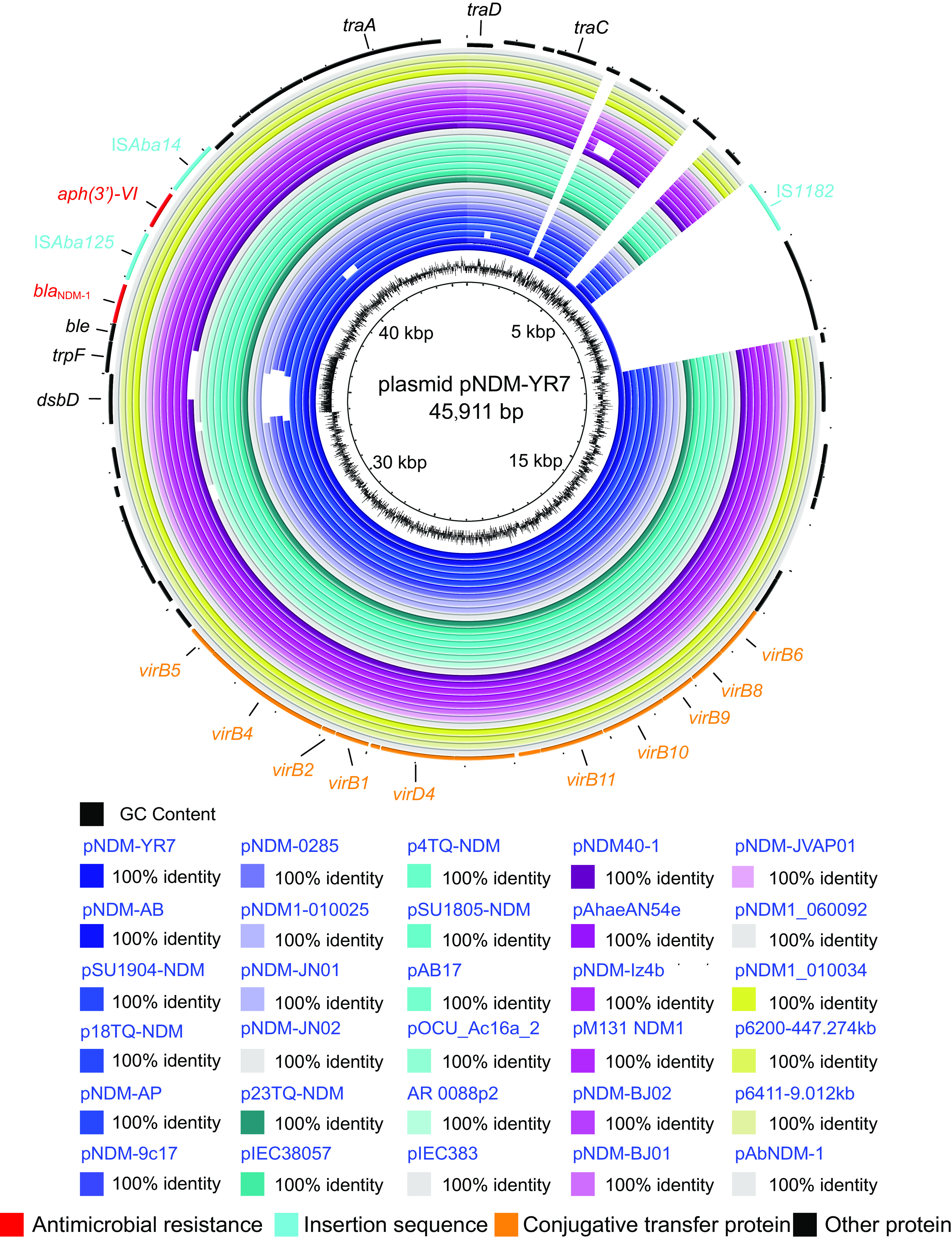
Schematic map of VirB-like T4SS gene clusters and *bla*_NDM_-associated genetic structures identified among the 30 *bla*_NDM_-positive conjugative plasmids in Acinetobacter species.

One clade containing 30 *bla*_NDM_-positive plasmids, including the plasmid pNDM-YR7, was found to represent the most common conjugative plasmid pattern carrying the *bla*_NDM_ gene in Acinetobacter species. The clade (termed the pNDM-YR7-like clade) included 29 *bla*_NDM-1_-positive plasmids and one *bla*_NDM-14_-positive plasmid, pNDM-JN01, accounting for approximately 48.4% of all *bla*_NDM_-positive plasmids in Acinetobacter species. All the plasmids belonging to the pNDM-YR7-like clade were found to share conjugative transfer regions similar to those of plasmid pNDM-YR7 in our study (Fig. 2). They were found to carry *oriT* sites similar to that of plasmid pNDM-YR7 (Fig. 2) with the 38-nucleotide (nt) core region sequence AGGGATTCATAAGGGAATTATTCCCTTATGTGGGGCTT. Almost all the *bla*_NDM_-positive conjugative plasmids belonging to the pNDM-YR7-like clade were found to carry genes coding for relaxases of the MOB_Q_ family, except that one plasmid was lacking in the relaxase gene (Fig. 2). All the pNDM-YR7-like plasmids carried the genes encoding T4CPs of the TrwB/TraD subfamily (Fig. 2). Almost all the *bla*_NDM_-positive conjugative plasmids belonging to the pNDM-YR7-like clade were found to contain *virB*-like T4SS gene clusters, except for one plasmid lacking T4SS genes (Fig. 2). Most of the *virB*-like T4SS gene clusters in pNDM-YR7-like plasmids were mainly composed of nine core genes (Fig. 3). Notably, the 30 pNDM-YR7-like plasmids do not have identifiable Rep genes.

For the other large clade, containing 32 *bla*_NDM_-harboring plasmids, 19 were found to carry the Rep proteins belong to the Rep_3 superfamily (Pfam: 01051). Only one plasmid (A. baumannii strain DT01139C plasmid unnamed2) was identified as the putative plasmid, including the 378-bp *oriT*-like region different from those of pNDM-YR7-like plasmids, an untypeable relaxase gene, T4CP of the VirD4/TraG subfamily, and the *virB*-like T4SS gene clusters. Notably, seven *bla*_NDM-1_-positive plasmids were found to carry the Dot/lcm-like T4SS gene clusters.

### General characteristics of the 30 pNDM-YR7-like plasmids.

We analyzed and compared the genome sizes and GC contents of all 62 *bla*_NDM_-positive plasmids in Acinetobacter species. The genome sizes of 30 *bla*_NDM_-positive conjugative plasmids belonging to the pNDM-YR7-like clade varied from 39.36 kb to 49.65 kb, with the 25th percentile, median, and 75th percentile being 41.08 kb, 44.56 kb, and 47.27 kb, respectively (Fig. S4). The GC content of the 30 pNDM-YR7-like plasmids ranged from 37.07% to 41.01%, with a median GC content of 38.63% (25th percentile = 38.31%; 75th percentile = 40.79%) (Fig. S4).

The most common species of Acinetobacter containing the *bla*_NDM_-positive conjugative plasmids was A. baumannii, followed by A. lwoffii and *A. indicus* (Fig. 2 and Fig. S5). Notably, pNDM-YR7 in our study is the first report of the *bla*_NDM_-positive conjugative plasmid existing in a strain of A. junii (Fig. 2 and Fig. S5).

### Genetic contexts of *bla*_NDM_ of the 30 pNDM-YR7-like plasmids.

We observed that all 30 *bla*_NDM_-positive conjugative plasmids of the pNDM-YR7-like clade carry only two acquired ARGs, *bla*_NDM_ (*bla*_NDM-14_ in the A. lwoffii plasmid pNDM-JN01 and *bla*_NDM-1_ in the other 29 plasmids) and *aph(3′)-VI*, the former encoding carbapenemase and the latter encoding resistance to aminoglycosides (Fig. S6). For the A. baumannii plasmid pNDM-AB, except for *bla*_NDM-1_ and *aph(3′)-VI*, the *msr(E)*-*mph(E)* operon conferring resistance to macrolides was also identified in the genome of plasmid pNDM-AB (Fig. S6).

**FIG 4:**
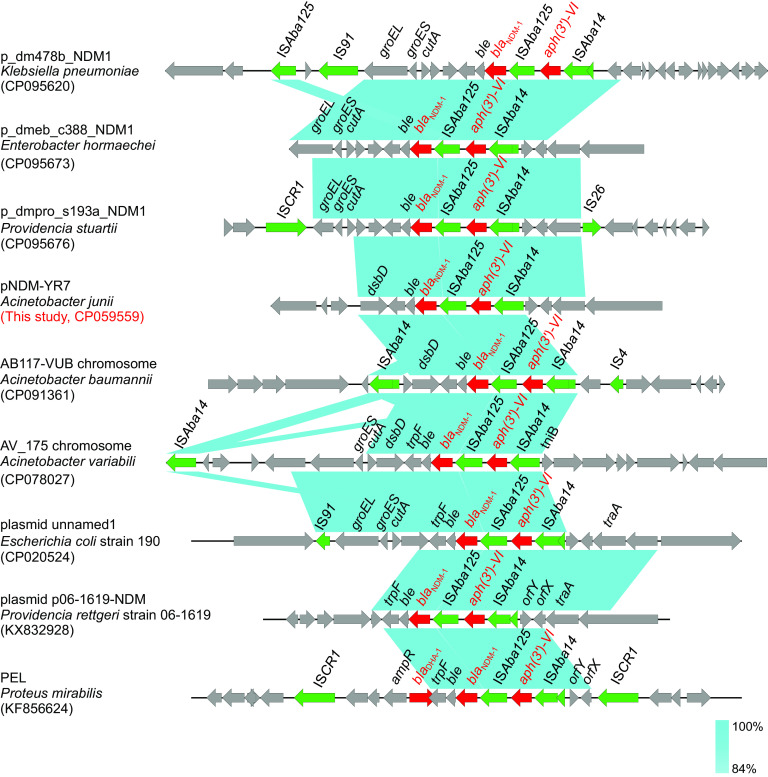
Comparison of the gene cluster embracing the *bla*_NDM_ [IS*Aba14*-*aph(3′)-VI*-IS*Aba125*-*bla*_NDM_-*ble*] carried by the plasmid pNDM-YR7 with other Acinetobacter genomes and other pathogenic bacteria outside this bacterial genus.

Based on the genetic context of bla_NDM-1_ and *aph(3′)-VI* on the plasmid pNDM-YR7, we analyzed those of 29 other pNDM-YR7-like plasmids. The results indicated that the structure IS*Aba14*-*aph(3′)-VI*-IS*Aba125*-*bla*_NDM_-*ble* existed in all 30 *bla*_NDM_-positive conjugative plasmids classified into the pNDM-YR7-like clade (Fig. 3). Based on the conserved region IS*Aba14*-*aph(3′)-VI*-IS*Aba125*-*bla*_NDM_-*ble* of plasmid pNDM-YR7, the BLAST search hit from the nr database of GenBank showed that the structure was present not only in the Acinetobacter plasmids but also in the chromosomes of Acinetobacter (Fig. 4). Moreover, the structure IS*Aba14*-*aph(3′)-VI*-IS*Aba125*-*bla*_NDM_-*ble* was also widely present in the plasmids from K. pneumoniae, Escherichia coli, Enterobacter hormaechei, Providencia rettgeri, Providencia stuartii, Citrobacter freundii, Citrobacter werkmanii, Proteus mirabilis, and others (Fig. 4 and Table S3).

## DISCUSSION

NDM-type carbapenemases are a rapidly emerging and troublesome family of β-lactamases, and NDM-producing Acinetobacter species have been recently reported in many countries, especially the main type, NDM-1 (6, 7). Conjugative plasmids have been highlighted as important vehicles for the dissemination of ARGs (25). To characterize the conjugative plasmids bearing *bla*_NDM_ genes in Acinetobacter species, we systematically compared the profiles of resistance determinants, conjugative transfer regions, and the genetic features associated with *bla*_NDM_ of the 62 predicted *bla*_NDM_-positive plasmids, which were selected from 1,191 plasmids of Acinetobacter species from the GenBank RefSeq database.

For the 62 *bla*_NDM_-positive plasmids of Acinetobacter species, the most common species bearing the *bla*_NDM_-positive plasmids was A. baumannii, accounting for more than one-fourth of all the *bla*_NDM_-positive plasmids of Acinetobacter species identified in this study (16 *bla*_NDM_-positive plasmids). The first report of an NDM-producing clinical strain in Acinetobacter species in the world was a *bla*_NDM-1_-positive A. baumannii strain found in India (8). In China, the first reported *bla*_NDM-1_-positive clinical isolates were also A. baumannii isolates, which were identified in four different provinces in 2010 (12). In our study, other Acinetobacter species bearing *bla*_NDM_-positive plasmids were *A. indicus* (7 *bla*_NDM_-positive plasmids), A. pittii (6 *bla*_NDM_-positive plasmids), and A. lwoffii (5 *bla*_NDM_-positive plasmids). For A. junii, two strains bearing *bla*_NDM_-positive plasmids were found, including the plasmid pNDM-YR7 from A. junii YR7 collected by our group. Of the 62 *bla*_NDM_-positive plasmids of Acinetobacter species, 19 were found to carry the Rep proteins belong to the Rep_3 superfamily (Pfam: 01051), which were the most common Rep proteins carried by the A. baumannii plasmids (21). The 30 *bla*_NDM_-positive conjugative plasmids in this study replicated using the new Rep protein, which was different from the common Rep proteins, such as Rep_3 superfamily, replicase-PriCT, Rep_1 superfamily, and RepC superfamily (21).

Conjugative plasmids, important vehicles for the dissemination of ARGs, play a central role in facilitating horizontal genetic exchange and therefore promoting the acquisition and spread of antimicrobial resistance genes (16, 25). Here, we attempted to analyze and compare the potential conjugative transfer modules located on the 62 *bla*_NDM_-positive plasmids in Acinetobacter species using the software oriTfinder (26). We identified 30 conjugative plasmids from the 62 *bla*_NDM_-harboring plasmids in Acinetobacter species according to their predicted *oriT* regions, relaxase genes, T4CP genes, and gene clusters for T4SS. The *bla*_NDM-1_ gene was originally discovered in a 180-kb plasmid in K. pneumoniae (2), and later, it was reported to be carried by other plasmids ranging from 50 to 500 kb in various Gram-negative species (3, 27). In this study, we compared the genome series of all 30 pNDM-YR7-like plasmids ranging from 39.36 kb to 49.65 kb with a median size of 44.56 kb (25th percentile = 41.08 kb; 75th percentile = 47.27 kb). The most common species of Acinetobacter containing the *bla*_NDM_-positive conjugative plasmids was A. baumannii, followed by A. lwoffii and *A. indicus*. Notably, the pNDM-YR7 in our study is the first report of the *bla*_NDM_-positive conjugative plasmid present in a strain of A. junii.

Conjugative plasmids typically contain conjugative transfer regions consisting of four conjugative modules (22). In this study, we comprehensively analyzed and compared the four conjugative modules of all 30 *bla*_NDM_-positive conjugative plasmids in Acinetobacter species. All 30 *bla*_NDM_-positive conjugative plasmids were found to contain *oriT* regions similar to that of plasmid pNDM-YR7, which represent a new type of *oriT* not included in the nine main types of plasmid-borne *oriT* recorded by the oriTDB database (https://bioinfo-mml.sjtu.edu.cn/oriTDB/browse_oriT_type_p.php) (26), indicating the specificity of the *bla*_NDM_-positive conjugative plasmids in Acinetobacter species. The oriTDB database recorded eight main relaxase families (https://bioinfo-mml.sjtu.edu.cn/oriTDB/browse_relaxase.php) (26), and in our study, almost all the *bla*_NDM_-positive conjugative plasmids of Acinetobacter species (29 plasmids) were found to contain genes coding for relaxases belonging to the MOB_Q_ family, with the hidden Markov model (HMM) profile of MobA_MobL in their protein sequences. Members of the MOB_Q_ family were found not only in *Proteobacteria* but also in *Firmicutes* and *Cyanobacteria* and were encoded on many different plasmids, not just IncQ plasmids (22). The relaxases R1162, RSF1010, and R3200B, which were independently isolated but essentially identical plasmids, were the prototypes of the MOB_Q_ family (28, 29). T4CPs are essential elements in conjugative T4SSs and are also key elements in many pathogenic T4SSs (30). In this study, all the *bla*_NDM_-positive conjugative plasmids of Acinetobacter species were found to carry the genes encoding T4CPs of the TrwB/TraD subfamily, with the TrwB of plasmid R388 from E. coli as a representative (31). The T4SS gene cluster is predicted by the software oriTfinder via the colocalization of the homologs of at least five core components (26). According to this standard, 29 of the 30 *bla*_NDM_-positive conjugative plasmids of Acinetobacter species were found to contain gene clusters for the VirB-like T4SS, which is by far the best-characterized T4SS (32).

In our study, we found that all 30 *bla*_NDM_-positive conjugative plasmids in Acinetobacter species carried two acquired ARGs, *bla*_NDM_ and *aph(3′)-VI*. In Acinetobacter species, the *bla*_NDM-1_ gene has been reported to be embedded in transposon Tn*125* (33–35), which is a composite transposon bracketed by two copies of the insertion sequence IS*Aba125* oriented in the same direction. However, in this study, we found that *bla*_NDM_ and *aph(3′)-VI* were located on the structure IS*Aba14*-*aph(3′)-VI*-IS*Aba125*-*bla*_NDM_-*ble*. Notably, the genetic context was present not only in all 30 *bla*_NDM_-positive conjugative plasmids of Acinetobacter species but also in the chromosomes of Acinetobacter. Moreover, it was also widely present in the plasmids from various species of *Enterobacterales*, underlining its strong transmissibility.

## MATERIALS AND METHODS

### Isolation and characterization of carbapenem-resistant bacterial strain YR7.

The carbapenem-resistant bacterial strain YR7 was isolated from a chicken meat sample collected from markets in Hangzhou city, Zhejiang Province, in November 2018. It was identified by matrix-assisted laser desorption ionization–time of flight mass spectrometry (MALDI-TOF MS) (Bruker MALDI Biotyper System, Germany) and 16S rRNA gene (rDNA) sequencing. Strain YR7 was tested for PCR amplification of *bla*_NDM_ using specific primers as previously described (36, 37). Antimicrobial susceptibility testing was performed by the broth microdilution method using a Gram-negative panel (Fosun Diagnostics, Shanghai, China). The results were interpreted according to the Clinical and Laboratory Standards Institute (CLSI) documents M100-S27 and M45-A2 (38). Additionally, Etests of meropenem were applied. The A. junii strain BNCC 354128 served as a quality control in this assay. The cell size and morphology of strain YR7 were determined by cold-field emission scanning electron microscopy (Hitachi Regulus 8100) using cells from the exponential growth phase cultured in Luria-Bertani (LB) broth.

### Whole-genome sequencing, assembly, and annotation.

A single colony of strain YR7 was inoculated into 10 mL of LB broth for DNA extraction (QIAprep Spin miniprep kit, Qiagen, Germany). Long-read libraries were prepared (SQK-LSK109 kit; Oxford Nanopore Technologies [ONT]) and sequenced using a Flo-MIN106D R9.4 flow cell on a GridION sequencer. Meanwhile, a short-read library was generated using a NEXTflex DNA sequencing kit (Bioo Scientific, USA) for Illumina sequencing. All the following software systems were run with their default settings. Guppy v3.2.4 software (ONT) was used for base calling and adapter removal. Nanopore reads were assembled *de novo* using Canu v 1.7.11, and the assembly was circularized by Circulator v1.5.1. The genome sequence was corrected by Illumina reads based on Pilon v1.22 software. Furthermore, gene prediction and annotation were performed according to the NCBI Prokaryotic Genome Annotation Pipeline (39).

### Bacterial plasmid sequences from the NCBI RefSeq database.

Bacterial plasmid genomic sequences without duplicates were downloaded from the National Center for Biotechnology Information (NCBI) RefSeq database (40) (https://ftp.ncbi.nih.gov/refseq/release/plasmid/), and the download date was 14 July 2021. We extracted 1,212 plasmids belonging to Acinetobacter species, including 1,191 sequences termed plasmids and 21 sequences termed only complete coding sequences (CDSs). We applied the 1,191 plasmids of Acinetobacter species for further study (see Table S1 in the supplemental material).

### Identification of *bla*_NDM_-positive plasmids of Acinetobacter species.

The potential β-lactamase genes of plasmids (in FASTA DNA format) from Acinetobacter species were identified using ResFinder software version 4.1 (41) with a minimum identity of 90% and a minimum coverage of 60%. The term “*bla*_NDM_” was used to search the “Antimicrobial resistance gene” list of the ResFinder results to screen the *bla*_NDM_-positive plasmids of Acinetobacter species. If the exact variant of *bla*_NDM_ was not determined using the ResFinder software, it was submitted to the CARD database (https://card.mcmaster.ca) (42) for further analysis.

### Bioinformatics analysis of the *bla*_NDM_-positive plasmids of Acinetobacter species.

The files in GenBank format of the *bla*_NDM_-positive plasmids of Acinetobacter species were downloaded in batch via the Bio::DB::GenBank and Bio::SeqIO modules of Bioperl. The files in GenBank format of the *bla*_NDM_-positive plasmids of Acinetobacter species were analyzed in batch using the software oriTfinder (26) (local version) to determine the presence/absence of *oriT*s, relaxase genes, T4CP genes, and gene clusters for T4SS (at least five core genes). In addition, the types of *oriT*s, relaxase genes, and T4CP genes of the plasmids were identified based on the oriTDB database (https://bioinfo-mml.sjtu.edu.cn/oriTDB/) (26), and the types of gene clusters for T4SS of the plasmids were classified via the SecReT4 database (https://bioinfo-mml.sjtu.edu.cn/SecReT4/) (43).

Replication initiation protein (Rep) genes of the plasmids of Acinetobacter species were identified using hmmscan of the HMMER3 (44) with the following HMM profiles: Rep_3 (PF01051), replicase (PF03090), Rep_1 (PF01446), and RepC (PF06504) (20, 21).

Phylogenetic cladograms based on the presence/absence of orthologous gene families of all *bla*_NDM_-positive plasmids of Acinetobacter species were performed in this study. Files containing protein sequences were extracted from the files in GenBank format using the Bio::SeqIO module of Bioperl. A binary protein presence/absence matrix was created using OrthoFinder (45) with DIAMOND for sequence similarity searches, and then, the hierarchical cluster result was visually displayed by iTOL software (https://itol.embl.de/) (46).

The bacterial insertion sequences of the *bla*_NDM_-positive plasmids of Acinetobacter species were explored using ISfinder (47) and VRprofile2 (48). Comparisons among the genetic contexts of *bla*_NDM_ of the plasmids were performed using the BLAST Ring Image Generator (BRIG) (49) and Easyfig (50).

### S1 pulsed-field gel electrophoresis and Southern blotting.

S1 pulsed-field gel electrophoresis and Southern blotting were carried out as previously described (51, 52).

### Data availability.

The complete genome sequences of Acinetobacter junii strain YR7 were deposited in GenBank with accession numbers CP059558 to CP059559.
