# Supplementary material for: In Silico Characterization of blaNDM-Harboring Conjugative Plasmids in Acinetobacter Species
Source: Microbiol Spectr. 2022 Oct 27;10(6):e02102-22. doi: 10.1128/spectrum.02102-22 (PMC9769834; doi:10.1128/spectrum.02102-22)
Supplement: Supplemental file 1 — Fig. S1-S6; Tables S1-S3. Download spectrum.02102-22-s0001.pdf, PDF file, 2.1 MB [file spectrum.02102-22-s0001.pdf]

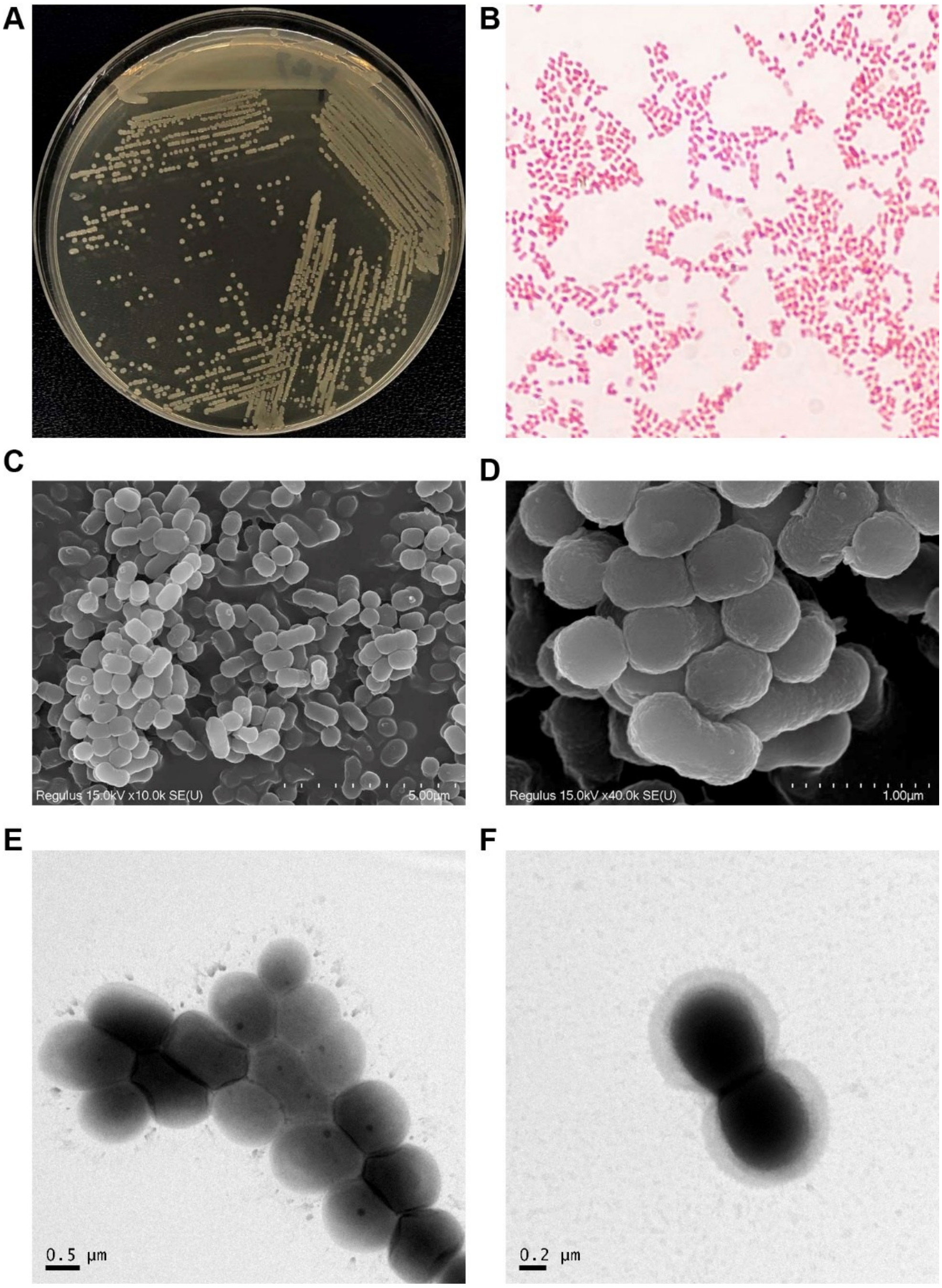

Figure S1. Microbial characterization of the strain YR7. (A) Colonial phenotype of the strain YR7 on MHA plates. (B) Gram-staining analysis of the strain YR7. (C&D) Scanning electron micrographs of the cell surface of strain YR7. (E&F) Transmission electron micrographs of the strain YR7.

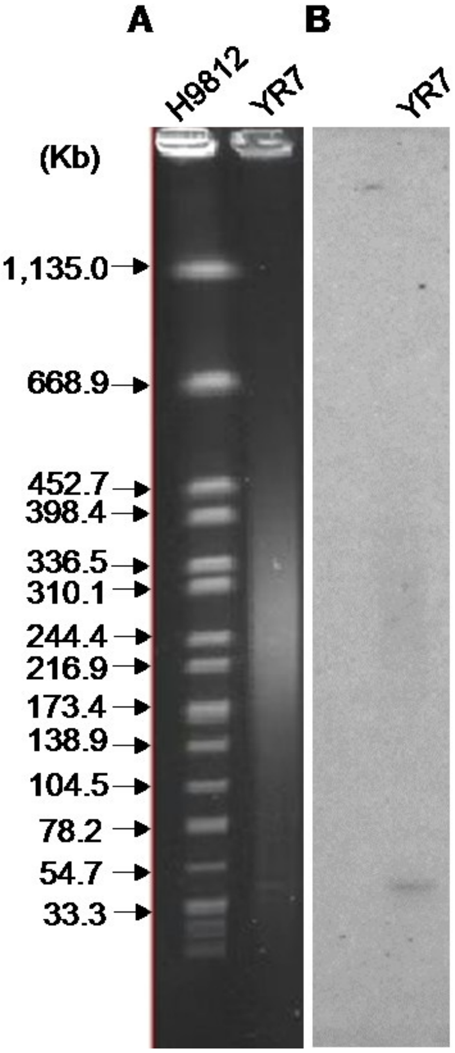

Figure S2. The plasmid size of pNDM-YR7 using an S1-PFGE (A) and confirmation the presence of *bla*NDM on plasmid using southern blotting (B).

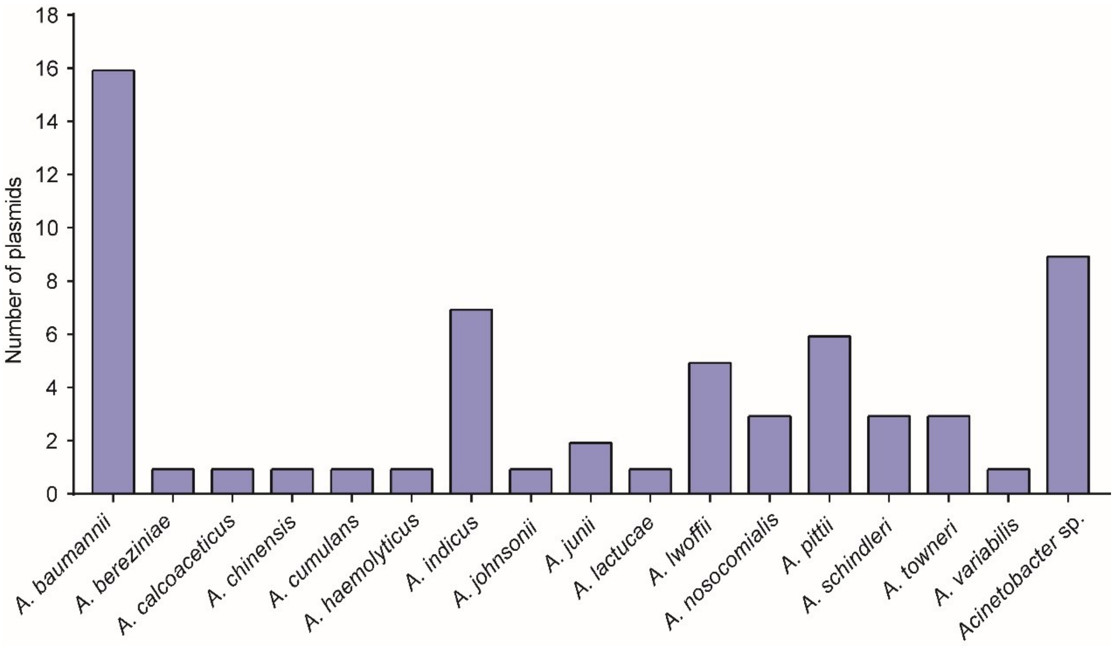

Figure S3. Histogram about number of plasmids distributed in different species for the 62 *bla*$_{\mathrm{NDM}}$-positive plasmids in *Acinetobacter* species.

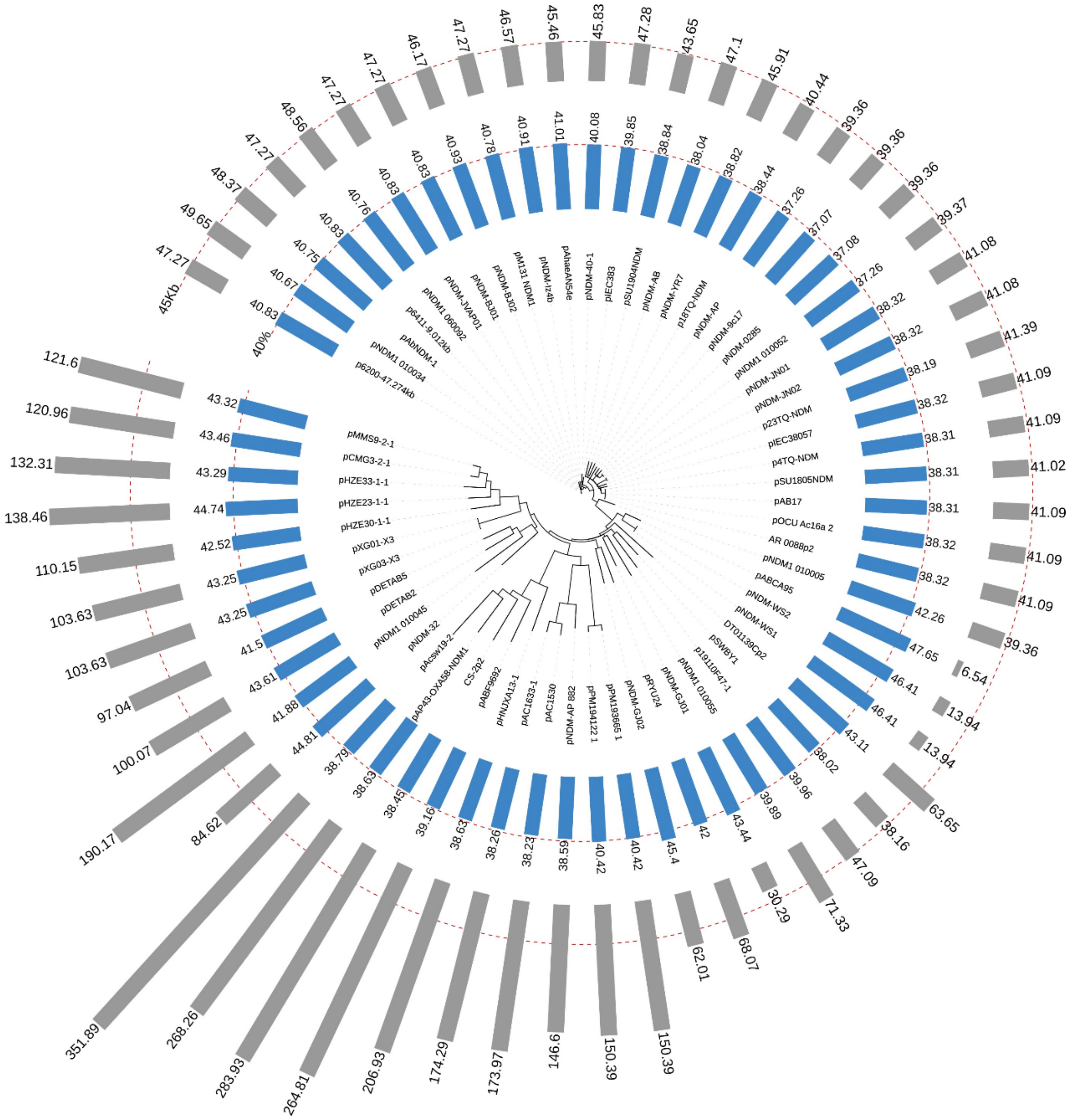

Figure S4. Length distribution of length and GC content of the 62 *bla*$_{\mathrm{NDM}}$-positive plasmids from 1191 plasmids of *Acinetobacter* species. (A) Length distribution of the 30 *bla*$_{\mathrm{NDM}}$-positive plasmids in *Acinetobacter* species. (B) GC content distribution of the 30 *bla*$_{\mathrm{NDM}}$-positive plasmids in *Acinetobacter* species.

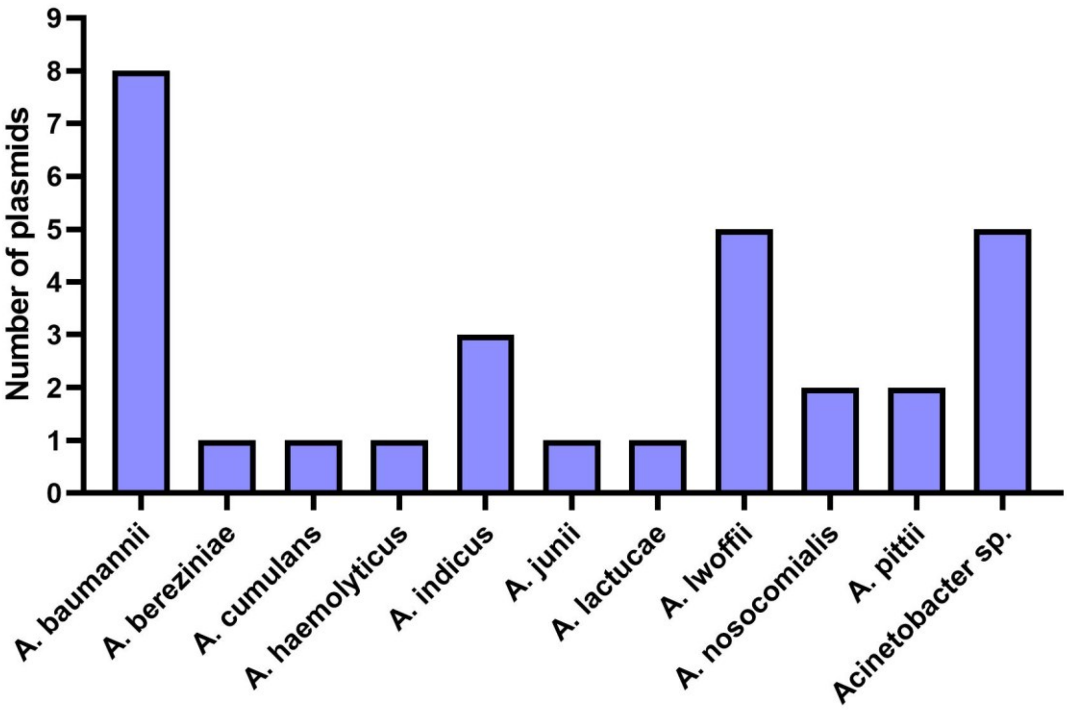

Figure S5. Histogram about number of plasmids distributed in different species for the 30 *bla*$_{\mathrm{NDM}}$-positive conjugative plasmids in *Acinetobacter* species.

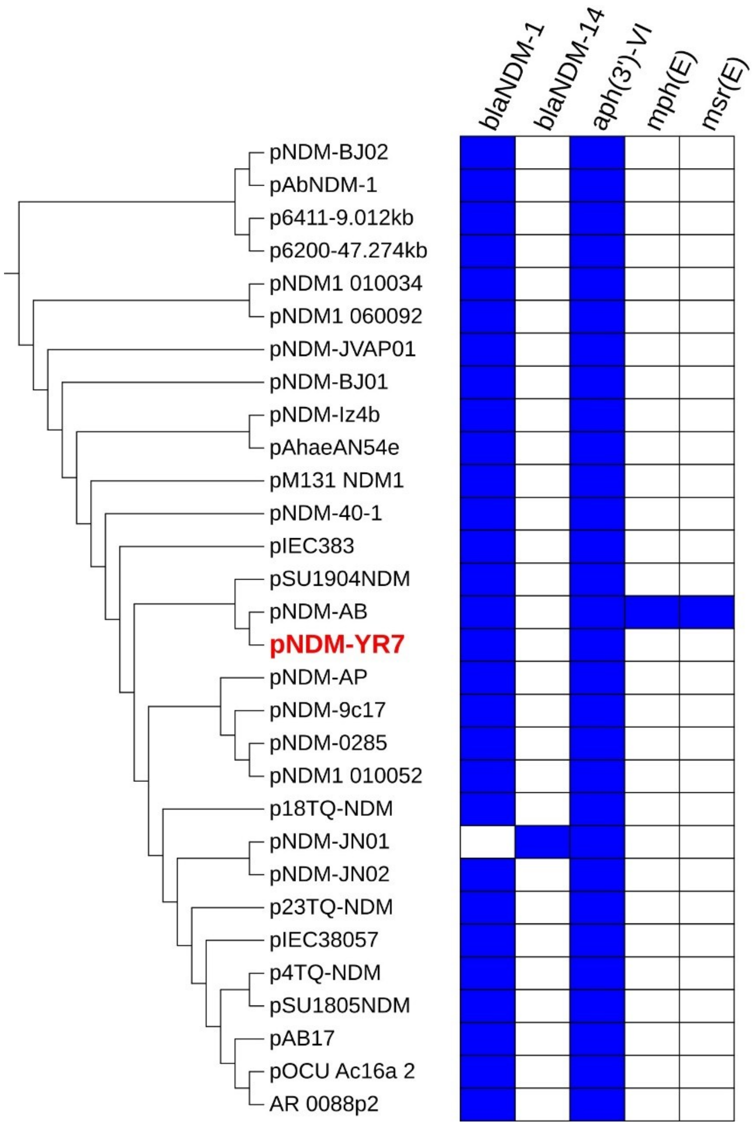

Figure S6. An overview of the ResFinder-facilitated detection of acquired antimicrobial resistance genes (ARGs) towards the 30 *bla*$_{\mathrm{NDM}}$-positive conjugatives plasmids in *Acinetobacter* species.

Table legends：

Table S1. Information of 1,191 plasmids from *Acinetobacter* species downloaded from the NCBI RefSeq database.

Table S2. Details of the results of software ResFinder based on the 1,191 plasmids of *Acinetobacter* species from the GenBank RefSeq database.

Table S3. Results of alignments of the genetic contexts associated with bla_{NDM-1} and *aph(3')-VI* of plasmid pNDM-YR7 in *A. junii* YR7 against the nr database of GenBank (Top100 results).

#ID	Accession number	Description
1	NC_000923	Acinetobacter sp. SUN plasmid pRAY, complete sequence
2	NC_002760	Acinetobacter sp. EB104 plasmid pAC450, complete sequence
3	NC_006877	Acinetobacter baumannii plasmid pMAC, complete sequence
4	NC_010309	Acinetobacter venetianus plasmid pAV1, complete sequence
5	NC_010310	Acinetobacter venetianus plasmid pAV2, complete sequence
6	NC_010401	Acinetobacter baumannii AYE plasmid p1ABAYE, complete sequence
7	NC_010402	Acinetobacter baumannii AYE plasmid p2ABAYE, complete sequence
8	NC_010403	Acinetobacter baumannii AYE plasmid p4ABAYE, complete sequence
9	NC_010404	Acinetobacter baumannii AYE plasmid p3ABAYE, complete sequence
10	NC_010481	Acinetobacter baumannii plasmid pABIR, complete sequence
11	NC_010605	Acinetobacter baumannii ACICU plasmid pACICU1, complete sequence
12	NC_010606	Acinetobacter baumannii ACICU plasmid pACICU2, complete sequence
13	NC_011585	Acinetobacter baumannii AB0057 plasmid pAB0057, complete sequence
14	NC_012813	Acinetobacter baumannii plasmid pABVA01, complete sequence
15	NC_013056	Acinetobacter calcoaceticus plasmid pMMCU1, complete sequence
16	NC_013277	Acinetobacter baumannii plasmid pMMA2, complete sequence
17	NC_013506	Acinetobacter baumannii plasmid pMMCU2, complete sequence
18	NC_016977	Acinetobacter baumannii plasmid pTS236, complete sequence
19	NC_017163	Acinetobacter baumannii 1656-2 plasmid ABKp1, complete sequence
20	NC_017164	Acinetobacter baumannii 1656-2 plasmid ABKp2, complete sequence
21	NC_017848	Acinetobacter baumannii MDR-TJ plasmid pABTJ1, complete sequence
22	NC_019199	Acinetobacter baumannii plasmid pMMCU3, complete sequence
23	NC_019268	Acinetobacter lwoffii plasmid pNDM-BJ01, complete sequence
24	NC_019280	Acinetobacter baumannii plasmid pMMD, complete sequence
25	NC_019281	Acinetobacter lwoffii plasmid pNDM-BJ02, complete sequence
26	NC_019311	Acinetobacter baumannii plasmid pRAY*-v1, complete sequence
27	NC_019345	Acinetobacter baumannii plasmid pRAY*-v2, complete sequence
28	NC_019359	Acinetobacter baumannii plasmid pAB120, complete sequence
29	NC_019985	Acinetobacter baumannii ZW85-1 plasmid pAbNDM-1, complete sequence
30	NC_020524	Acinetobacter baumannii MDR-TJ plasmid pABTJ2, complete sequence
31	NC_020525	Acinetobacter baumannii D1279779 plasmid pD1279779, complete sequence
32	NC_020818	Acinetobacter baumannii plasmid pNDM-AB, complete sequence

33	NC_021489	Acinetobacter baumannii plasmid pAB-NCGM253, complete sequence
34	NC_021727	Acinetobacter baumannii BJAB07104 plasmid p1BJAB07104, complete sequence
35	NC_021728	Acinetobacter baumannii BJAB07104 plasmid p2BJAB07104, complete sequence
36	NC_021730	Acinetobacter baumannii BJAB0868 plasmid p1BJAB0868, complete sequence
37	NC_021731	Acinetobacter baumannii BJAB0868 plasmid p2BJAB0868, complete sequence
38	NC_021732	Acinetobacter baumannii BJAB0868 plasmid p3BJAB0868, complete sequence
39	NC_021734	Acinetobacter baumannii BJAB0715 plasmid pBJAB0715, complete sequence
40	NC_022565	Acinetobacter baumannii 107m plasmid ABIBUN107mP1, complete sequence
41	NC_023031	Acinetobacter baumannii ZW85-1 plasmid ZW85p2, complete sequence
42	NC_023280	Acinetobacter nosocomialis plasmid pRAY*-v3, complete sequence
43	NC_023281	Acinetobacter nosocomialis plasmid pAB49-v1, complete sequence
44	NC_023322	Acinetobacter bereziniae plasmid pNDM-40-1, complete sequence
45	NC_024959	Acinetobacter calcoaceticus plasmid pNDM-WS2, complete sequence
46	NC_024999	Acinetobacter junii plasmid pNDM-WS1, complete sequence
47	NC_025000	Acinetobacter lwoffii plasmid pNDM-Iz4b, complete sequence
48	NC_025068	Acinetobacter baumannii plasmid pRAY*, complete sequence
49	NC_025104	Acinetobacter baumannii plasmid pAb-G7-2, complete sequence
50	NC_025109	Acinetobacter baumannii plasmid pA85-3, complete sequence
51	NC_025110	Acinetobacter baumannii plasmid pAB-G7-1, complete sequence
52	NC_025111	Acinetobacter baumannii plasmid pD72-2, complete sequence
53	NC_025116	Acinetobacter sp. M131 plasmid pM131_NDM1, complete sequence
54	NC_025117	Acinetobacter sp. M131 plasmid pM131-11, complete sequence
55	NC_025118	Acinetobacter sp. M131 plasmid pM131-9, complete sequence
56	NC_025119	Acinetobacter sp. M131 plasmid pM131-8, complete sequence
57	NC_025120	Acinetobacter sp. M131 plasmid pM131-6, complete sequence
58	NC_025121	Acinetobacter sp. M131 plasmid pM131-4, complete sequence
59	NC_025122	Acinetobacter sp. M131 plasmid pM131-3, complete sequence
60	NC_025137	Acinetobacter pittii plasmid pMS32-3, complete sequence
61	NC_025168	Acinetobacter baumannii plasmid AbATCC329, complete sequence
62	NC_025169	Acinetobacter sp. M131 plasmid pM131-10, complete sequence
63	NC_025170	Acinetobacter sp. M131 plasmid pM131-7, complete sequence
64	NC_025171	Acinetobacter sp. M131 plasmid pM131-5, complete sequence

65	NC_025172	Acinetobacter sp. M131 plasmid pM131-2, complete sequence
66	NC_025173	Acinetobacter pittii plasmid pMS32-1, complete sequence
67	NZ_AP014650	Acinetobacter baumannii strain IOMTU433 plasmid pIOMTU433, complete sequence
68	NZ_AP018825	Acinetobacter ursingii strain M3 plasmid pAURM-1, complete sequence
69	NZ_AP018826	Acinetobacter ursingii strain M3 plasmid pAURM-2, complete sequence
70	NZ_AP019741	Acinetobacter radioresistens DSM 6976 = NBRC 102413 = CIP 103788 strain NBRC 102413 plasmid pARA1, complete sequence
71	NZ_AP019742	Acinetobacter radioresistens DSM 6976 = NBRC 102413 = CIP 103788 strain NBRC 102413 plasmid pARA2, complete sequence
72	NZ_AP019743	Acinetobacter radioresistens DSM 6976 = NBRC 102413 = CIP 103788 strain NBRC 102413 plasmid pARA3, complete sequence
73	NZ_AP019744	Acinetobacter radioresistens DSM 6976 = NBRC 102413 = CIP 103788 strain NBRC 102413 plasmid pARA4, complete sequence
74	NZ_AP019745	Acinetobacter radioresistens DSM 6976 = NBRC 102413 = CIP 103788 strain NBRC 102413 plasmid pARA5, complete sequence
75	NZ_AP019746	Acinetobacter radioresistens DSM 6976 = NBRC 102413 = CIP 103788 strain NBRC 102413 plasmid pARA6, complete sequence
76	NZ_AP019747	Acinetobacter radioresistens DSM 6976 = NBRC 102413 = CIP 103788 strain NBRC 102413 plasmid pARA7, complete sequence
77	NZ_AP019748	Acinetobacter radioresistens DSM 6976 = NBRC 102413 = CIP 103788 strain NBRC 102413 plasmid pARA8, complete sequence
78	NZ_AP021937	Acinetobacter pittii strain WP2-W18-ESBL-11 plasmid pWP2-W18-ESBL-11_1, complete sequence
79	NZ_AP021938	Acinetobacter pittii strain WP2-W18-ESBL-11 plasmid pWP2-W18-ESBL-11_2, complete sequence
80	NZ_AP021939	Acinetobacter pittii strain WP2-W18-ESBL-11 plasmid pWP2-W18-ESBL-11_3, complete sequence
81	NZ_AP022239	Acinetobacter baumannii strain WP8-W18-ESBL-11 plasmid pWP8-W18-ESBL-11_1, complete sequence
82	NZ_AP022240	Acinetobacter baumannii strain WP8-W18-ESBL-11 plasmid pWP8-W18-ESBL-11_2, complete sequence
83	NZ_AP022303	Acinetobacter sp. BEC1-S18-ESBL-01 plasmid pBEC1-S18-ESBL-01_1, complete sequence
84	NZ_AP022304	Acinetobacter sp. BEC1-S18-ESBL-01 plasmid pBEC1-S18-ESBL-01_2, complete sequence
85	NZ_AP023078	Acinetobacter baumannii strain OCU_Ac16a plasmid pOCU_Ac16a_1, complete sequence
86	NZ_AP023079	Acinetobacter baumannii strain OCU_Ac16a plasmid pOCU_Ac16a_2, complete sequence
87	NZ_AP023080	Acinetobacter baumannii strain OCU_Ac16a plasmid pOCU_Ac16a_3, complete sequence
88	NZ_CP006964	Acinetobacter baumannii PKAB07 strain AB07 plasmid pPKAB07, complete sequence
89	NZ_CP007713	Acinetobacter baumannii LAC-4 plasmid pABLAC1, complete sequence
90	NZ_CP007714	Acinetobacter baumannii LAC-4 plasmid pABLAC2, complete sequence

91	NZ_CP008707	Acinetobacter baumannii strain AB5075-UW plasmid p1AB5075, complete sequence
92	NZ_CP008708	Acinetobacter baumannii strain AB5075-UW plasmid p2AB5075, complete sequence
93	NZ_CP008709	Acinetobacter baumannii strain AB5075-UW plasmid p3AB5075, complete sequence
94	NZ_CP008850	Acinetobacter baumannii strain AC29 plasmid pAC29a, complete sequence
95	NZ_CP008851	Acinetobacter baumannii strain AC29 plasmid pAC29b, complete sequence
96	NZ_CP010351	Acinetobacter johnsonii XBB1 plasmid pXBB1-9, complete sequence
97	NZ_CP010352	Acinetobacter johnsonii XBB1 plasmid pXBB1-1, complete sequence
98	NZ_CP010353	Acinetobacter johnsonii XBB1 plasmid pXBB1-2, complete sequence
99	NZ_CP010354	Acinetobacter johnsonii XBB1 plasmid pXBB1-3, complete sequence
100	NZ_CP010355	Acinetobacter johnsonii XBB1 plasmid pXBB1-4, complete sequence
101	NZ_CP010356	Acinetobacter johnsonii XBB1 plasmid pXBB1-5, complete sequence
102	NZ_CP010357	Acinetobacter johnsonii XBB1 plasmid pXBB1-6, complete sequence
103	NZ_CP010358	Acinetobacter johnsonii XBB1 plasmid pXBB1-8, complete sequence
104	NZ_CP010369	Acinetobacter nosocomialis strain 6411 plasmid p6411-89.111kb, complete sequence
105	NZ_CP010370	Acinetobacter nosocomialis strain 6411 plasmid p6411-9.012kb, complete sequence
106	NZ_CP010398	Acinetobacter baumannii strain 6200 plasmid p6200-114.848kb, complete sequence
107	NZ_CP010399	Acinetobacter baumannii strain 6200 plasmid p6200-47.274kb, complete sequence
108	NZ_CP010400	Acinetobacter baumannii strain 6200 plasmid p6200-9.327kb, complete sequence
109	NZ_CP010780	Acinetobacter baumannii strain XH386 plasmid pAB386, complete sequence
110	NZ_CP010782	Acinetobacter baumannii strain A1 plasmid pA1-1, complete sequence
111	NZ_CP010903	Acinetobacter nosocomialis strain 6411 plasmid p6411-66.409kb, complete sequence
112	NZ_CP012005	Acinetobacter baumannii strain ATCC 17978-mff plasmid pAB3, complete sequence
113	NZ_CP012007	Acinetobacter baumannii strain Ab04-mff plasmid pAB04-1, complete sequence
114	NZ_CP012008	Acinetobacter baumannii strain Ab04-mff plasmid pAB04-2, complete sequence
115	NZ_CP012953	Acinetobacter baumannii strain D36 plasmid pD36-1 clone GC1, complete sequence
116	NZ_CP012954	Acinetobacter baumannii strain D36 plasmid pRAY* (pD36-2) clone GC1, complete sequence
117	NZ_CP012955	Acinetobacter baumannii strain D36 plasmid pD36-3 clone GC1, complete sequence
118	NZ_CP012956	Acinetobacter baumannii strain D36 plasmid pD36-4 clone GC1, complete sequence
119	NZ_CP013925	Acinetobacter baumannii strain KBN10P02143 plasmid pKBN10P02143, complete sequence
120	NZ_CP014216	Acinetobacter baumannii strain YU-R612 plasmid unnamed1, complete sequence
121	NZ_CP014217	Acinetobacter baumannii strain YU-R612 plasmid unnamed2, complete sequence
122	NZ_CP014478	Acinetobacter pittii strain AP_882 plasmid pNDM-AP_882, complete sequence

123	NZ_CP014479	Acinetobacter pittii strain AP_882 plasmid pOXA58-AP_882, complete sequence
124	NZ_CP015111	Acinetobacter sp. TGL-Y2 plasmid unnamed1, complete sequence
125	NZ_CP015112	Acinetobacter sp. TGL-Y2 plasmid unnamed2, complete sequence
126	NZ_CP015122	Acinetobacter baumannii strain ab736 plasmid unnamed, complete sequence
127	NZ_CP015146	Acinetobacter pittii strain IEC338SC plasmid pIEC338SCOX, complete sequence
128	NZ_CP015147	Acinetobacter pittii strain IEC338SC plasmid pIEC338SC2, complete sequence
129	NZ_CP015148	Acinetobacter pittii strain IEC338SC plasmid pIEC338SC3, complete sequence
130	NZ_CP015365	Acinetobacter baumannii strain 3207 plasmid pAba3207a, complete sequence
131	NZ_CP015366	Acinetobacter baumannii strain 3207 plasmid pAba3207b, complete sequence
132	NZ_CP015484	Acinetobacter baumannii strain ORAB01 plasmid pORAB01-1, complete sequence
133	NZ_CP015485	Acinetobacter baumannii strain ORAB01 plasmid pORAB01-2, complete sequence
134	NZ_CP015486	Acinetobacter baumannii strain ORAB01 plasmid pORAB01-3, complete sequence
135	NZ_CP015595	Acinetobacter sp. NCu2D-2 plasmid unnamed, complete sequence
136	NZ_CP015616	Acinetobacter schindleri strain ACE plasmid p1AsACE, complete sequence
137	NZ_CP015617	Acinetobacter schindleri strain ACE plasmid p2AsACE, complete sequence
138	NZ_CP015618	Acinetobacter schindleri strain ACE plasmid p3AsACE, complete sequence
139	NZ_CP015619	Acinetobacter schindleri strain ACE plasmid p4AsACE, complete sequence
140	NZ_CP015620	Acinetobacter schindleri strain ACE plasmid p5AsACE, complete sequence
141	NZ_CP015621	Acinetobacter schindleri strain ACE plasmid p6AsACE, complete sequence
142	NZ_CP016296	Acinetobacter baumannii strain CMC-CR-MDR-Ab4 plasmid pCMCVTAb1-Ab4, complete sequence
143	NZ_CP016297	Acinetobacter baumannii strain CMC-CR-MDR-Ab4 plasmid pCMCVTAb2-Ab4, complete sequence
144	NZ_CP016299	Acinetobacter baumannii strain CMC-MDR-Ab59 plasmid pCMCVTAb1-Ab59, complete sequence
145	NZ_CP016301	Acinetobacter baumannii strain CMC-CR-MDR-Ab66 plasmid pCMCVTAb1-Ab66, complete sequence
146	NZ_CP016302	Acinetobacter baumannii strain CMC-CR-MDR-Ab66 plasmid pCMCVTAb2-Ab66, complete sequence
147	NZ_CP016897	Acinetobacter soli strain GFJ2 plasmid pGFJ1, complete sequence
148	NZ_CP016898	Acinetobacter soli strain GFJ2 plasmid pGFJ2, complete sequence
149	NZ_CP016899	Acinetobacter soli strain GFJ2 plasmid pGFJ3, complete sequence
150	NZ_CP016900	Acinetobacter soli strain GFJ2 plasmid pGFJ4, complete sequence
151	NZ_CP016901	Acinetobacter soli strain GFJ2 plasmid pGFJ5, complete sequence
152	NZ_CP016902	Acinetobacter soli strain GFJ2 plasmid pGFJ6, complete sequence

153	NZ_CP016903	Acinetobacter soli strain GFJ2 plasmid pGFJ7, complete sequence
154	NZ_CP017643	Acinetobacter baumannii strain KAB01 plasmid unnamed, complete sequence
155	NZ_CP017645	Acinetobacter baumannii strain KAB02 plasmid unnamed, complete sequence
156	NZ_CP017647	Acinetobacter baumannii strain KAB03 plasmid unnamed, complete sequence
157	NZ_CP017649	Acinetobacter baumannii strain KAB04 plasmid unnamed, complete sequence
158	NZ_CP017651	Acinetobacter baumannii strain KAB05 plasmid unnamed, complete sequence
159	NZ_CP017653	Acinetobacter baumannii strain KAB06 plasmid unnamed, complete sequence
160	NZ_CP017655	Acinetobacter baumannii strain KAB07 plasmid pKAB07, complete sequence
161	NZ_CP017657	Acinetobacter baumannii strain KAB08 plasmid unnamed, complete sequence
162	NZ_CP017939	Acinetobacter pittii strain YMC2010/8/T346 plasmid unnamed1, complete sequence
163	NZ_CP018141	Acinetobacter larvae strain BRTC-1 plasmid pRW1, complete sequence
164	NZ_CP018142	Acinetobacter larvae strain BRTC-1 plasmid pRW2, complete sequence
165	NZ_CP018144	Acinetobacter baumannii strain HRAB-85 plasmid unnamed, complete sequence
166	NZ_CP018255	Acinetobacter baumannii strain AF-401 plasmid pAF-401, complete sequence
167	NZ_CP018257	Acinetobacter baumannii strain AF-673 plasmid pAF-673, complete sequence
168	NZ_CP018261	Acinetobacter haemolyticus strain XH900 plasmid pXH901, complete sequence
169	NZ_CP018333	Acinetobacter baumannii strain A1296 isolate A1296 plasmid pA1296_1, complete sequence
170	NZ_CP018334	Acinetobacter baumannii strain A1296 isolate A1296 plasmid pA1296_2, complete sequence
171	NZ_CP018422	Acinetobacter baumannii strain XDR-BJ83 isolate male patient plasmid pBJ83, complete sequence
172	NZ_CP018678	Acinetobacter baumannii strain LAC4 plasmid pALAC4-1, complete sequence
173	NZ_CP018679	Acinetobacter baumannii strain LAC4 plasmid pALAC4-2, complete sequence
174	NZ_CP018862	Acinetobacter baumannii strain 11510 plasmid pAba11510a
175	NZ_CP018872	Acinetobacter haemolyticus strain TJS01 plasmid pAHTJS1, complete sequence
176	NZ_CP018873	Acinetobacter haemolyticus strain TJS01 plasmid pAHTJS2, complete sequence
177	NZ_CP018910	Acinetobacter pittii strain XJ88 plasmid unnamed1, complete sequence
178	NZ_CP019115	Acinetobacter baumannii strain MDR-CQ plasmid pMDR-CQ, complete sequence
179	NZ_CP019144	Acinetobacter lwoffii strain ZS207 plasmid pmZS sequence
180	NZ_CP019145	Acinetobacter lwoffii strain ZS207 plasmid pZS-3, complete sequence
181	NZ_CP019146	Acinetobacter lwoffii strain ZS207 plasmid pZS-6, complete sequence
182	NZ_CP019147	Acinetobacter lwoffii strain ZS207 plasmid pZS-7, complete sequence
183	NZ_CP019148	Acinetobacter lwoffii strain ZS207 plasmid pZS-8, complete sequence
184	NZ_CP019149	Acinetobacter lwoffii strain ZS207 plasmid pZS-9, complete sequence

185	NZ_CP019150	Acinetobacter lwoffii strain ZS207 plasmid pZS-11, complete sequence
186	NZ_CP019151	Acinetobacter lwoffii strain ZS207 plasmid pZS-13, complete sequence
187	NZ_CP019152	Acinetobacter lwoffii strain ZS207 plasmid pZS-20, complete sequence
188	NZ_CP019218	Acinetobacter baumannii strain XH731 plasmid pXH731, complete sequence
189	NZ_CP020001	Acinetobacter calcoaceticus strain CA16 plasmid pCA16, complete sequence
190	NZ_CP020573	Acinetobacter baumannii strain 15A5 plasmid p15A5_1, complete sequence
191	NZ_CP020575	Acinetobacter baumannii strain 15A5 plasmid p15A5_2, complete sequence
192	NZ_CP020576	Acinetobacter baumannii strain SSA12 plasmid pSSA12_2, complete sequence
193	NZ_CP020577	Acinetobacter baumannii strain SSA12 plasmid pSSA12_1, complete sequence
194	NZ_CP020580	Acinetobacter baumannii strain SSMA17 plasmid pSSMA17_1, complete sequence
195	NZ_CP020582	Acinetobacter baumannii strain JBA13 plasmid pJBA13_1, complete sequence
196	NZ_CP020583	Acinetobacter baumannii strain JBA13 plasmid pJBA13_2, complete sequence
197	NZ_CP020585	Acinetobacter baumannii strain CBA7 plasmid pCBA7_1, complete sequence
198	NZ_CP020587	Acinetobacter nosocomialis strain SSA3 plasmid pSSA3_1, complete sequence
199	NZ_CP020589	Acinetobacter baumannii strain 15A34 plasmid p15A34_1, complete sequence
200	NZ_CP020593	Acinetobacter baumannii strain USA2 plasmid pUSA2_1, complete sequence
201	NZ_CP020594	Acinetobacter baumannii strain USA15 plasmid pUSA15_1, complete sequence
202	NZ_CP020596	Acinetobacter baumannii strain HWBA8 plasmid pHWBA8_1, complete sequence
203	NZ_CP021322	Acinetobacter baumannii strain XH731 plasmid pXH731, complete sequence
204	NZ_CP021327	Acinetobacter baumannii strain XH386 plasmid pXH386, complete sequence
205	NZ_CP021343	Acinetobacter baumannii strain B11911 plasmid unnamed1 map unlocalized
206	NZ_CP021344	Acinetobacter baumannii strain B11911 plasmid pB11911, complete sequence
207	NZ_CP021346	Acinetobacter baumannii strain B11911 plasmid unnamed1 map unlocalized
208	NZ_CP021348	Acinetobacter baumannii strain B8300 plasmid pB8300, complete sequence
209	NZ_CP021429	Acinetobacter pittii strain HUMV-6483 plasmid p11, complete sequence
210	NZ_CP021783	Acinetobacter baumannii strain A85 plasmid pA85-1, complete sequence
211	NZ_CP021784	Acinetobacter baumannii strain A85 plasmid pA85-1a, complete sequence
212	NZ_CP021785	Acinetobacter baumannii strain A85 plasmid pA85-1b, complete sequence
213	NZ_CP021786	Acinetobacter baumannii strain A85 plasmid pA85-2, complete sequence
214	NZ_CP021787	Acinetobacter baumannii strain A85 plasmid pA85-3, complete sequence
215	NZ_CP022284	Acinetobacter baumannii strain 7804 plasmid pAba7804a, complete sequence
216	NZ_CP022285	Acinetobacter baumannii strain 7804 plasmid pAba7804b, complete sequence

217	NZ_CP022299	Acinetobacter johnsonii strain IC001 plasmid pIC001A, complete sequence
218	NZ_CP022300	Acinetobacter johnsonii strain IC001 plasmid pIC001B, complete sequence
219	NZ_CP022301	Acinetobacter johnsonii strain IC001 plasmid pIC001C, complete sequence
220	NZ_CP022302	Acinetobacter johnsonii strain IC001 plasmid pIC001D, complete sequence
221	NZ_CP023021	Acinetobacter baumannii strain 9201 plasmid pAba9201a, complete sequence
222	NZ_CP023023	Acinetobacter baumannii strain 10324 plasmid pAba10324a, complete sequence
223	NZ_CP023024	Acinetobacter baumannii strain 10324 plasmid pAba10324b, complete sequence
224	NZ_CP023025	Acinetobacter baumannii strain 10324 plasmid pAba10324c, complete sequence
225	NZ_CP023027	Acinetobacter baumannii strain 10042 plasmid pAba10042a, complete sequence
226	NZ_CP023028	Acinetobacter baumannii strain 10042 plasmid pAba10042b, complete sequence
227	NZ_CP023030	Acinetobacter baumannii strain 9102 plasmid pAba9102a, complete sequence
228	NZ_CP023032	Acinetobacter baumannii strain 7847 plasmid pAba7847a, complete sequence
229	NZ_CP023033	Acinetobacter baumannii strain 7847 plasmid pAba7847b, complete sequence
230	NZ_CP023035	Acinetobacter baumannii strain 5845 plasmid pAba5845a, complete sequence
231	NZ_CP023300	Acinetobacter baumannii strain 11510 plasmid pAba11510b
232	NZ_CP024012	Acinetobacter sp. LoGeW2-3 plasmid unnamed1, complete sequence
233	NZ_CP024125	Acinetobacter baumannii strain AYP-A2 plasmid pAYP-A2, complete sequence
234	NZ_CP024419	Acinetobacter baumannii strain A388 plasmid pA388, complete sequence
235	NZ_CP024577	Acinetobacter baumannii strain AbPK1 plasmid pAbPK1a, complete sequence
236	NZ_CP024578	Acinetobacter baumannii strain AbPK1 plasmid pAbPK1b, complete sequence
237	NZ_CP025267	Acinetobacter baumannii isolate SMC_Paed_Ab_BL01 plasmid pSMC_AB_BL01_1, complete sequence
238	NZ_CP025619	Acinetobacter schindleri strain SGAir0122 plasmid pSGAir0122, complete sequence
239	NZ_CP026086	Acinetobacter pittii strain WCHAP005069 plasmid pOXA58_005069, complete sequence
240	NZ_CP026087	Acinetobacter pittii strain WCHAP005069 plasmid p1_005069, complete sequence
241	NZ_CP026088	Acinetobacter pittii strain WCHAP005069 plasmid p2_005069, complete sequence
242	NZ_CP026126	Acinetobacter baumannii strain ABNIH28 plasmid pABA-6973, complete sequence
243	NZ_CP026127	Acinetobacter baumannii strain ABNIH28 plasmid pNDM-0285, complete sequence
244	NZ_CP026128	Acinetobacter baumannii strain ABNIH28 plasmid pABA-1fe1, complete sequence
245	NZ_CP026129	Acinetobacter baumannii strain ABNIH28 plasmid pABA-2f10, complete sequence
246	NZ_CP026339	Acinetobacter baumannii strain 810CP plasmid pAba810CPb, complete sequence
247	NZ_CP026340	Acinetobacter baumannii strain 810CP plasmid pAba810CPa, complete sequence
248	NZ_CP026413	Acinetobacter sp. ACNIH2 plasmid pACI-35f6, complete sequence

249	NZ_CP026414	Acinetobacter sp. ACNIH2 plasmid pACI-235c, complete sequence
250	NZ_CP026415	Acinetobacter sp. ACNIH2 plasmid pACI-55cf, complete sequence
251	NZ_CP026416	Acinetobacter sp. ACNIH2 plasmid pACI-3569, complete sequence
252	NZ_CP026417	Acinetobacter sp. ACNIH2 plasmid pACI-c6b4, complete sequence
253	NZ_CP026418	Acinetobacter sp. ACNIH2 plasmid pKPC-8dee, complete sequence
254	NZ_CP026419	Acinetobacter sp. ACNIH2 plasmid pACI-6db8, complete sequence
255	NZ_CP026421	Acinetobacter sp. ACNIH1 plasmid pACI-3bd5, complete sequence
256	NZ_CP026422	Acinetobacter sp. ACNIH1 plasmid pACI-a283, complete sequence
257	NZ_CP026423	Acinetobacter sp. ACNIH1 plasmid pACI-b25a, complete sequence
258	NZ_CP026424	Acinetobacter sp. ACNIH1 plasmid pACI-148e, complete sequence
259	NZ_CP026425	Acinetobacter sp. ACNIH1 plasmid pNDM-9c17, complete sequence
260	NZ_CP026426	Acinetobacter sp. ACNIH1 plasmid pACI-df08, complete sequence
261	NZ_CP026427	Acinetobacter sp. ACNIH1 plasmid unnamed, complete sequence
262	NZ_CP026617	Acinetobacter sp. SWBY1 plasmid pSWBY1, complete sequence
263	NZ_CP026705	Acinetobacter baumannii strain AR_0056 plasmid tig00000058_pilon, complete sequence
264	NZ_CP026706	Acinetobacter baumannii strain AR_0056 plasmid tig00000059_pilon, complete sequence
265	NZ_CP026708	Acinetobacter baumannii strain AR_0056 plasmid tig00000534_pilon, complete sequence
266	NZ_CP026712	Acinetobacter baumannii strain AR_0063 plasmid unitig_2_pilon, complete sequence
267	NZ_CP026748	Acinetobacter baumannii strain WCHAB005133 plasmid p1_005133, complete sequence
268	NZ_CP026749	Acinetobacter baumannii strain WCHAB005133 plasmid pOXA58_005133, complete sequence
269	NZ_CP026944	Acinetobacter baumannii strain S1, AB1A2 plasmid pAbS1_01
270	NZ_CP026945	Acinetobacter baumannii strain S1, AB1A2 plasmid pAbS1_02
271	NZ_CP026946	Acinetobacter baumannii strain S1, AB1A2 plasmid pAbS1_03
272	NZ_CP027121	Acinetobacter baumannii strain AR_0056 plasmid unnamed2, complete sequence
273	NZ_CP027122	Acinetobacter baumannii strain AR_0056 plasmid unnamed1, complete sequence
274	NZ_CP027124	Acinetobacter baumannii strain AR_0056 plasmid unnamed3, complete sequence
275	NZ_CP027179	Acinetobacter baumannii strain AR_0070 plasmid unnamed1, complete sequence
276	NZ_CP027180	Acinetobacter baumannii strain AR_0070 plasmid unnamed2, complete sequence
277	NZ_CP027181	Acinetobacter baumannii strain AR_0070 plasmid unnamed3, complete sequence
278	NZ_CP027182	Acinetobacter baumannii strain AR_0070 plasmid unnamed4, complete sequence

279	NZ_CP027184	Acinetobacter baumannii strain AR_0052 plasmid unnamed2, complete sequence
280	NZ_CP027185	Acinetobacter baumannii strain AR_0052 plasmid unnamed1, complete sequence
281	NZ_CP027186	Acinetobacter baumannii strain AR_0052 plasmid unnamed4
282	NZ_CP027187	Acinetobacter baumannii strain AR_0052 plasmid unnamed3, complete sequence
283	NZ_CP027243	Acinetobacter baumannii strain WCHAB005078 plasmid p1_005078, complete sequence
284	NZ_CP027244	Acinetobacter baumannii strain WCHAB005078 plasmid p2_005078, complete sequence
285	NZ_CP027245	Acinetobacter baumannii strain WCHAB005078 plasmid pOXA58_005078, complete sequence
286	NZ_CP027247	Acinetobacter pittii strain WCHAP100004 plasmid p1_100004, complete sequence
287	NZ_CP027248	Acinetobacter pittii strain WCHAP100004 plasmid p2_100004, complete sequence
288	NZ_CP027249	Acinetobacter pittii strain WCHAP100004 plasmid pOXA58_100004, complete sequence
289	NZ_CP027251	Acinetobacter pittii strain WCHAP100020 plasmid p1_100020, complete sequence
290	NZ_CP027252	Acinetobacter pittii strain WCHAP100020 plasmid p2_100020, complete sequence
291	NZ_CP027253	Acinetobacter pittii strain WCHAP100020 plasmid pOXA58_100020, complete sequence
292	NZ_CP027483	Acinetobacter baumannii strain I43 plasmid pABI43, complete sequence
293	NZ_CP027529	Acinetobacter baumannii strain AR_0083 plasmid unnamed, complete sequence
294	NZ_CP027531	Acinetobacter baumannii strain AR_0088 plasmid unnamed1, complete sequence
295	NZ_CP027532	Acinetobacter baumannii strain AR_0088 plasmid unnamed2, complete sequence
296	NZ_CP027608	Acinetobacter baumannii strain AR_0102 plasmid unnamed1, complete sequence
297	NZ_CP027609	Acinetobacter baumannii strain AR_0102 plasmid unnamed2, complete sequence
298	NZ_CP027610	Acinetobacter baumannii strain AR_0101 plasmid unnamed, complete sequence
299	NZ_CP028139	Acinetobacter baumannii strain NCIMB 8209 plasmid pAbNCIMB8209_134, complete sequence
300	NZ_CP028556	Acinetobacter sp. WCHA45 plasmid p1_010045, complete sequence
301	NZ_CP028557	Acinetobacter sp. WCHA45 plasmid p2_010045, complete sequence
302	NZ_CP028558	Acinetobacter sp. WCHA45 plasmid p3_010045, complete sequence
303	NZ_CP028559	Acinetobacter sp. WCHA45 plasmid p4_010045, complete sequence
304	NZ_CP028560	Acinetobacter sp. WCHA45 plasmid pNDM1_010045, complete sequence
305	NZ_CP028569	Acinetobacter pittii strain WCHAP005046 plasmid p1_005046, complete sequence
306	NZ_CP028570	Acinetobacter pittii strain WCHAP005046 plasmid p2_005046, complete sequence
307	NZ_CP028571	Acinetobacter pittii strain WCHAP005046 plasmid p3_005046, complete sequence
308	NZ_CP028572	Acinetobacter pittii strain WCHAP005046 plasmid p4_005046, complete sequence

309	NZ_CP028573	Acinetobacter pittii strain WCHAP005046 plasmid pOXA58_005046, complete sequence
310	NZ_CP028798	Acinetobacter junii strain WCHAJ59 plasmid p1_010059, complete sequence
311	NZ_CP028799	Acinetobacter junii strain WCHAJ59 plasmid p2_010059, complete sequence
312	NZ_CP029389	Acinetobacter defluvii strain WCHA30 plasmid p1_010030, complete sequence
313	NZ_CP029390	Acinetobacter defluvii strain WCHA30 plasmid p2_010030, complete sequence
314	NZ_CP029391	Acinetobacter defluvii strain WCHA30 plasmid p3_010030, complete sequence
315	NZ_CP029392	Acinetobacter defluvii strain WCHA30 plasmid p4_010030, complete sequence
316	NZ_CP029393	Acinetobacter defluvii strain WCHA30 plasmid p5_010030, complete sequence
317	NZ_CP029394	Acinetobacter defluvii strain WCHA30 plasmid p6_010030, complete sequence
318	NZ_CP029395	Acinetobacter defluvii strain WCHA30 plasmid p7_010030, complete sequence
319	NZ_CP029396	Acinetobacter defluvii strain WCHA30 plasmid pOXA58_010030, complete sequence
320	NZ_CP029570	Acinetobacter baumannii strain DA33098 plasmid pDA33098-108, complete sequence
321	NZ_CP029571	Acinetobacter baumannii strain DA33098 plasmid pDA33098-71, complete sequence
322	NZ_CP029572	Acinetobacter baumannii strain DA33098 plasmid pDA33098-9-2, complete sequence
323	NZ_CP029573	Acinetobacter baumannii strain DA33098 plasmid pDA33098-9, complete sequence
324	NZ_CP029611	Acinetobacter pittii strain ST220 plasmid unnamed, complete sequence
325	NZ_CP030084	Acinetobacter baumannii strain Aba plasmid unnamed1, complete sequence
326	NZ_CP030107	Acinetobacter baumannii strain DA33382 plasmid pDA33382-2-2, complete sequence
327	NZ_CP030108	Acinetobacter baumannii strain DA33382 plasmid pDA33382-2, complete sequence
328	NZ_CP030109	Acinetobacter baumannii strain DA33382 plasmid pDA33382-85, complete sequence
329	NZ_CP030755	Acinetobacter schindleri strain H3 plasmid unnamed1, complete sequence
330	NZ_CP030756	Acinetobacter schindleri strain H3 plasmid unnamed2, complete sequence
331	NZ_CP030757	Acinetobacter schindleri strain H3 plasmid unnamed3, complete sequence
332	NZ_CP030758	Acinetobacter schindleri strain H3 plasmid unnamed4, complete sequence
333	NZ_CP031381	Acinetobacter baumannii ACICU plasmid pACICU1, complete sequence
334	NZ_CP031382	Acinetobacter baumannii ACICU plasmid pACICU2, complete sequence
335	NZ_CP031445	Acinetobacter baumannii strain MDR-UNC plasmid unnamed1, complete sequence
336	NZ_CP031446	Acinetobacter baumannii strain MDR-UNC plasmid pAB120, complete sequence
337	NZ_CP031709	Acinetobacter wuhouensis strain WCHA60 plasmid p1_010060, complete sequence
338	NZ_CP031710	Acinetobacter wuhouensis strain WCHA60 plasmid p2_010060, complete sequence
339	NZ_CP031711	Acinetobacter wuhouensis strain WCHA60 plasmid p3_010060, complete sequence
340	NZ_CP031712	Acinetobacter wuhouensis strain WCHA60 plasmid p4_010060, complete sequence

341	NZ_CP031713	Acinetobacter wuhouensis strain WCHA60 plasmid p5_010060, complete sequence
342	NZ_CP031714	Acinetobacter wuhouensis strain WCHA60 plasmid p6_010060, complete sequence
343	NZ_CP031715	Acinetobacter wuhouensis strain WCHA60 plasmid p7_010060, complete sequence
344	NZ_CP031973	Acinetobacter haemolyticus strain AN59 plasmid pAhaemAN59c, complete sequence
345	NZ_CP031974	Acinetobacter haemolyticus strain AN59 plasmid pAhaemAN59b, complete sequence
346	NZ_CP031975	Acinetobacter haemolyticus strain AN59 plasmid pAhaemAN59a, complete sequence
347	NZ_CP031977	Acinetobacter haemolyticus strain AN43 plasmid pAhaemAN43b, complete sequence
348	NZ_CP031978	Acinetobacter haemolyticus strain AN43 plasmid pAhaemAN43a
349	NZ_CP031980	Acinetobacter haemolyticus strain AN4 plasmid pAhaemAN4d, complete sequence
350	NZ_CP031981	Acinetobacter haemolyticus strain AN4 plasmid pAhaemAN4c, complete sequence
351	NZ_CP031982	Acinetobacter haemolyticus strain AN4 plasmid pAhaemAN4b, complete sequence
352	NZ_CP031983	Acinetobacter haemolyticus strain AN4 plasmid pAhaemAN4a, complete sequence
353	NZ_CP031985	Acinetobacter haemolyticus strain AN3 plasmid pAhaemAN3c, complete sequence
354	NZ_CP031986	Acinetobacter haemolyticus strain AN3 plasmid pAhaemAN3b, complete sequence
355	NZ_CP031987	Acinetobacter haemolyticus strain AN3 plasmid pAhaemAN3a, complete sequence
356	NZ_CP031989	Acinetobacter haemolyticus strain 5227 plasmid pAhaem5227b, complete sequence
357	NZ_CP031990	Acinetobacter haemolyticus strain 5227 plasmid pAhaem5227a, complete sequence
358	NZ_CP031992	Acinetobacter haemolyticus strain 2126ch plasmid pAhaem2126chf, complete sequence
359	NZ_CP031993	Acinetobacter haemolyticus strain 2126ch plasmid pAhaem2126che, complete sequence
360	NZ_CP031994	Acinetobacter haemolyticus strain 2126ch plasmid pAhaem2126chd, complete sequence
361	NZ_CP031995	Acinetobacter haemolyticus strain 2126ch plasmid pAhaem2126chc, complete sequence
362	NZ_CP031996	Acinetobacter haemolyticus strain 2126ch plasmid pAhaem2126chb, complete sequence
363	NZ_CP031997	Acinetobacter haemolyticus strain 2126ch plasmid pAhaem2126cha, complete sequence
364	NZ_CP031999	Acinetobacter haemolyticus strain INNSZ174 plasmid pAhaemINNSZ174c, complete sequence
365	NZ_CP032000	Acinetobacter haemolyticus strain INNSZ174 plasmid pAhaemINNSZ174b, complete sequence
366	NZ_CP032001	Acinetobacter haemolyticus strain INNSZ174 plasmid pAhaemINNSZ174a, complete sequence
367	NZ_CP032003	Acinetobacter haemolyticus strain 11616 plasmid pAhaem11616f, complete sequence
368	NZ_CP032004	Acinetobacter haemolyticus strain 11616 plasmid pAhaem11616e, complete sequence
369	NZ_CP032005	Acinetobacter haemolyticus strain 11616 plasmid pAhaem11616c, complete sequence
370	NZ_CP032006	Acinetobacter haemolyticus strain 11616 plasmid pAhaem11616d
371	NZ_CP032007	Acinetobacter haemolyticus strain 11616 plasmid pAhaem11616b, complete sequence

372	NZ_CP032008	Acinetobacter haemolyticus strain 11616 plasmid pAhaem11616a, complete sequence
373	NZ_CP032102	Acinetobacter lwoffii strain EK30A plasmid pALWEK1.1, complete sequence
374	NZ_CP032103	Acinetobacter lwoffii strain EK30A plasmid pALWEK1.10, complete sequence
375	NZ_CP032104	Acinetobacter lwoffii strain EK30A plasmid pALWEK1.11, complete sequence
376	NZ_CP032105	Acinetobacter lwoffii strain EK30A plasmid pALWEK1.2, complete sequence
377	NZ_CP032106	Acinetobacter lwoffii strain EK30A plasmid pALWEK1.3, complete sequence
378	NZ_CP032107	Acinetobacter lwoffii strain EK30A plasmid pALWEK1.4, complete sequence
379	NZ_CP032108	Acinetobacter lwoffii strain EK30A plasmid pALWEK1.6, complete sequence
380	NZ_CP032109	Acinetobacter lwoffii strain EK30A plasmid pALWEK1.7, complete sequence
381	NZ_CP032110	Acinetobacter lwoffii strain EK30A plasmid pALWEK1.8, complete sequence
382	NZ_CP032111	Acinetobacter lwoffii strain EK30A plasmid pALWEK1.9, complete sequence
383	NZ_CP032112	Acinetobacter lwoffii strain ED23-35 plasmid pALWED1.2, complete sequence
384	NZ_CP032113	Acinetobacter lwoffii strain ED23-35 plasmid pALWED1.4, complete sequence
385	NZ_CP032114	Acinetobacter lwoffii strain ED23-35 plasmid pALWED1.5, complete sequence
386	NZ_CP032115	Acinetobacter lwoffii strain ED23-35 plasmid pALWED1.6, complete sequence
387	NZ_CP032116	Acinetobacter lwoffii strain ED23-35 plasmid pALWED1.7, complete sequence
388	NZ_CP032117	Acinetobacter lwoffii strain ED45-23 plasmid pALWED2.2, complete sequence
389	NZ_CP032118	Acinetobacter lwoffii strain ED45-23 plasmid pALWED2.3, complete sequence
390	NZ_CP032119	Acinetobacter lwoffii strain ED45-23 plasmid pALWED2.4, complete sequence
391	NZ_CP032120	Acinetobacter lwoffii strain ED45-23 plasmid pALWED2.5, complete sequence
392	NZ_CP032121	Acinetobacter lwoffii strain ED45-23 plasmid pALWED2.6, complete sequence
393	NZ_CP032122	Acinetobacter lwoffii strain ED45-23 plasmid pALWED2.7, complete sequence
394	NZ_CP032123	Acinetobacter lwoffii strain ED45-23 plasmid pALWED2.8, complete sequence
395	NZ_CP032124	Acinetobacter lwoffii strain ED45-23 plasmid pALWED2.9, complete sequence
396	NZ_CP032127	Acinetobacter chinensis strain WCHAc010005 plasmid p1_010005, complete sequence
397	NZ_CP032128	Acinetobacter chinensis strain WCHAc010005 plasmid p2_010005, complete sequence
398	NZ_CP032129	Acinetobacter chinensis strain WCHAc010005 plasmid p3_010005, complete sequence
399	NZ_CP032130	Acinetobacter chinensis strain WCHAc010005 plasmid p4_010005, complete sequence
400	NZ_CP032131	Acinetobacter chinensis strain WCHAc010005 plasmid p5_010005, complete sequence
401	NZ_CP032132	Acinetobacter chinensis strain WCHAc010005 plasmid pNDM1_010005, complete sequence
402	NZ_CP032133	Acinetobacter chinensis strain WCHAc010005 plasmid pOXA58_010005, complete sequence
403	NZ_CP032138	Acinetobacter sp. WCHAc010052 plasmid p1_010052, complete sequence

404	NZ_CP032139	Acinetobacter sp. WCHAc010052 plasmid p2_010052, complete sequence
405	NZ_CP032140	Acinetobacter sp. WCHAc010052 plasmid p3_010052, complete sequence
406	NZ_CP032141	Acinetobacter sp. WCHAc010052 plasmid p4_010052, complete sequence
407	NZ_CP032142	Acinetobacter sp. WCHAc010052 plasmid pNDM1_010052, complete sequence
408	NZ_CP032216	Acinetobacter baumannii strain UPAB1 plasmid pAB5 map unlocalized
409	NZ_CP032217	Acinetobacter baumannii strain UPAB1 plasmid unnamed1
410	NZ_CP032218	Acinetobacter baumannii strain UPAB1 plasmid pAB5 map unlocalized
411	NZ_CP032219	Acinetobacter baumannii strain UPAB1 plasmid pAB5 map unlocalized
412	NZ_CP032220	Acinetobacter baumannii strain UPAB1 plasmid unnamed2
413	NZ_CP032267	Acinetobacter sp. WCHAc010034 plasmid p10_010034, complete sequence
414	NZ_CP032268	Acinetobacter sp. WCHAc010034 plasmid p1_010034, complete sequence
415	NZ_CP032269	Acinetobacter sp. WCHAc010034 plasmid p11_010034, complete sequence
416	NZ_CP032270	Acinetobacter sp. WCHAc010034 plasmid p2_010034, complete sequence
417	NZ_CP032271	Acinetobacter sp. WCHAc010034 plasmid p3_010034, complete sequence
418	NZ_CP032272	Acinetobacter sp. WCHAc010034 plasmid p4_010034, complete sequence
419	NZ_CP032273	Acinetobacter sp. WCHAc010034 plasmid p5_010034, complete sequence
420	NZ_CP032274	Acinetobacter sp. WCHAc010034 plasmid p6_010034, complete sequence
421	NZ_CP032275	Acinetobacter sp. WCHAc010034 plasmid p7_010034, complete sequence
422	NZ_CP032276	Acinetobacter sp. WCHAc010034 plasmid p8_010034, complete sequence
423	NZ_CP032277	Acinetobacter sp. WCHAc010034 plasmid p9_010034, complete sequence
424	NZ_CP032278	Acinetobacter sp. WCHAc010034 plasmid pNDM1_010034, complete sequence
425	NZ_CP032280	Acinetobacter sp. WCHA55 plasmid p1_010055, complete sequence
426	NZ_CP032281	Acinetobacter sp. WCHA55 plasmid p2_010055, complete sequence
427	NZ_CP032282	Acinetobacter sp. WCHA55 plasmid p3_010055, complete sequence
428	NZ_CP032283	Acinetobacter sp. WCHA55 plasmid p4_010055, complete sequence
429	NZ_CP032284	Acinetobacter sp. WCHA55 plasmid pNDM1_010055, complete sequence
430	NZ_CP032285	Acinetobacter sp. WCHA55 plasmid pOXA58_010055, complete sequence
431	NZ_CP032287	Acinetobacter lwoffii strain ED9-5a plasmid pALWED3.2, complete sequence
432	NZ_CP032288	Acinetobacter lwoffii strain ED9-5a plasmid pALWED3.3, complete sequence
433	NZ_CP032289	Acinetobacter lwoffii strain ED9-5a plasmid pALWED3.4, complete sequence
434	NZ_CP032290	Acinetobacter lwoffii strain ED9-5a plasmid pALWED3.6, complete sequence
435	NZ_CP032741	Acinetobacter baumannii strain C25 plasmid unnamed1

436	NZ_CP032742	Acinetobacter baumannii strain C25 plasmid unnamed2
437	NZ_CP033118	Acinetobacter wuhouensis strain WCHAW010062 plasmid p10_010062, complete sequence
438	NZ_CP033119	Acinetobacter wuhouensis strain WCHAW010062 plasmid p1_010062, complete sequence
439	NZ_CP033120	Acinetobacter wuhouensis strain WCHAW010062 plasmid p11_010062, complete sequence
440	NZ_CP033121	Acinetobacter wuhouensis strain WCHAW010062 plasmid p12_010062, complete sequence
441	NZ_CP033122	Acinetobacter wuhouensis strain WCHAW010062 plasmid p2_010062, complete sequence
442	NZ_CP033123	Acinetobacter wuhouensis strain WCHAW010062 plasmid p3_010062, complete sequence
443	NZ_CP033124	Acinetobacter wuhouensis strain WCHAW010062 plasmid p4_010062, complete sequence
444	NZ_CP033125	Acinetobacter wuhouensis strain WCHAW010062 plasmid p5_010062, complete sequence
445	NZ_CP033126	Acinetobacter wuhouensis strain WCHAW010062 plasmid p6_010062, complete sequence
446	NZ_CP033127	Acinetobacter wuhouensis strain WCHAW010062 plasmid p7_010062, complete sequence
447	NZ_CP033128	Acinetobacter wuhouensis strain WCHAW010062 plasmid p8_010062, complete sequence
448	NZ_CP033129	Acinetobacter wuhouensis strain WCHAW010062 plasmid p9_010062, complete sequence
449	NZ_CP033130	Acinetobacter wuhouensis strain WCHAW010062 plasmid pOXA23_010062, complete sequence
450	NZ_CP033131	Acinetobacter wuhouensis strain WCHAW010062 plasmid pOXA58_010062, complete sequence
451	NZ_CP033132	Acinetobacter wuhouensis strain WCHAW010062 plasmid pOXA653_010062, complete sequence
452	NZ_CP033244	Acinetobacter baumannii strain 7835 plasmid pAba7835a, complete sequence
453	NZ_CP033245	Acinetobacter baumannii strain 7835 plasmid pAba7835b, complete sequence
454	NZ_CP033751	Acinetobacter baumannii strain FDAARGOS_540 plasmid unnamed3
455	NZ_CP033752	Acinetobacter baumannii strain FDAARGOS_540 plasmid unnamed2, complete sequence
456	NZ_CP033753	Acinetobacter baumannii strain FDAARGOS_540 plasmid unnamed1, complete sequence
457	NZ_CP033769	Acinetobacter baumannii strain FDAARGOS_533 plasmid unnamed1, complete sequence
458	NZ_CP033770	Acinetobacter baumannii strain FDAARGOS_533 plasmid unnamed2, complete sequence

459	NZ_CP033857	Acinetobacter sp. FDAARGOS_493 plasmid unnamed1, complete sequence
460	NZ_CP033859	Acinetobacter sp. FDAARGOS_493 plasmid unnamed2
461	NZ_CP033860	Acinetobacter sp. FDAARGOS_493 plasmid unnamed3, complete sequence
462	NZ_CP033863	Acinetobacter sp. FDAARGOS_560 plasmid unnamed1
463	NZ_CP033864	Acinetobacter sp. FDAARGOS_560 plasmid unnamed2
464	NZ_CP033867	Acinetobacter sp. FDAARGOS_494 plasmid unnamed
465	NZ_CP033870	Acinetobacter baumannii strain MRSN15313 plasmid p597A-6.7, complete sequence
466	NZ_CP033871	Acinetobacter baumannii strain MRSN15313 plasmid p597A-14.8, complete sequence
467	NZ_CP033872	Acinetobacter baumannii strain MRSN15313 plasmid pAb-MCR4.3, complete sequence
468	NZ_CP034093	Acinetobacter baumannii strain A52 plasmid pA52-1, complete sequence
469	NZ_CP034094	Acinetobacter baumannii strain A52 plasmid pA52-2, complete sequence
470	NZ_CP034095	Acinetobacter baumannii strain A52 plasmid pA52-3, complete sequence
471	NZ_CP034096	Acinetobacter baumannii strain A52 plasmid pA52-4, complete sequence
472	NZ_CP034097	Acinetobacter baumannii strain A52 plasmid pA52-OXA-72, complete sequence
473	NZ_CP035044	Acinetobacter baumannii strain ABUH796 plasmid p13.0Kbp, complete sequence
474	NZ_CP035046	Acinetobacter baumannii strain ABUH793 plasmid p107.0Kbp, complete sequence
475	NZ_CP035047	Acinetobacter baumannii strain ABUH793 plasmid p74.1Kbp, complete sequence
476	NZ_CP035048	Acinetobacter baumannii strain ABUH793 plasmid p10.9Kbp, complete sequence
477	NZ_CP035050	Acinetobacter baumannii strain ABUH773 plasmid p11.8Kbp, complete sequence
478	NZ_CP035052	Acinetobacter baumannii strain ABUH763 plasmid p74.1Kbp, complete sequence
479	NZ_CP035053	Acinetobacter baumannii strain ABUH763 plasmid p11.0Kbp, complete sequence
480	NZ_CP035935	Acinetobacter cumulans strain WCHAc060092 plasmid pNDM1_060092, complete sequence
481	NZ_CP035936	Acinetobacter cumulans strain WCHAc060092 plasmid p1_060092, complete sequence
482	NZ_CP035937	Acinetobacter cumulans strain WCHAc060092 plasmid p2_060092, complete sequence
483	NZ_CP035938	Acinetobacter cumulans strain WCHAc060092 plasmid p3_060092, complete sequence
484	NZ_CP035939	Acinetobacter cumulans strain WCHAc060092 plasmid p4_060092, complete sequence
485	NZ_CP035940	Acinetobacter cumulans strain WCHAc060092 plasmid p5_060092, complete sequence
486	NZ_CP035941	Acinetobacter cumulans strain WCHAc060092 plasmid p6_060092, complete sequence
487	NZ_CP035942	Acinetobacter cumulans strain WCHAc060092 plasmid p7_060092, complete sequence
488	NZ_CP035943	Acinetobacter cumulans strain WCHAc060092 plasmid p8_060092, complete sequence
489	NZ_CP036284	Acinetobacter baumannii strain TG60155 plasmid p60155_1, complete sequence
490	NZ_CP036285	Acinetobacter baumannii strain TG60155 plasmid p60155_2, complete sequence

491	NZ_CP036286	Acinetobacter baumannii strain TG60155 plasmid p60155_3, complete sequence
492	NZ_CP037425	Acinetobacter johnsonii strain M19 plasmid pFM-M19, complete sequence
493	NZ_CP038010	Acinetobacter haemolyticus strain TJR01 plasmid pAHTJR1, complete sequence
494	NZ_CP038011	Acinetobacter haemolyticus strain TJR01 plasmid pAHTJR2, complete sequence
495	NZ_CP038023	Acinetobacter radioresistens strain DD78 plasmid pAR1, complete sequence
496	NZ_CP038024	Acinetobacter radioresistens strain DD78 plasmid pAR2, complete sequence
497	NZ_CP038025	Acinetobacter radioresistens strain DD78 plasmid pAR3, complete sequence
498	NZ_CP038259	Acinetobacter baumannii strain 39741 plasmid pEH_gr13, complete sequence
499	NZ_CP038260	Acinetobacter baumannii strain 39741 plasmid pEH_gr3, complete sequence
500	NZ_CP038261	Acinetobacter baumannii strain 39741 plasmid pEH_mcr4.3, complete sequence
501	NZ_CP038263	Acinetobacter baumannii strain LEV1449/17Ec plasmid pEC_gr13, complete sequence
502	NZ_CP038264	Acinetobacter baumannii strain LEV1449/17Ec plasmid pEC_gr6, complete sequence
503	NZ_CP038265	Acinetobacter baumannii strain LEV1449/17Ec plasmid pEC_mcr4.3, complete sequence
504	NZ_CP038501	Acinetobacter baumannii strain CIAT758 plasmid unnamed1, complete sequence
505	NZ_CP038502	Acinetobacter baumannii strain CIAT758 plasmid unnamed2, complete sequence
506	NZ_CP038503	Acinetobacter baumannii strain CIAT758 plasmid unnamed3, complete sequence
507	NZ_CP038645	Acinetobacter baumannii strain ACN21 plasmid unnamed1, complete sequence
508	NZ_CP038646	Acinetobacter baumannii strain ACN21 plasmid unnamed2, complete sequence
509	NZ_CP038647	Acinetobacter baumannii strain ACN21 plasmid unnamed3, complete sequence
510	NZ_CP038648	Acinetobacter baumannii strain ACN21 plasmid unnamed4, complete sequence
511	NZ_CP038649	Acinetobacter baumannii strain ACN21 plasmid unnamed5, complete sequence
512	NZ_CP038650	Acinetobacter baumannii strain ACN21 plasmid unnamed6, complete sequence
513	NZ_CP038651	Acinetobacter baumannii strain ACN21 plasmid unnamed7, complete sequence
514	NZ_CP038652	Acinetobacter baumannii strain ACN21 plasmid unnamed8, complete sequence
515	NZ_CP039024	Acinetobacter baumannii ATCC 17978 substr. PMR-High plasmid unnamed1
516	NZ_CP039026	Acinetobacter baumannii ATCC 17978 substr. PMR-High plasmid unnamed2
517	NZ_CP039027	Acinetobacter baumannii ATCC 17978 substr. Lab-WT plasmid unnamed1
518	NZ_CP039029	Acinetobacter baumannii ATCC 17978 substr. Lab-WT plasmid unnamed2
519	NZ_CP039030	Acinetobacter baumannii ATCC 17978 substr. Lab-WT plasmid unnamed3
520	NZ_CP039144	Acinetobacter sp. 10FS3-1 plasmid p10FS3-1-1, complete sequence
521	NZ_CP039145	Acinetobacter sp. 10FS3-1 plasmid p10FS3-1-2, complete sequence
522	NZ_CP039146	Acinetobacter sp. 10FS3-1 plasmid p10FS3-1-3, complete sequence

523	NZ_CP039147	Acinetobacter sp. 10FS3-1 plasmid p10FS3-1-4, complete sequence
524	NZ_CP039148	Acinetobacter sp. 10FS3-1 plasmid p10FS3-1-5, complete sequence
525	NZ_CP039149	Acinetobacter sp. 10FS3-1 plasmid p10FS3-1-6, complete sequence
526	NZ_CP039150	Acinetobacter sp. 10FS3-1 plasmid p10FS3-1-7, complete sequence
527	NZ_CP039151	Acinetobacter sp. 10FS3-1 plasmid p10FS3-1-8, complete sequence
528	NZ_CP039152	Acinetobacter sp. 10FS3-1 plasmid p10FS3-1-9, complete sequence
529	NZ_CP039153	Acinetobacter sp. 10FS3-1 plasmid p10FS3-1-10, complete sequence
530	NZ_CP039154	Acinetobacter sp. 10FS3-1 plasmid p10FS3-1-11, complete sequence
531	NZ_CP039155	Acinetobacter sp. 10FS3-1 plasmid p10FS3-1-12, complete sequence
532	NZ_CP039342	Acinetobacter baumannii strain TG31986 plasmid pTG31986, complete sequence
533	NZ_CP039344	Acinetobacter baumannii strain TG31302 plasmid pTG31302, complete sequence
534	NZ_CP039519	Acinetobacter baumannii strain TG22653 plasmid pTG22653, complete sequence
535	NZ_CP039521	Acinetobacter baumannii strain TG22627 plasmid pTG22627, complete sequence
536	NZ_CP039931	Acinetobacter baumannii strain TG29392 plasmid pTG29392_1, complete sequence
537	NZ_CP039932	Acinetobacter baumannii strain TG29392 plasmid pTG29392_2, complete sequence
538	NZ_CP039933	Acinetobacter baumannii strain TG29392 plasmid pTG29392_3, complete sequence
539	NZ_CP039994	Acinetobacter baumannii strain TG22182 plasmid pTG22182_1, complete sequence
540	NZ_CP039995	Acinetobacter baumannii strain TG22182 plasmid pTG22182_2, complete sequence
541	NZ_CP040048	Acinetobacter baumannii strain VB1190 plasmid unnamed1, complete sequence
542	NZ_CP040049	Acinetobacter baumannii strain VB1190 plasmid unnamed2, complete sequence
543	NZ_CP040051	Acinetobacter baumannii strain VB16141 plasmid unnamed1, complete sequence
544	NZ_CP040052	Acinetobacter baumannii strain VB16141 plasmid unnamed2, complete sequence
545	NZ_CP040081	Acinetobacter baumannii strain SP304 plasmid unnamed1, complete sequence
546	NZ_CP040082	Acinetobacter baumannii strain SP304 plasmid unnamed2, complete sequence
547	NZ_CP040083	Acinetobacter baumannii strain SP304 plasmid unnamed3, complete sequence
548	NZ_CP040085	Acinetobacter baumannii strain VB33071 plasmid unnamed1, complete sequence
549	NZ_CP040086	Acinetobacter baumannii strain VB33071 plasmid unnamed2, complete sequence
550	NZ_CP040088	Acinetobacter baumannii strain VB35575 plasmid unnamed1, complete sequence
551	NZ_CP040260	Acinetobacter baumannii strain P7774 plasmid unnamed1, complete sequence
552	NZ_CP040261	Acinetobacter baumannii strain P7774 plasmid unnamed2, complete sequence
553	NZ_CP040262	Acinetobacter baumannii strain P7774 plasmid unnamed3, complete sequence
554	NZ_CP040426	Acinetobacter baumannii strain PB364 plasmid pPB364_1, complete sequence

555	NZ_CP040427	Acinetobacter baumannii strain PB364 plasmid pPB364_2, complete sequence
556	NZ_CP040912	Acinetobacter pittii strain AB17H194 plasmid pAB17H194-1, complete sequence
557	NZ_CP040913	Acinetobacter pittii strain AB17H194 plasmid pAB17H194-2, complete sequence
558	NZ_CP041149	Acinetobacter baumannii strain CUVET-MIC596 plasmid pCUVET596, complete sequence
559	NZ_CP041225	Acinetobacter haemolyticus strain AN54 plasmid pAhaeAN54d
560	NZ_CP041226	Acinetobacter haemolyticus strain AN54 plasmid pAhaeAN54c
561	NZ_CP041227	Acinetobacter haemolyticus strain AN54 plasmid plUnnamed
562	NZ_CP041228	Acinetobacter haemolyticus strain AN54 plasmid pAhaemAN54a
563	NZ_CP041229	Acinetobacter haemolyticus strain AN54 plasmid pAhaeAN54e, complete sequence
564	NZ_CP041287	Acinetobacter indicus strain 94-2 plasmid p94-2-p4, complete sequence
565	NZ_CP041288	Acinetobacter indicus strain 94-2 plasmid p94-2-p2, complete sequence
566	NZ_CP041289	Acinetobacter indicus strain 94-2 plasmid p94-2-p5, complete sequence
567	NZ_CP041290	Acinetobacter indicus strain 94-2 plasmid p94-2-tetX3, complete sequence
568	NZ_CP041292	Acinetobacter indicus strain 94-2 plasmid p94-2-p3, complete sequence
569	NZ_CP041293	Acinetobacter indicus strain 80-1-2 plasmid p80-1-2-p5, complete sequence
570	NZ_CP041294	Acinetobacter indicus strain 80-1-2 plasmid p80-1-2-p3, complete sequence
571	NZ_CP041296	Acinetobacter indicus strain 80-1-2 plasmid p80-1-2-p2, complete sequence
572	NZ_CP041297	Acinetobacter indicus strain 80-1-2 plasmid p80-1-2-tetX3, complete sequence
573	NZ_CP041298	Acinetobacter indicus strain 80-1-2 plasmid p80-1-2-p4, complete sequence
574	NZ_CP041366	Acinetobacter tandoii strain SE63 plasmid p1, complete sequence
575	NZ_CP041367	Acinetobacter tandoii strain SE63 plasmid p2, complete sequence
576	NZ_CP041588	Acinetobacter baumannii strain J9 plasmid pJ9-1, complete sequence
577	NZ_CP041589	Acinetobacter baumannii strain J9 plasmid pJ9-2, complete sequence
578	NZ_CP041590	Acinetobacter baumannii strain J9 plasmid pJ9-3, complete sequence
579	NZ_CP042365	Acinetobacter pittii strain C54 plasmid pC54_001, complete sequence
580	NZ_CP042366	Acinetobacter pittii strain C54 plasmid pC54_002
581	NZ_CP042367	Acinetobacter pittii strain C54 plasmid pC54_003, complete sequence
582	NZ_CP042368	Acinetobacter pittii strain C54 plasmid pC54_004, complete sequence
583	NZ_CP042369	Acinetobacter pittii strain C54 plasmid pC54_005, complete sequence
584	NZ_CP042370	Acinetobacter pittii strain C54 plasmid pC54_006
585	NZ_CP042557	Acinetobacter baumannii strain E47 plasmid pE47_001, complete sequence
586	NZ_CP042558	Acinetobacter baumannii strain E47 plasmid pE47_002, complete sequence

587	NZ_CP042559	Acinetobacter baumannii strain E47 plasmid pE47_003, complete sequence
588	NZ_CP042560	Acinetobacter baumannii strain E47 plasmid pE47_004, complete sequence
589	NZ_CP042561	Acinetobacter baumannii strain E47 plasmid pE47_005, complete sequence
590	NZ_CP042562	Acinetobacter baumannii strain E47 plasmid pE47_006, complete sequence
591	NZ_CP042563	Acinetobacter baumannii strain E47 plasmid pE47_007, complete sequence
592	NZ_CP042564	Acinetobacter baumannii strain E47 plasmid pE47_008, complete sequence
593	NZ_CP042565	Acinetobacter baumannii strain E47 plasmid pE47_009, complete sequence
594	NZ_CP042842	Acinetobacter baumannii strain ATCC BAA-1790 plasmid unnamed, complete sequence
595	NZ_CP042995	Acinetobacter nosocomialis strain J1A plasmid unnamed1, complete sequence
596	NZ_CP042996	Acinetobacter nosocomialis strain J1A plasmid unnamed2, complete sequence
597	NZ_CP043053	Acinetobacter pittii strain AP43 plasmid pAP43-OXA58-NDM1, complete sequence
598	NZ_CP043054	Acinetobacter pittii strain AP43 plasmid pAP43-2, complete sequence
599	NZ_CP043055	Acinetobacter pittii strain AP43 plasmid pAP43-3, complete sequence
600	NZ_CP043308	Acinetobacter johnsonii strain Acsw19 plasmid pAcsw19-1, complete sequence
601	NZ_CP043309	Acinetobacter johnsonii strain Acsw19 plasmid pAcsw19-2, complete sequence
602	NZ_CP043310	Acinetobacter johnsonii strain Acsw19 plasmid pAcsw19-3, complete sequence
603	NZ_CP043954	Acinetobacter baumannii strain K09-14 plasmid pK09-14, complete sequence
604	NZ_CP044019	Acinetobacter indicus strain HY20 plasmid pAI01, complete sequence
605	NZ_CP044020	Acinetobacter indicus strain HY20 plasmid pAI02, complete sequence
606	NZ_CP044357	Acinetobacter baumannii strain CAM180-1 plasmid pCAM180A, complete sequence
607	NZ_CP044358	Acinetobacter baumannii strain CAM180-1 plasmid pCAM180B, complete sequence
608	NZ_CP044446	Acinetobacter indicus strain CMG3-2 plasmid pCMG3-2-1, complete sequence
609	NZ_CP044447	Acinetobacter indicus strain CMG3-2 plasmid pCMG3-2-2, complete sequence
610	NZ_CP044448	Acinetobacter indicus strain CMG3-2 plasmid pCMG3-2-3, complete sequence
611	NZ_CP044449	Acinetobacter indicus strain CMG3-2 plasmid pCMG3-2-4, complete sequence
612	NZ_CP044451	Acinetobacter indicus strain MMS9-2 plasmid pMMS9-2-1, complete sequence
613	NZ_CP044452	Acinetobacter indicus strain MMS9-2 plasmid pMMS9-2-2, complete sequence
614	NZ_CP044453	Acinetobacter indicus strain MMS9-2 plasmid pMMS9-2-3, complete sequence
615	NZ_CP044454	Acinetobacter indicus strain MMS9-2 plasmid pMMS9-2-4, complete sequence
616	NZ_CP044456	Acinetobacter indicus strain B18 plasmid pB18-1, complete sequence
617	NZ_CP044457	Acinetobacter indicus strain B18 plasmid pB18-2, complete sequence
618	NZ_CP044458	Acinetobacter indicus strain B18 plasmid pB18-3, complete sequence

619	NZ_CP044459	Acinetobacter indicus strain B18 plasmid pB18-4, complete sequence
620	NZ_CP044460	Acinetobacter indicus strain B18 plasmid pB18-5, complete sequence
621	NZ_CP044461	Acinetobacter indicus strain B18 plasmid pB18-6, complete sequence
622	NZ_CP044462	Acinetobacter indicus strain B18 plasmid pB18-7, complete sequence
623	NZ_CP044464	Acinetobacter schindleri strain HZE23-1 plasmid pHZE23-1-1, complete sequence
624	NZ_CP044465	Acinetobacter schindleri strain HZE23-1 plasmid pHZE23-1-2, complete sequence
625	NZ_CP044466	Acinetobacter schindleri strain HZE23-1 plasmid pHZE23-1-3, complete sequence
626	NZ_CP044467	Acinetobacter schindleri strain HZE23-1 plasmid pHZE23-1-4, complete sequence
627	NZ_CP044468	Acinetobacter schindleri strain HZE23-1 plasmid pHZE23-1-5, complete sequence
628	NZ_CP044469	Acinetobacter schindleri strain HZE23-1 plasmid pHZE23-1-6, complete sequence
629	NZ_CP044470	Acinetobacter schindleri strain HZE23-1 plasmid pHZE23-1-7, complete sequence
630	NZ_CP044471	Acinetobacter schindleri strain HZE23-1 plasmid pHZE23-1-8, complete sequence
631	NZ_CP044472	Acinetobacter schindleri strain HZE23-1 plasmid pHZE23-1-9, complete sequence
632	NZ_CP044473	Acinetobacter schindleri strain HZE23-1 plasmid pHZE23-1-10, complete sequence
633	NZ_CP044475	Acinetobacter schindleri strain HZE33-1 plasmid pHZE33-1-1, complete sequence
634	NZ_CP044476	Acinetobacter schindleri strain HZE33-1 plasmid pHZE33-1-2, complete sequence
635	NZ_CP044477	Acinetobacter schindleri strain HZE33-1 plasmid pHZE33-1-3, complete sequence
636	NZ_CP044478	Acinetobacter schindleri strain HZE33-1 plasmid pHZE33-1-4, complete sequence
637	NZ_CP044479	Acinetobacter schindleri strain HZE33-1 plasmid pHZE33-1-5, complete sequence
638	NZ_CP044480	Acinetobacter schindleri strain HZE33-1 plasmid pHZE33-1-6, complete sequence
639	NZ_CP044481	Acinetobacter schindleri strain HZE33-1 plasmid pHZE33-1-7, complete sequence
640	NZ_CP044482	Acinetobacter schindleri strain HZE33-1 plasmid pHZE33-1-8, complete sequence
641	NZ_CP044484	Acinetobacter schindleri strain HZE30-1 plasmid pHZE30-1-1, complete sequence
642	NZ_CP044485	Acinetobacter schindleri strain HZE30-1 plasmid pHZE30-1-2, complete sequence
643	NZ_CP044486	Acinetobacter schindleri strain HZE30-1 plasmid pHZE30-1-3, complete sequence
644	NZ_CP044487	Acinetobacter schindleri strain HZE30-1 plasmid pHZE30-1-4, complete sequence
645	NZ_CP044488	Acinetobacter schindleri strain HZE30-1 plasmid pHZE30-1-5, complete sequence
646	NZ_CP044489	Acinetobacter schindleri strain HZE30-1 plasmid pHZE30-1-6, complete sequence
647	NZ_CP044490	Acinetobacter schindleri strain HZE30-1 plasmid pHZE30-1-7, complete sequence
648	NZ_CP044491	Acinetobacter schindleri strain HZE30-1 plasmid pHZE30-1-8, complete sequence
649	NZ_CP044518	Acinetobacter baumannii strain 31FS3-2 plasmid p31FS3-2-1, complete sequence
650	NZ_CP044520	Acinetobacter baumannii strain 29FS20 plasmid p29FS20-1, complete sequence

651	NZ_CP044521	Acinetobacter baumannii strain 29FS20 plasmid p29FS20-2, complete sequence
652	NZ_CP045108	Acinetobacter baumannii strain ATCC 19606 plasmid p1ATCC19606, complete sequence
653	NZ_CP045109	Acinetobacter baumannii strain ATCC 19606 plasmid p2ATCC19606, complete sequence
654	NZ_CP045123	Acinetobacter indicus strain XG03 plasmid pXG-13k, complete sequence
655	NZ_CP045124	Acinetobacter indicus strain XG03 plasmid pXG-160kb, complete sequence
656	NZ_CP045125	Acinetobacter indicus strain XG03 plasmid pXG-4k, complete sequence
657	NZ_CP045126	Acinetobacter indicus strain XG03 plasmid pXG-5k, complete sequence
658	NZ_CP045128	Acinetobacter indicus strain XG03 plasmid pXG03-X3, complete sequence
659	NZ_CP045130	Acinetobacter indicus strain TQ04 plasmid p4TQ-NDM, complete sequence
660	NZ_CP045132	Acinetobacter indicus strain TQ18 plasmid p18TQ-X3, complete sequence
661	NZ_CP045133	Acinetobacter indicus strain TQ18 plasmid p18TQ-NDM, complete sequence
662	NZ_CP045134	Acinetobacter indicus strain TQ18 plasmid p18TQ-13k, complete sequence
663	NZ_CP045136	Acinetobacter indicus strain XG01 plasmid pXG01-X3, complete sequence
664	NZ_CP045196	Acinetobacter indicus strain TQ23 plasmid p23TQ-37k, complete sequence
665	NZ_CP045197	Acinetobacter indicus strain TQ23 plasmid p23TQ-NDM, complete sequence
666	NZ_CP045561	Acinetobacter nosocomialis strain AC1530 plasmid pAC1530, complete sequence
667	NZ_CP046043	Acinetobacter towneri strain 19110F47 plasmid p19110F47-1, complete sequence
668	NZ_CP046044	Acinetobacter towneri strain 19110F47 plasmid p19110F47-2, complete sequence
669	NZ_CP046295	Acinetobacter lwoffii strain FDAARGOS_552 plasmid unnamed1, complete sequence
670	NZ_CP046297	Acinetobacter lwoffii strain FDAARGOS_552 plasmid unnamed2, complete sequence
671	NZ_CP046298	Acinetobacter lwoffii strain FDAARGOS_552 plasmid unnamed3, complete sequence
672	NZ_CP046299	Acinetobacter lwoffii strain FDAARGOS_552 plasmid unnamed4, complete sequence
673	NZ_CP046537	Acinetobacter baumannii strain XL380 plasmid unnamed, complete sequence
674	NZ_CP046655	Acinetobacter baumannii strain ATCC 19606 plasmid pMAC, complete sequence
675	NZ_CP046899	Acinetobacter baumannii strain A1429 plasmid pA1429c, complete sequence
676	NZ_CP046900	Acinetobacter baumannii strain A1429 plasmid pA1429b, complete sequence
677	NZ_CP046901	Acinetobacter baumannii strain A1429 plasmid pA1429a, complete sequence
678	NZ_CP047974	Acinetobacter baumannii strain DETAB-P2 plasmid pDETAB1, complete sequence
679	NZ_CP047975	Acinetobacter baumannii strain DETAB-P2 plasmid pDETAB2, complete sequence
680	NZ_CP047976	Acinetobacter baumannii strain DETAB-P2 plasmid pDETAB3, complete sequence
681	NZ_CP048015	Acinetobacter towneri strain 205 plasmid pAT205, complete sequence
682	NZ_CP048016	Acinetobacter towneri strain 205 plasmid pAT205-1

683	NZ_CP048017	Acinetobacter towneri strain 205 plasmid pAT205-2
684	NZ_CP048018	Acinetobacter towneri strain 205 plasmid pAT205-3
685	NZ_CP048655	Acinetobacter indicus strain C15_T plasmid pC15-1, complete sequence
686	NZ_CP048656	Acinetobacter indicus strain C15_T plasmid pC15-2, complete sequence
687	NZ_CP048657	Acinetobacter indicus strain C15_T plasmid pC15-3, complete sequence
688	NZ_CP048658	Acinetobacter indicus strain C15_T plasmid pC15-4
689	NZ_CP048660	Acinetobacter piscicola strain YH12207_T plasmid pYH12207-1, complete sequence
690	NZ_CP048661	Acinetobacter piscicola strain YH12207_T plasmid pYH12207-2, complete sequence
691	NZ_CP048662	Acinetobacter piscicola strain YH12207_T plasmid pYH12207-3, complete sequence
692	NZ_CP048663	Acinetobacter piscicola strain YH12207_T plasmid pYH12207-4, complete sequence
693	NZ_CP048664	Acinetobacter piscicola strain YH12207_T plasmid pYH12207-5, complete sequence
694	NZ_CP048665	Acinetobacter piscicola strain YH12207_T plasmid pYH12207-6, complete sequence
695	NZ_CP048666	Acinetobacter piscicola strain YH12207_T plasmid pYH12207-7, complete sequence
696	NZ_CP048667	Acinetobacter piscicola strain YH12207_T plasmid pYH12207-8, complete sequence
697	NZ_CP048668	Acinetobacter piscicola strain YH12207_T plasmid pYH12207-9, complete sequence
698	NZ_CP048669	Acinetobacter piscicola strain YH12207_T plasmid pYH12207-10, complete sequence
699	NZ_CP048671	Acinetobacter sp. YH12138 strain YH12138_T plasmid pYH12138-1, complete sequence
700	NZ_CP048672	Acinetobacter sp. YH12138 strain YH12138_T plasmid pYH12138-2
701	NZ_CP048673	Acinetobacter sp. YH12138 strain YH12138_T plasmid pYH12138-3, complete sequence
702	NZ_CP048674	Acinetobacter sp. YH12138 strain YH12138_T plasmid pYH12138-4, complete sequence
703	NZ_CP048675	Acinetobacter sp. YH12138 strain YH12138_T plasmid pYH12138-5, complete sequence
704	NZ_CP048676	Acinetobacter sp. YH12138 strain YH12138_T plasmid pYH12138-6, complete sequence
705	NZ_CP048677	Acinetobacter sp. YH12138 strain YH12138_T plasmid pYH12138-7, complete sequence
706	NZ_CP048678	Acinetobacter sp. YH12138 strain YH12138_T plasmid pYH12138-8, complete sequence
707	NZ_CP048679	Acinetobacter sp. YH12138 strain YH12138_T plasmid pYH12138-9, complete sequence
708	NZ_CP048680	Acinetobacter sp. YH12138 strain YH12138_T plasmid pYH12138-10, complete sequence
709	NZ_CP048682	Acinetobacter indicus strain Q186-3_T plasmid pQ186-3-1, complete sequence
710	NZ_CP048683	Acinetobacter indicus strain Q186-3_T plasmid pQ186-3-2, complete sequence
711	NZ_CP048684	Acinetobacter indicus strain Q186-3_T plasmid pQ186-3-3, complete sequence
712	NZ_CP048828	Acinetobacter baumannii strain ABF9692 plasmid pABF9692, complete sequence
713	NZ_CP049364	Acinetobacter baumannii strain ATCC 17978 plasmid pAB1, complete sequence
714	NZ_CP049365	Acinetobacter baumannii strain ATCC 17978 plasmid pAB2, complete sequence

715	NZ_CP049807	Acinetobacter pittii strain A1254 plasmid pA1254_1, complete sequence
716	NZ_CP049808	Acinetobacter pittii strain A1254 plasmid pA1254_2, complete sequence
717	NZ_CP049809	Acinetobacter pittii strain A1254 plasmid pA1254_3, complete sequence
718	NZ_CP049810	Acinetobacter pittii strain A1254 plasmid pA1254_4, complete sequence
719	NZ_CP049917	Acinetobacter lanii strain 185 plasmid unnamed1
720	NZ_CP050386	Acinetobacter baumannii strain VB82 plasmid pVB82_1, complete sequence
721	NZ_CP050387	Acinetobacter baumannii strain VB82 plasmid pVB82_2, complete sequence
722	NZ_CP050389	Acinetobacter baumannii strain VB473 plasmid pVB473_1, complete sequence
723	NZ_CP050391	Acinetobacter baumannii strain VB723 plasmid pVB723_1, complete sequence
724	NZ_CP050392	Acinetobacter baumannii strain VB11737 plasmid pVB11737_1, complete sequence
725	NZ_CP050393	Acinetobacter baumannii strain VB11737 plasmid pVB11737_2, complete sequence
726	NZ_CP050394	Acinetobacter baumannii strain VB11737 plasmid pVB11737_3, complete sequence
727	NZ_CP050395	Acinetobacter baumannii strain VB11737 plasmid pVB11737_4, complete sequence
728	NZ_CP050396	Acinetobacter baumannii strain VB11737 plasmid pVB11737_5, complete sequence
729	NZ_CP050397	Acinetobacter baumannii strain VB11737 plasmid pVB11737_6, complete sequence
730	NZ_CP050398	Acinetobacter baumannii strain VB11737 plasmid pVB11737_7, complete sequence
731	NZ_CP050399	Acinetobacter baumannii strain VB11737 plasmid pVB11737_8, complete sequence
732	NZ_CP050402	Acinetobacter baumannii strain VB2181 plasmid pVB2181, complete sequence
733	NZ_CP050404	Acinetobacter baumannii strain VB2486 plasmid pVB2486_1, complete sequence
734	NZ_CP050405	Acinetobacter baumannii strain VB2486 plasmid pVB2486_2, complete sequence
735	NZ_CP050406	Acinetobacter baumannii strain VB2486 plasmid pVB2486_3, complete sequence
736	NZ_CP050407	Acinetobacter baumannii strain VB2486 plasmid pVB2486_4, complete sequence
737	NZ_CP050408	Acinetobacter baumannii strain VB2486 plasmid pVB2486_5, complete sequence
738	NZ_CP050409	Acinetobacter baumannii strain VB2486 plasmid pVB2486_6, complete sequence
739	NZ_CP050411	Acinetobacter baumannii strain PM1912235 plasmid pPM192235_1, complete sequence
740	NZ_CP050413	Acinetobacter baumannii strain PM192696 plasmid pPM192696_1, complete sequence
741	NZ_CP050414	Acinetobacter baumannii strain PM192696 plasmid pPM192696_2, complete sequence
742	NZ_CP050416	Acinetobacter baumannii strain PM193665 plasmid pPM193665_1, complete sequence
743	NZ_CP050417	Acinetobacter baumannii strain PM193665 plasmid pPM193665_2, complete sequence
744	NZ_CP050418	Acinetobacter baumannii strain PM193665 plasmid pPM193665_3, complete sequence
745	NZ_CP050419	Acinetobacter baumannii strain PM193665 plasmid pPM193665_4, complete sequence
746	NZ_CP050420	Acinetobacter baumannii strain PM193665 plasmid pPM193665_5, complete sequence

747	NZ_CP050422	Acinetobacter baumannii strain VB2200 plasmid pVB2200_1, complete sequence
748	NZ_CP050423	Acinetobacter baumannii strain VB2200 plasmid pVB2200_2, complete sequence
749	NZ_CP050426	Acinetobacter baumannii strain PM194188 plasmid pPM194122_1, complete sequence
750	NZ_CP050427	Acinetobacter baumannii strain PM194188 plasmid pPM194122_2, complete sequence
751	NZ_CP050428	Acinetobacter baumannii strain PM194188 plasmid pPM194122_3, complete sequence
752	NZ_CP050429	Acinetobacter baumannii strain PM194188 plasmid pPM194122_4, complete sequence
753	NZ_CP050430	Acinetobacter baumannii strain PM194188 plasmid pPM194122_5, complete sequence
754	NZ_CP050431	Acinetobacter baumannii strain PM194188 plasmid pPM194122_6, complete sequence
755	NZ_CP050433	Acinetobacter baumannii strain PM194229 plasmid pPM194229_1, complete sequence
756	NZ_CP050434	Acinetobacter baumannii strain PM194229 plasmid pPM194229_2, complete sequence
757	NZ_CP050435	Acinetobacter baumannii strain PM194229 plasmid pPM194229_3, complete sequence
758	NZ_CP050524	Acinetobacter baumannii strain VB7036 plasmid pVB7036_1, complete sequence
759	NZ_CP050525	Acinetobacter baumannii strain VB7036 plasmid pVB7036_2, complete sequence
760	NZ_CP050527	Acinetobacter baumannii strain VB2139 plasmid pVB2139_1, complete sequence
761	NZ_CP050528	Acinetobacter baumannii strain VB2139 plasmid pVB2139_2, complete sequence
762	NZ_CP050529	Acinetobacter baumannii strain VB2139 plasmid pVB2139_3, complete sequence
763	NZ_CP050905	Acinetobacter baumannii strain DT-Ab057 plasmid unnamed1, complete sequence
764	NZ_CP050906	Acinetobacter baumannii strain DT-Ab057 plasmid unnamed2, complete sequence
765	NZ_CP050908	Acinetobacter baumannii strain DT-Ab022 plasmid unnamed1, complete sequence
766	NZ_CP050909	Acinetobacter baumannii strain DT-Ab022 plasmid unnamed2, complete sequence
767	NZ_CP050910	Acinetobacter baumannii strain DT-Ab022 plasmid unnamed3, complete sequence
768	NZ_CP050912	Acinetobacter baumannii strain DT-Ab020 plasmid unnamed1, complete sequence
769	NZ_CP050913	Acinetobacter baumannii strain DT-Ab020 plasmid unnamed2, complete sequence
770	NZ_CP050915	Acinetobacter baumannii strain DT-Ab007 plasmid unnamed1, complete sequence
771	NZ_CP050917	Acinetobacter baumannii strain DT-Ab003 plasmid unnamed1, complete sequence
772	NZ_CP050918	Acinetobacter baumannii strain DT-Ab003 plasmid unnamed2, complete sequence
773	NZ_CP051209	Acinetobacter sp. NEB149 plasmid pMsp179, complete sequence
774	NZ_CP051210	Acinetobacter sp. NEB149 plasmid pMsp65, complete sequence
775	NZ_CP051211	Acinetobacter sp. NEB149 plasmid pMsp22, complete sequence
776	NZ_CP051212	Acinetobacter sp. NEB149 plasmid pMsp11, complete sequence
777	NZ_CP051475	Acinetobacter baumannii strain VB2107 plasmid pVB2107_1, complete sequence
778	NZ_CP051476	Acinetobacter baumannii strain VB2107 plasmid pVB2107_2, complete sequence

779	NZ_CP051477	Acinetobacter baumannii strain VB2107 plasmid pVB2107_3, complete sequence
780	NZ_CP051863	Acinetobacter baumannii strain Ab-C102 plasmid pAb-C102_1, complete sequence
781	NZ_CP051864	Acinetobacter baumannii strain Ab-C102 plasmid pAb-C102_2, complete sequence
782	NZ_CP051865	Acinetobacter baumannii strain Ab-C102 plasmid pAb-C102_3, complete sequence
783	NZ_CP051867	Acinetobacter baumannii strain Ab-C63 plasmid pAb-C63_1, complete sequence
784	NZ_CP051868	Acinetobacter baumannii strain Ab-C63 plasmid pAb-C63_2, complete sequence
785	NZ_CP051870	Acinetobacter baumannii strain Ab-D10a-a plasmid pAb-D10a-a_1, complete sequence
786	NZ_CP051871	Acinetobacter baumannii strain Ab-D10a-a plasmid pAb-D10a-a_2, complete sequence
787	NZ_CP051872	Acinetobacter baumannii strain Ab-D10a-a plasmid pAb-D10a-a_3, complete sequence
788	NZ_CP051873	Acinetobacter baumannii strain Ab-D10a-a plasmid pAb-D10a-a_4, complete sequence
789	NZ_CP051874	Acinetobacter baumannii strain Ab-D10a-a plasmid pAb-D10a-a_5, complete sequence
790	NZ_CP051876	Acinetobacter baumannii strain Ab-B004d-c plasmid pAb-B004d-c_1, complete sequence
791	NZ_CP051877	Acinetobacter baumannii strain Ab-B004d-c plasmid pAb-B004d-c_2, complete sequence
792	NZ_CP051878	Acinetobacter baumannii strain Ab-B004d-c plasmid pAb-B004d-c_3, complete sequence
793	NZ_CP051879	Acinetobacter baumannii strain Ab-B004d-c plasmid pAb-B004d-c_4, complete sequence
794	NZ_CP053099	Acinetobacter baumannii ATCC 17978 plasmid unnamed1, complete sequence
795	NZ_CP053100	Acinetobacter baumannii ATCC 17978 plasmid unnamed2, complete sequence
796	NZ_CP053216	Acinetobacter baumannii strain DT0544C plasmid unnamed1, complete sequence
797	NZ_CP053217	Acinetobacter baumannii strain DT0544C plasmid unnamed2, complete sequence
798	NZ_CP053219	Acinetobacter baumannii strain DT01139C plasmid unnamed1, complete sequence
799	NZ_CP053220	Acinetobacter baumannii strain DT01139C plasmid unnamed2, complete sequence
800	NZ_CP053221	Acinetobacter baumannii strain DT01139C plasmid unnamed3, complete sequence
801	NZ_CP054138	Acinetobacter pittii strain JXA13 plasmid pHNJXA13-1, complete sequence
802	NZ_CP054804	Acinetobacter lwoffii strain FDAARGOS_557 plasmid unnamed3
803	NZ_CP054805	Acinetobacter lwoffii strain FDAARGOS_557 plasmid unnamed2
804	NZ_CP054806	Acinetobacter lwoffii strain FDAARGOS_557 plasmid unnamed1, complete sequence
805	NZ_CP054821	Acinetobacter lwoffii strain FDAARGOS_551 plasmid unnamed1, complete sequence
806	NZ_CP054823	Acinetobacter lwoffii strain FDAARGOS_551 plasmid unnamed3
807	NZ_CP054824	Acinetobacter lwoffii strain FDAARGOS_551 plasmid unnamed2, complete sequence
808	NZ_CP054825	Acinetobacter lwoffii strain FDAARGOS_551 plasmid unnamed4
809	NZ_CP054826	Acinetobacter lwoffii strain FDAARGOS_551 plasmid unnamed5
810	NZ_CP054904	Acinetobacter sp. FDAARGOS_724 plasmid unnamed1, complete sequence

811	NZ_CP054905	Acinetobacter sp. FDAARGOS_724 plasmid unnamed4
812	NZ_CP054906	Acinetobacter sp. FDAARGOS_724 plasmid unnamed3
813	NZ_CP054907	Acinetobacter sp. FDAARGOS_724 plasmid unnamed2, complete sequence
814	NZ_CP055278	Acinetobacter sp. NEB 394 plasmid pBspH10, complete sequence
815	NZ_CP055279	Acinetobacter sp. NEB 394 plasmid pBspH11, complete sequence
816	NZ_CP055280	Acinetobacter sp. NEB 394 plasmid pBspH12, complete sequence
817	NZ_CP055281	Acinetobacter sp. NEB 394 plasmid pBspH13, complete sequence
818	NZ_CP055282	Acinetobacter sp. NEB 394 plasmid pBspH14, complete sequence
819	NZ_CP055283	Acinetobacter sp. NEB 394 plasmid pBspH1, complete sequence
820	NZ_CP055284	Acinetobacter sp. NEB 394 plasmid pBspH2, complete sequence
821	NZ_CP055285	Acinetobacter sp. NEB 394 plasmid pBspH3, complete sequence
822	NZ_CP055286	Acinetobacter sp. NEB 394 plasmid pBspH4, complete sequence
823	NZ_CP055287	Acinetobacter sp. NEB 394 plasmid pBspH5, complete sequence
824	NZ_CP055288	Acinetobacter sp. NEB 394 plasmid pBspH6, complete sequence
825	NZ_CP055289	Acinetobacter sp. NEB 394 plasmid pBspH7, complete sequence
826	NZ_CP055290	Acinetobacter sp. NEB 394 plasmid pBspH8, complete sequence
827	NZ_CP055291	Acinetobacter sp. NEB 394 plasmid pBspH9, complete sequence
828	NZ_CP056785	Acinetobacter baumannii strain TP1 plasmid pTP1A, complete sequence
829	NZ_CP059042	Acinetobacter baumannii strain ATCC 17978 plasmid unnamed, complete sequence
830	NZ_CP059301	Acinetobacter baumannii strain AC1633 plasmid pAC1633-1, complete sequence
831	NZ_CP059302	Acinetobacter baumannii strain AC1633 plasmid pAC1633-4, complete sequence
832	NZ_CP059303	Acinetobacter baumannii strain AC1633 plasmid pAC1633-2, complete sequence
833	NZ_CP059304	Acinetobacter baumannii strain AC1633 plasmid pAC1633-3, complete sequence
834	NZ_CP059387	Acinetobacter baumannii strain 36-1512 plasmid p1.36-1512, complete sequence
835	NZ_CP059388	Acinetobacter baumannii strain 36-1512 plasmid p2.36-1512, complete sequence
836	NZ_CP059389	Acinetobacter baumannii strain 36-1512 plasmid p3.36-1512, complete sequence
837	NZ_CP059390	Acinetobacter baumannii strain 36-1512 plasmid p4.36-1512, complete sequence
838	NZ_CP059478	Acinetobacter baumannii strain 17-84 plasmid p17-84_OXA, complete sequence
839	NZ_CP059559	Acinetobacter junii strain YR7 plasmid pNDM-YR7, complete sequence
840	NZ_CP059685	Acinetobacter radioresistens strain FDAARGOS_731 plasmid unnamed1, complete sequence
841	NZ_CP059686	Acinetobacter radioresistens strain FDAARGOS_731 plasmid unnamed2, complete sequence

842	NZ_CP059687	Acinetobacter radioresistens strain FDAARGOS_731 plasmid unnamed5
843	NZ_CP059688	Acinetobacter radioresistens strain FDAARGOS_731 plasmid unnamed3, complete sequence
844	NZ_CP059689	Acinetobacter radioresistens strain FDAARGOS_731 plasmid unnamed4, complete sequence
845	NZ_CP059730	Acinetobacter baumannii strain AbCTX13 plasmid pAbCTX13_7kb, complete sequence
846	NZ_CP059731	Acinetobacter baumannii strain AbCTX13 plasmid pAbCTX13_17kb, complete sequence
847	NZ_CP060012	Acinetobacter baumannii strain TP2 plasmid pTP2A, complete sequence
848	NZ_CP060014	Acinetobacter baumannii strain TP3 plasmid pTP3A, complete sequence
849	NZ_CP060812	Acinetobacter variabilis strain XM9F202-2 plasmid pXM9F202-2-186k, complete sequence
850	NZ_CP060813	Acinetobacter variabilis strain XM9F202-2 plasmid pXM9F202-2-tetX-90k, complete sequence
851	NZ_CP060814	Acinetobacter variabilis strain XM9F202-2 plasmid pXM9F202-2-17k, complete sequence
852	NZ_CP060815	Acinetobacter variabilis strain XM9F202-2 plasmid pXM9F202-2-13k, complete sequence
853	NZ_CP060816	Acinetobacter variabilis strain XM9F202-2 plasmid pXM9F202-2-2k, complete sequence
854	NZ_CP061515	Acinetobacter baumannii strain CFSAN093710 plasmid pCFSAN093710_1, complete sequence
855	NZ_CP061516	Acinetobacter baumannii strain CFSAN093710 plasmid pCFSAN093710_2, complete sequence
856	NZ_CP061518	Acinetobacter baumannii strain CFSAN093709 plasmid pCFSAN093709, complete sequence
857	NZ_CP061520	Acinetobacter baumannii strain CFSAN093708 plasmid pCFSAN093708, complete sequence
858	NZ_CP061522	Acinetobacter baumannii strain CFSAN093707 plasmid pCFSAN093707, complete sequence
859	NZ_CP061524	Acinetobacter baumannii strain CFSAN093706 plasmid pCFSAN093706, complete sequence
860	NZ_CP061526	Acinetobacter baumannii strain CFSAN093705 plasmid pCFSAN093705, complete sequence
861	NZ_CP061562	Acinetobacter seifertii strain AS73 plasmid pAS73-1, complete sequence
862	NZ_CP061563	Acinetobacter seifertii strain AS73 plasmid pAS73-2, complete sequence
863	NZ_CP061564	Acinetobacter seifertii strain AS73 plasmid pAS73-3, complete sequence
864	NZ_CP061579	Acinetobacter seifertii strain AS63 plasmid pAS63-1, complete sequence
865	NZ_CP061580	Acinetobacter seifertii strain AS63 plasmid pAS63-2, complete sequence
866	NZ_CP061581	Acinetobacter seifertii strain AS63 plasmid pAS63-3, complete sequence
867	NZ_CP061583	Acinetobacter seifertii strain AS62 plasmid pAS62-1, complete sequence
868	NZ_CP061584	Acinetobacter seifertii strain AS62 plasmid pAS62-2, complete sequence
869	NZ_CP061585	Acinetobacter seifertii strain AS62 plasmid pAS62-3, complete sequence
870	NZ_CP061586	Acinetobacter seifertii strain AS62 plasmid pAS62-4, complete sequence

871	NZ_CP061587	Acinetobacter seifertii strain AS62 plasmid pAS62-5, complete sequence
872	NZ_CP061588	Acinetobacter seifertii strain AS62 plasmid pAS62-6, complete sequence
873	NZ_CP061593	Acinetobacter seifertii strain AS60 plasmid pAS60-1, complete sequence
874	NZ_CP061594	Acinetobacter seifertii strain AS60 plasmid pAS60-2, complete sequence
875	NZ_CP061595	Acinetobacter seifertii strain AS60 plasmid pAS60-3, complete sequence
876	NZ_CP061597	Acinetobacter seifertii strain AS59 plasmid pAS59-1, complete sequence
877	NZ_CP061604	Acinetobacter seifertii strain AS53 plasmid pAS53-1, complete sequence
878	NZ_CP061605	Acinetobacter seifertii strain AS53 plasmid pAS53-2, complete sequence
879	NZ_CP061606	Acinetobacter seifertii strain AS53 plasmid pAS53-3, complete sequence
880	NZ_CP061607	Acinetobacter seifertii strain AS53 plasmid pAS53-4, complete sequence
881	NZ_CP061608	Acinetobacter seifertii strain AS53 plasmid pAS53-5, complete sequence
882	NZ_CP061614	Acinetobacter seifertii strain AS51 plasmid pAS51-1, complete sequence
883	NZ_CP061615	Acinetobacter seifertii strain AS51 plasmid pAS51-2, complete sequence
884	NZ_CP061616	Acinetobacter seifertii strain AS51 plasmid pAS51-3, complete sequence
885	NZ_CP061617	Acinetobacter seifertii strain AS51 plasmid pAS51-4, complete sequence
886	NZ_CP061618	Acinetobacter seifertii strain AS51 plasmid pAS51-5, complete sequence
887	NZ_CP061619	Acinetobacter seifertii strain AS51 plasmid pAS51-6, complete sequence
888	NZ_CP061621	Acinetobacter seifertii strain AS49 plasmid pAS49-1, complete sequence
889	NZ_CP061622	Acinetobacter seifertii strain AS49 plasmid pAS49-2, complete sequence
890	NZ_CP061623	Acinetobacter seifertii strain AS49 plasmid pAS49-3, complete sequence
891	NZ_CP061624	Acinetobacter seifertii strain AS49 plasmid pAS49-4, complete sequence
892	NZ_CP061625	Acinetobacter seifertii strain AS49 plasmid pAS49-5, complete sequence
893	NZ_CP061627	Acinetobacter seifertii strain AS48 plasmid pAS48-1, complete sequence
894	NZ_CP061628	Acinetobacter seifertii strain AS48 plasmid pAS48-2, complete sequence
895	NZ_CP061630	Acinetobacter seifertii strain AS47 plasmid pAS47-1, complete sequence
896	NZ_CP061631	Acinetobacter seifertii strain AS47 plasmid pAS47-2, complete sequence
897	NZ_CP061632	Acinetobacter seifertii strain AS47 plasmid pAS47-3, complete sequence
898	NZ_CP061633	Acinetobacter seifertii strain AS47 plasmid pAS47-4, complete sequence
899	NZ_CP061635	Acinetobacter seifertii strain AS41 plasmid pAS41-1, complete sequence
900	NZ_CP061636	Acinetobacter seifertii strain AS41 plasmid pAS41-2, complete sequence
901	NZ_CP061637	Acinetobacter seifertii strain AS41 plasmid pAS41-3, complete sequence
902	NZ_CP061638	Acinetobacter seifertii strain AS41 plasmid pAS41-4, complete sequence

903	NZ_CP061639	Acinetobacter seifertii strain AS41 plasmid pAS41-5, complete sequence
904	NZ_CP061641	Acinetobacter seifertii strain AS40 plasmid pAS40-1, complete sequence
905	NZ_CP061642	Acinetobacter seifertii strain AS40 plasmid pAS40-2, complete sequence
906	NZ_CP061643	Acinetobacter seifertii strain AS40 plasmid pAS40-3, complete sequence
907	NZ_CP061644	Acinetobacter seifertii strain AS40 plasmid pAS40-4, complete sequence
908	NZ_CP061645	Acinetobacter seifertii strain AS40 plasmid pAS40-5, complete sequence
909	NZ_CP061647	Acinetobacter seifertii strain AS39 plasmid pAS39-1, complete sequence
910	NZ_CP061648	Acinetobacter seifertii strain AS39 plasmid pAS39-2, complete sequence
911	NZ_CP061649	Acinetobacter seifertii strain AS39 plasmid pAS39-3, complete sequence
912	NZ_CP061661	Acinetobacter seifertii strain AS31 plasmid pAS31-1, complete sequence
913	NZ_CP061662	Acinetobacter seifertii strain AS31 plasmid pAS31-2, complete sequence
914	NZ_CP061663	Acinetobacter seifertii strain AS31 plasmid pAS31-3, complete sequence
915	NZ_CP061665	Acinetobacter seifertii strain AS28 plasmid pAS28-1, complete sequence
916	NZ_CP061667	Acinetobacter seifertii strain AS25 plasmid pAS25-1, complete sequence
917	NZ_CP061668	Acinetobacter seifertii strain AS25 plasmid pAS25-2, complete sequence
918	NZ_CP061676	Acinetobacter seifertii strain AS17 plasmid pAS17-1, complete sequence
919	NZ_CP061677	Acinetobacter seifertii strain AS17 plasmid pAS17-2, complete sequence
920	NZ_CP061679	Acinetobacter seifertii strain AS11 plasmid pAS11-1, complete sequence
921	NZ_CP061680	Acinetobacter seifertii strain AS11 plasmid pAS11-2, complete sequence
922	NZ_CP061682	Acinetobacter seifertii strain AS9 plasmid pAS9-1, complete sequence
923	NZ_CP061684	Acinetobacter seifertii strain AS5 plasmid pAS5-1, complete sequence
924	NZ_CP061685	Acinetobacter seifertii strain AS5 plasmid pAS5-2, complete sequence
925	NZ_CP061686	Acinetobacter seifertii strain AS5 plasmid pAS5-3, complete sequence
926	NZ_CP061829	Acinetobacter seifertii strain AS42 plasmid pAS42-1, complete sequence
927	NZ_CP061830	Acinetobacter seifertii strain AS42 plasmid pAS42-2, complete sequence
928	NZ_CP061831	Acinetobacter seifertii strain AS42 plasmid pAS42-3, complete sequence
929	NZ_CP062920	Acinetobacter baumannii strain Res13-Abat-PEA21-P4-01-A plasmid unnamed1774, complete sequence
930	NZ_CP062921	Acinetobacter baumannii strain Res13-Abat-PEA21-P4-01-A plasmid unnamed1836, complete sequence
931	NZ_CP062922	Acinetobacter baumannii strain Res13-Abat-PEA21-P4-01-A plasmid unnamednovel_1, complete sequence
932	NZ_CP062923	Acinetobacter baumannii strain Res13-Abat-PEA21-P4-01-A plasmid unnamednovel_2, complete sequence

933	NZ_CP063770	Acinetobacter sp. Ac-14 plasmid unnamed1, complete sequence
934	NZ_CP063771	Acinetobacter sp. Ac-14 plasmid unnamed2, complete sequence
935	NZ_CP065433	Acinetobacter baumannii strain ATCC 17961 plasmid pAB17961-1, complete sequence
936	NZ_CP065434	Acinetobacter baumannii strain ATCC 17961 plasmid pAB17961-2, complete sequence
937	NZ_CP065663	Acinetobacter johnsonii strain FDAARGOS_910 plasmid unnamed1, complete sequence
938	NZ_CP065664	Acinetobacter johnsonii strain FDAARGOS_910 plasmid unnamed2
939	NZ_CP065665	Acinetobacter johnsonii strain FDAARGOS_910 plasmid unnamed3
940	NZ_CP065886	Acinetobacter baumannii strain FDAARGOS_917 plasmid unnamed1, complete sequence
941	NZ_CP065888	Acinetobacter baumannii strain FDAARGOS_917 plasmid unnamed2
942	NZ_CP066017	Acinetobacter baumannii strain FDAARGOS_1036 plasmid unnamed
943	NZ_CP066120	Acinetobacter bereziniae strain GD03185 plasmid unnamed map unlocalized
944	NZ_CP066122	Acinetobacter bereziniae strain GD0320 plasmid unnamed map unlocalized
945	NZ_CP066230	Acinetobacter baumannii strain G20AB011 plasmid pG20AB011-1, complete sequence
946	NZ_CP066231	Acinetobacter baumannii strain G20AB011 plasmid pG20AB011-2, complete sequence
947	NZ_CP066233	Acinetobacter baumannii strain G20AB010 plasmid pG20AB010-1, complete sequence
948	NZ_CP066234	Acinetobacter baumannii strain G20AB010 plasmid pG20AB010-2
949	NZ_CP066236	Acinetobacter baumannii strain G20AB009 plasmid pG20AB009-1, complete sequence
950	NZ_CP066238	Acinetobacter baumannii strain G20AB007 plasmid pG20AB007-1, complete sequence
951	NZ_CP066239	Acinetobacter baumannii strain G20AB007 plasmid pG20AB007-2, complete sequence
952	NZ_CP066240	Acinetobacter baumannii strain G20AB007 plasmid pG20AB007-3, complete sequence
953	NZ_CP067020	Acinetobacter sp. CS-2 plasmid unnamed1, complete sequence
954	NZ_CP067021	Acinetobacter sp. CS-2 plasmid unnamed2, complete sequence
955	NZ_CP067103	Acinetobacter baumannii strain ATCC BAA-1790 plasmid pNC2, complete sequence
956	NZ_CP068175	Acinetobacter ursingii strain FDAARGOS_1096 plasmid unnamed1, complete sequence
957	NZ_CP068177	Acinetobacter ursingii strain FDAARGOS_1096 plasmid unnamed2, complete sequence
958	NZ_CP068178	Acinetobacter ursingii strain FDAARGOS_1096 plasmid unnamed3, complete sequence
959	NZ_CP068179	Acinetobacter ursingii strain FDAARGOS_1096 plasmid unnamed4, complete sequence
960	NZ_CP068181	Acinetobacter ursingii strain FDAARGOS_1095 plasmid unnamed1, complete sequence
961	NZ_CP068182	Acinetobacter ursingii strain FDAARGOS_1095 plasmid unnamed2, complete sequence
962	NZ_CP068183	Acinetobacter ursingii strain FDAARGOS_1095 plasmid unnamed3, complete sequence
963	NZ_CP068184	Acinetobacter ursingii strain FDAARGOS_1095 plasmid unnamed4

964	NZ_CP068185	Acinetobacter johnsonii strain FDAARGOS_1094 plasmid unnamed1, complete sequence
965	NZ_CP068186	Acinetobacter johnsonii strain FDAARGOS_1094 plasmid unnamed2, complete sequence
966	NZ_CP068188	Acinetobacter johnsonii strain FDAARGOS_1094 plasmid unnamed3, complete sequence
967	NZ_CP068189	Acinetobacter johnsonii strain FDAARGOS_1094 plasmid unnamed4, complete sequence
968	NZ_CP068190	Acinetobacter johnsonii strain FDAARGOS_1094 plasmid unnamed5
969	NZ_CP068191	Acinetobacter johnsonii strain FDAARGOS_1094 plasmid unnamed6
970	NZ_CP068192	Acinetobacter johnsonii strain FDAARGOS_1094 plasmid unnamed7
971	NZ_CP068193	Acinetobacter johnsonii strain FDAARGOS_1094 plasmid unnamed8, complete sequence
972	NZ_CP068194	Acinetobacter johnsonii strain FDAARGOS_1094 plasmid unnamed9
973	NZ_CP068196	Acinetobacter johnsonii strain FDAARGOS_1093 plasmid unnamed1, complete sequence
974	NZ_CP068197	Acinetobacter johnsonii strain FDAARGOS_1093 plasmid unnamed2
975	NZ_CP068198	Acinetobacter johnsonii strain FDAARGOS_1093 plasmid unnamed3
976	NZ_CP068199	Acinetobacter johnsonii strain FDAARGOS_1093 plasmid unnamed4
977	NZ_CP068200	Acinetobacter johnsonii strain FDAARGOS_1093 plasmid unnamed5
978	NZ_CP068201	Acinetobacter johnsonii strain FDAARGOS_1093 plasmid unnamed6
979	NZ_CP068202	Acinetobacter johnsonii strain FDAARGOS_1092 plasmid unnamed1, complete sequence
980	NZ_CP068203	Acinetobacter johnsonii strain FDAARGOS_1092 plasmid unnamed2
981	NZ_CP068204	Acinetobacter johnsonii strain FDAARGOS_1092 plasmid unnamed3
982	NZ_CP068205	Acinetobacter johnsonii strain FDAARGOS_1092 plasmid unnamed4, complete sequence
983	NZ_CP068207	Acinetobacter johnsonii strain FDAARGOS_1092 plasmid unnamed5
984	NZ_CP068208	Acinetobacter johnsonii strain FDAARGOS_1092 plasmid unnamed6
985	NZ_CP068209	Acinetobacter johnsonii strain FDAARGOS_1092 plasmid unnamed7, complete sequence
986	NZ_CP068210	Acinetobacter johnsonii strain FDAARGOS_1092 plasmid unnamed8, complete sequence
987	NZ_CP069497	Acinetobacter pittii strain FDAARGOS_1217 plasmid unnamed1, complete sequence
988	NZ_CP069498	Acinetobacter pittii strain FDAARGOS_1217 plasmid unnamed2, complete sequence
989	NZ_CP069505	Acinetobacter pittii strain FDAARGOS_1215 plasmid unnamed1, complete sequence
990	NZ_CP069506	Acinetobacter pittii strain FDAARGOS_1215 plasmid unnamed2, complete sequence

991	NZ_CP069507	Acinetobacter pittii strain FDAARGOS_1215 plasmid unnamed3, complete sequence
992	NZ_CP069508	Acinetobacter pittii strain FDAARGOS_1215 plasmid unnamed4, complete sequence
993	NZ_CP069538	Acinetobacter pittii strain FDAARGOS_1214 plasmid unnamed1, complete sequence
994	NZ_CP069539	Acinetobacter pittii strain FDAARGOS_1214 plasmid unnamed2
995	NZ_CP069540	Acinetobacter pittii strain FDAARGOS_1214 plasmid unnamed3, complete sequence
996	NZ_CP069541	Acinetobacter pittii strain FDAARGOS_1214 plasmid unnamed4
997	NZ_CP069841	Acinetobacter baumannii strain FDAARGOS_1360 plasmid unnamed1, complete sequence
998	NZ_CP069842	Acinetobacter baumannii strain FDAARGOS_1360 plasmid unnamed2, complete sequence
999	NZ_CP069852	Acinetobacter baumannii strain FDAARGOS_1359 plasmid unnamed, complete sequence
1000	NZ_CP070363	Acinetobacter baumannii strain AB5075-VUB plasmid pAB5075-VUB_1, complete sequence
1001	NZ_CP070364	Acinetobacter baumannii strain AB5075-VUB plasmid pAB5075-VUB_2, complete sequence
1002	NZ_CP070365	Acinetobacter baumannii strain AB5075-VUB plasmid pAB5075-VUB_3, complete sequence
1003	NZ_CP070867	Acinetobacter johnsonii strain XY27 plasmid plas1, complete sequence
1004	NZ_CP070868	Acinetobacter johnsonii strain XY27 plasmid plas2, complete sequence
1005	NZ_CP070869	Acinetobacter johnsonii strain XY27 plasmid plas3, complete sequence
1006	NZ_CP071318	Acinetobacter indicus strain GXNN15X4 plasmid pGXNN15, complete sequence
1007	NZ_CP071767	Acinetobacter towneri strain GX3 plasmid pGX3-1, complete sequence
1008	NZ_CP071768	Acinetobacter towneri strain GX3 plasmid pGX3-2, complete sequence
1009	NZ_CP071769	Acinetobacter towneri strain GX5 plasmid pGX5, complete sequence
1010	NZ_CP071772	Acinetobacter towneri strain GX7 plasmid pGX7, complete sequence
1011	NZ_CP071920	Acinetobacter baumannii strain GIMC5510:ABT-897-17 plasmid pABT-897-17, complete sequence
1012	NZ_CP072123	Acinetobacter baumannii strain KSK1 plasmid p1KSK1, complete sequence
1013	NZ_CP072124	Acinetobacter baumannii strain KSK1 plasmid p2KSK1, complete sequence
1014	NZ_CP072125	Acinetobacter baumannii strain KSK1 plasmid p3KSK1, complete sequence
1015	NZ_CP072126	Acinetobacter baumannii strain KSK1 plasmid p4KSK1, complete sequence
1016	NZ_CP072271	Acinetobacter baumannii strain KSK6 plasmid p1KSK6, complete sequence
1017	NZ_CP072272	Acinetobacter baumannii strain KSK6 plasmid p2KSK6, complete sequence
1018	NZ_CP072273	Acinetobacter baumannii strain KSK6 plasmid p3KSK6, complete sequence
1019	NZ_CP072274	Acinetobacter baumannii strain KSK6 plasmid p4KSK6, complete sequence
1020	NZ_CP072276	Acinetobacter baumannii strain KSK7 plasmid p1KSK7, complete sequence

1021	NZ_CP072277	Acinetobacter baumannii strain KSK7 plasmid p2KSK7, complete sequence
1022	NZ_CP072278	Acinetobacter baumannii strain KSK7 plasmid p3KSK7, complete sequence
1023	NZ_CP072279	Acinetobacter baumannii strain KSK7 plasmid p4KSK7, complete sequence
1024	NZ_CP072281	Acinetobacter baumannii strain KSK10 plasmid p1KSK10, complete sequence
1025	NZ_CP072282	Acinetobacter baumannii strain KSK10 plasmid p2KSK10, complete sequence
1026	NZ_CP072283	Acinetobacter baumannii strain KSK10 plasmid p3KSK10, complete sequence
1027	NZ_CP072284	Acinetobacter baumannii strain KSK10 plasmid p4KSK10, complete sequence
1028	NZ_CP072286	Acinetobacter baumannii strain KSK11 plasmid p1KSK11, complete sequence
1029	NZ_CP072287	Acinetobacter baumannii strain KSK11 plasmid p2KSK11, complete sequence
1030	NZ_CP072288	Acinetobacter baumannii strain KSK11 plasmid p3KSK11, complete sequence
1031	NZ_CP072289	Acinetobacter baumannii strain KSK11 plasmid p4KSK11, complete sequence
1032	NZ_CP072291	Acinetobacter baumannii strain KSK18 plasmid p1KSK18, complete sequence
1033	NZ_CP072292	Acinetobacter baumannii strain KSK18 plasmid p2KSK18, complete sequence
1034	NZ_CP072293	Acinetobacter baumannii strain KSK18 plasmid p3KSK18, complete sequence
1035	NZ_CP072294	Acinetobacter baumannii strain KSK18 plasmid p4KSK18, complete sequence
1036	NZ_CP072296	Acinetobacter baumannii strain KSK19 plasmid p1KSK19, complete sequence
1037	NZ_CP072297	Acinetobacter baumannii strain KSK19 plasmid p2KSK19, complete sequence
1038	NZ_CP072298	Acinetobacter baumannii strain KSK19 plasmid p3KSK19, complete sequence
1039	NZ_CP072299	Acinetobacter baumannii strain KSK19 plasmid p4KSK19, complete sequence
1040	NZ_CP072301	Acinetobacter baumannii strain KSK20 plasmid p1KSK20, complete sequence
1041	NZ_CP072302	Acinetobacter baumannii strain KSK20 plasmid p2KSK20, complete sequence
1042	NZ_CP072303	Acinetobacter baumannii strain KSK20 plasmid p3KSK20, complete sequence
1043	NZ_CP072304	Acinetobacter baumannii strain KSK20 plasmid p4KSK20, complete sequence
1044	NZ_CP072306	Acinetobacter baumannii strain KSK Sensitive plasmid p1KSKSensitive, complete sequence
1045	NZ_CP072307	Acinetobacter baumannii strain KSK Sensitive plasmid p2KSKSensitive, complete sequence
1046	NZ_CP072399	Acinetobacter baumannii strain KSK2 plasmid p1KSK2, complete sequence
1047	NZ_CP072400	Acinetobacter baumannii strain KSK2 plasmid p2KSK2, complete sequence
1048	NZ_CP072401	Acinetobacter baumannii strain KSK2 plasmid p3KSK2, complete sequence
1049	NZ_CP072402	Acinetobacter baumannii strain KSK2 plasmid p4KSK2, complete sequence
1050	NZ_CP072527	Acinetobacter baumannii strain DETAB-E227 plasmid pDETAB4, complete sequence
1051	NZ_CP072528	Acinetobacter baumannii strain DETAB-E227 plasmid pDETAB5, complete sequence
1052	NZ_CP072529	Acinetobacter baumannii strain DETAB-E227 plasmid pDETAB6, complete sequence

1053	NZ_CP073061	Acinetobacter baumannii strain DETAB-P39 plasmid pDETAB13, complete sequence
1054	NZ_CP073242	Acinetobacter soli strain M3-1-68 plasmid pM3-1-68, complete sequence
1055	NZ_HG977523	Acinetobacter baumannii strain CS01 plasmid pCS01A, complete sequence
1056	NZ_HG977524	Acinetobacter baumannii strain CS01 plasmid pCS01B, complete sequence
1057	NZ_HG977525	Acinetobacter baumannii strain CS01 plasmid pCS01C, complete sequence
1058	NZ_HG977527	Acinetobacter baumannii strain CR17 plasmid pCR17A
1059	NZ_HG977528	Acinetobacter baumannii strain CR17 plasmid pCR17B
1060	NZ_HG977529	Acinetobacter baumannii strain CR17 plasmid pCR17C
1061	NZ_JQ739157	Acinetobacter pittii strain ABCA95 plasmid pABCA95, complete sequence
1062	NZ_JQ739158	Acinetobacter lwoffii strain ABZ78 plasmid pABZ78, complete sequence
1063	NZ_KF889012	Acinetobacter baumannii TYTH-1 plasmid pAB_CC, complete sequence
1064	NZ_KJ003839	Acinetobacter pittii strain Acinetobacter pitti plasmid pNDM-AP, complete sequence
1065	NZ_KJ534568	Acinetobacter baumannii strain ATCC 223 plasmid AbATCC223, complete sequence
1066	NZ_KM051986	Acinetobacter baumannii strain D72 plasmid pD72-1 clone GC2; global clone 2, complete sequence
1067	NZ_KM210086	Acinetobacter lwoffii strain JN49-1 plasmid pNDM-JN01, complete sequence
1068	NZ_KM210088	Acinetobacter sp. JN247 plasmid pNDM-JN02, complete sequence
1069	NZ_KM922672	Acinetobacter baumannii strain A221 plasmid pAZJ221, complete sequence
1070	NZ_KM923969	Acinetobacter lactucae strain JVAP01 plasmid pNDM-JVAP01, complete sequence
1071	NZ_KM977710	Acinetobacter baumannii strain D46 plasmid pD46-3, complete sequence
1072	NZ_KP890934	Acinetobacter baumannii strain BM2686 plasmid pIP1858, complete sequence
1073	NZ_KR535992	Acinetobacter baumannii strain A105 plasmid pA105-1, complete sequence
1074	NZ_KR535993	Acinetobacter baumannii strain A105 plasmid pA105-2, complete sequence
1075	NZ_KT022421	Acinetobacter baumannii strain ML plasmid pAB-ML, complete sequence
1076	NZ_KT346360	Acinetobacter baumannii strain RCH52 plasmid pRCH52-1, complete sequence
1077	NZ_KT779035	Acinetobacter baumannii strain D4 plasmid pD4, complete sequence
1078	NZ_KT852971	Acinetobacter baumannii strain 255_n plasmid p255n_1 clone MLST16 (Pasteur); Clonal Complex 16; CC16, complete sequence
1079	NZ_KT965092	Acinetobacter towneri strain G165 plasmid pNDM-GJ01, complete sequence
1080	NZ_KT965093	Acinetobacter towneri strain G295 plasmid pNDM-GJ02, complete sequence
1081	NZ_KU500415	Acinetobacter baumannii strain JV1 plasmid ABA02, complete sequence
1082	NZ_KU549175	Acinetobacter baumannii strain C13 plasmid pC13-2 clone GC2, complete sequence
1083	NZ_KU744946	Acinetobacter baumannii strain A297 (RUH875) plasmid pA297-3 clone Global clone 1 (GC1), complete sequence

1084	NZ_KU869528	Acinetobacter baumannii strain A297(RUH875) plasmid pA297-2 clone GC1 (global clone 1), complete sequence
1085	NZ_KU869529	Acinetobacter baumannii strain A297(RUH875) plasmid pA297-1 (pRAY*) clone GC1, complete sequence
1086	NZ_KX118105	Acinetobacter baumannii strain IHIT7853 plasmid IHIT7853-OXA-23, complete sequence
1087	NZ_KX230793	Acinetobacter baumannii strain MAL plasmid pMAL-1, complete sequence
1088	NZ_KX230794	Acinetobacter baumannii strain MAL plasmid pMAL-2, complete sequence
1089	NZ_KX426227	Acinetobacter lwoffii strain ED23-35 plasmid pALWED1.1, complete sequence
1090	NZ_KX426228	Acinetobacter lwoffii strain ED23-35 plasmid pALWED1.3, complete sequence
1091	NZ_KX426229	Acinetobacter lwoffii strain ED45-23 plasmid pALWED2.1, complete sequence
1092	NZ_KX426230	Acinetobacter lwoffii strain ED9-5a plasmid pALWED3.5, complete sequence
1093	NZ_KX426231	Acinetobacter lwoffii strain EK30A plasmid pALWEK1.5, complete sequence
1094	NZ_KX426232	Acinetobacter lwoffii strain VS15 plasmid pALWVS1.1, complete sequence
1095	NZ_KX528687	Acinetobacter lwoffii strain ED9-5a plasmid pALWED3.1, complete sequence
1096	NZ_KX528688	Acinetobacter lwoffii strain EK30A plasmid pALWEK1.1, complete sequence
1097	NZ_KY022424	Acinetobacter baumannii strain Ab8098 plasmid pAb8098, complete sequence
1098	NZ_KY202456	Acinetobacter baumannii strain AB1433 plasmid pIBAC_oxa58_1433, complete sequence
1099	NZ_KY202457	Acinetobacter baumannii strain AB2RED09 plasmid pIBAC_oxa58_2RED, complete sequence
1100	NZ_KY202458	Acinetobacter baumannii strain AB20C15 plasmid pIBAC_oxa58_20C15, complete sequence
1101	NZ_KY216144	Acinetobacter baumannii strain RCH51 plasmid pRCH51-3, complete sequence
1102	NZ_KY499579	Acinetobacter pittii strain CCBH10253 plasmid pAP10253-1, complete sequence
1103	NZ_KY617771	Acinetobacter baumannii strain SGH0823 plasmid pS30-1, complete sequence
1104	NZ_KY704308	Acinetobacter baumannii strain IHIT32296 plasmid pAbIHIT32296, complete sequence
1105	NZ_KY888886	Acinetobacter pittii strain IHIT24944 plasmid pAP24944-OXA-58, complete sequence
1106	NZ_LC483156	Acinetobacter pittii strain SU1805 plasmid pSU1805NDM, complete sequence
1107	NZ_LC537594	Acinetobacter lwoffii strain SU1904 plasmid pSU1904NDM, complete sequence
1108	NZ_LC591943	Acinetobacter variabilis strain RYU24 plasmid pRYU24, complete sequence
1109	NZ_LN833432	Acinetobacter baumannii isolate CHI-32 plasmid pNDM-32, complete sequence
1110	NZ_LN865144	Acinetobacter baumannii isolate CIP70.10 plasmid II, complete sequence
1111	NZ_LN873255	Acinetobacter johnsonii strain LS47-1 plasmid pAJOLS1.1, complete sequence
1112	NZ_LN873256	Acinetobacter lwoffii strain ED23-35 plasmid pALWED1.8, complete sequence
1113	NZ_LN997847	Acinetobacter baumannii isolate R2091 plasmid II, complete sequence

1114	NZ_LR026972	Acinetobacter baumannii strain KCRI-28 isolate RDK36_28 plasmid pKCRI-28-1, complete sequence
1115	NZ_LR026973	Acinetobacter baumannii strain KCRI-43 isolate RDK37_43 plasmid pKCRI-43-1, complete sequence
1116	NZ_LR026974	Acinetobacter baumannii strain KCRI-49 isolate RDK39_49 plasmid pKCRI-49-1, complete sequence
1117	NZ_LT594096	Acinetobacter baumannii strain BAL062 plasmid 2, complete sequence
1118	NZ_LT605060	Acinetobacter baumannii strain NCTC7364 plasmid 2, complete sequence
1119	NZ_LT984690	Acinetobacter baumannii isolate K50 plasmid I, complete sequence
1120	NZ_LT984691	Acinetobacter baumannii isolate K50 plasmid II, complete sequence
1121	NZ_MF078634	Acinetobacter pittii strain HGSA488 plasmid pLS488, complete sequence
1122	NZ_MF078635	Acinetobacter pittii strain HGSA535 plasmid pLS535, complete sequence
1123	NZ_MF399199	Acinetobacter baumannii plasmid pD46-4, complete sequence
1124	NZ_MG100202	Acinetobacter baumannii strain Ab825 plasmid pAb825_36, complete sequence
1125	NZ_MG520098	Acinetobacter baumannii strain Ab244 plasmid pAb244_7, complete sequence
1126	NZ_MG954376	Acinetobacter baumannii strain SGH9601 plasmid pS21-1, complete sequence
1127	NZ_MG954377	Acinetobacter baumannii strain SGH9601 plasmid pS21-2, complete sequence
1128	NZ_MG954378	Acinetobacter baumannii strain SGH0905 plasmid pS32-1, complete sequence
1129	NZ_MG954379	Acinetobacter baumannii strain SGH0905 plasmid pS32-2, complete sequence
1130	NZ_MH220285	Acinetobacter ursingii strain RIVM0002 plasmid pRIVM0002_IMP-4_171109_B03, complete sequence
1131	NZ_MH220286	Acinetobacter ursingii strain RIVM0051 plasmid pRIVM0051_IMP-4, complete sequence
1132	NZ_MH220287	Acinetobacter ursingii strain RIVM0061 plasmid pRIVM0061_IMP-4_171109_B01, complete sequence
1133	NZ_MH362811	Acinetobacter baumannii strain 11A1314CRGN088 plasmid pO237-3, complete sequence
1134	NZ_MH362812	Acinetobacter baumannii strain 11A1314CRGN008 plasmid pO237-1, complete sequence
1135	NZ_MH362813	Acinetobacter baumannii strain 11A1213CRGN055 plasmid pO237-2, complete sequence
1136	NZ_MH999418	Acinetobacter sp. SWBY1 plasmid unnamed, complete sequence
1137	NZ_MK053932	Acinetobacter baumannii strain IEC383 plasmid pIEC383
1138	NZ_MK053934	Acinetobacter nosocomialis strain IEC38057 plasmid pIEC38057, complete sequence
1139	NZ_MK134375	Acinetobacter baumannii strain 34AB plasmid p34AB, complete sequence
1140	NZ_MK243454	Acinetobacter baumannii strain 09A16CRGN0014 plasmid pCRA914-67, complete sequence

1141	NZ_MK323040	Acinetobacter seifertii strain Asp-1069 plasmid pAs1069_a, complete sequence
1142	NZ_MK323041	Acinetobacter seifertii strain Asp-1069 plasmid pAs1069_b, complete sequence
1143	NZ_MK323042	Acinetobacter baumannii strain Acb-45063 plasmid pAb45063_a, complete sequence
1144	NZ_MK323043	Acinetobacter baumannii strain Acb-45063 plasmid pAb45063_b, complete sequence
1145	NZ_MK360916	Acinetobacter baumannii plasmid pAB18PR065-MCR-4.3, complete sequence
1146	NZ_MK386680	Acinetobacter baumannii strain ABAY04001 plasmid pABAY04001_1A, complete sequence
1147	NZ_MK386681	Acinetobacter baumannii strain ABAY09008 plasmid pABAY09008_1B, complete sequence
1148	NZ_MK386682	Acinetobacter baumannii strain ABAY10001 plasmid pABAY10001_1C, complete sequence
1149	NZ_MK386683	Acinetobacter baumannii strain ABAY14012 plasmid pABAY14012_4D, complete sequence
1150	NZ_MK386684	Acinetobacter baumannii strain ABAY15001 plasmid pABAY15001_6E, complete sequence
1151	NZ_MK431775	Acinetobacter baumannii strain 11A14CRGN003 plasmid pO237-4, complete sequence
1152	NZ_MK531536	Acinetobacter baumannii strain MC1 plasmid pMC1.1, complete sequence
1153	NZ_MK531537	Acinetobacter baumannii strain MC1/MC23 plasmid pMC1.2/pMC23.2, complete sequence
1154	NZ_MK531538	Acinetobacter baumannii strain MC23 plasmid pMC23.1, complete sequence
1155	NZ_MK531539	Acinetobacter baumannii strain MC23 plasmid pMC23.3, complete sequence
1156	NZ_MK531540	Acinetobacter baumannii strain MC75 plasmid pMC75.1, complete sequence
1157	NZ_MK531541	Acinetobacter baumannii strain MC75 plasmid pMC75.2, complete sequence
1158	NZ_MK944317	Acinetobacter lwoffii strain M2a plasmid pAVAci144, complete sequence
1159	NZ_MK944318	Acinetobacter lwoffii strain M2a plasmid pAVAci145, complete sequence
1160	NZ_MK944319	Acinetobacter lwoffii strain M2a plasmid pAVAci130, complete sequence
1161	NZ_MK944320	Acinetobacter lwoffii strain M2a plasmid pAVAci84, complete sequence
1162	NZ_MK944321	Acinetobacter lwoffii strain M2a plasmid pAVAci147, complete sequence
1163	NZ_MK944322	Acinetobacter lwoffii strain M2a plasmid pAVAci176, complete sequence
1164	NZ_MK978159	Acinetobacter lwoffii strain M2a plasmid pAVAci117, complete sequence
1165	NZ_MK978160	Acinetobacter lwoffii strain M2a plasmid pAVAci119, complete sequence
1166	NZ_MK978161	Acinetobacter lwoffii strain M2a plasmid pAVAci116, complete sequence
1167	NZ_MK978162	Acinetobacter lwoffii strain M2a plasmid pAVAci14, complete sequence
1168	NZ_MK978163	Acinetobacter lwoffii strain M2a plasmid pAVAci94, complete sequence
1169	NZ_MN266872	Acinetobacter baumannii plasmid pAC1-BRL, complete sequence
1170	NZ_MN461227	Acinetobacter pittii strain A352 plasmid pA352, complete sequence
1171	NZ_MN461228	Acinetobacter pittii strain A2584 plasmid pA2584, complete sequence
1172	NZ_MN481286	Acinetobacter baylyi strain A2702 plasmid pA2702, complete sequence

1173	NZ_MN481287	Acinetobacter pittii strain A2949 plasmid pA2949, complete sequence
1174	NZ_MN495625	Acinetobacter baumannii strain A2485 plasmid pA2485, complete sequence
1175	NZ_MN495626	Acinetobacter baumannii strain A2503 plasmid pA2503, complete sequence
1176	NZ_MT002974	Acinetobacter baumannii strain AB17 plasmid pAB17, complete sequence
1177	NZ_MT107270	Acinetobacter sp. strain SH19PTT10 plasmid pYUSHP10-1, complete sequence
1178	NZ_MT319099	Acinetobacter lwoffii strain VS15 plasmid pALWVS1.4, complete sequence
1179	NZ_MT675918	Acinetobacter lwoffii strain ED9-5A plasmid pALWED3.7, complete sequence
1180	NZ_MT675919	Acinetobacter lwoffii strain EK30A plasmid pALWEK1.12, complete sequence
1181	NZ_MT675920	Acinetobacter lwoffii strain EK30A plasmid pALWEK1.13, complete sequence
1182	NZ_MT675921	Acinetobacter lwoffii strain EK30A plasmid pALWEK1.14, complete sequence
1183	NZ_MT675922	Acinetobacter lwoffii strain EK30A plasmid pALWEK1.16, complete sequence
1184	NZ_MT675923	Acinetobacter lwoffii strain VS15 plasmid pALWVS1.2, complete sequence
1185	NZ_MT675924	Acinetobacter lwoffii strain VS15 plasmid pALWVS1.3, complete sequence
1186	NZ_MT675925	Acinetobacter lwoffii strain EK30A plasmid pALWEK1.15, complete sequence
1187	NZ_MT675926	Acinetobacter lwoffii strain VS15 plasmid pALWVS1.5, complete sequence
1188	NZ_MT742180	Acinetobacter lwoffii strain 12CE1 plasmid pR4WN_12CE1, complete sequence
1189	NZ_MT742181	Acinetobacter johnsonii strain E10B plasmid pR4WN_E10B, complete sequence
1190	NZ_MT742182	Acinetobacter sp. TTH0-4 strain 1BD1 plasmid pR4WN_1BD1, complete sequence
1191	NZ_MT742183	Acinetobacter nosocomialis strain WM98B plasmid pWM98B, complete sequence

Accession number	AMR gene	Identity	Alignment Length/Gene Leng	Coverage	Position in reference	Position in contig	Phenotype	Plasmid
NC_019268	blaNDM-1	100	813/813	100	1..813	9068..9880	Beta-lactam resistance	Acinetobacter lwoffii plasmid pNDM-BJ01, complete sequence
NC_019281	blaNDM-1	100	813/813	100	1..813	9068..9880	Beta-lactam resistance	Acinetobacter lwoffii plasmid pNDM-BJ02, complete sequence
NC_019985	blaNDM-1	100	813/813	100	1..813	9068..9880	Beta-lactam resistance	Acinetobacter baumannii ZW85-1 plasmid pAbNDM-1, complete sequence
NC_020818	blaNDM-1	100	813/813	100	1..813	9068..9880	Beta-lactam resistance	Acinetobacter baumannii plasmid pNDM-AB, complete sequence
NC_023322	blaNDM-1	100	813/813	100	1..813	8810..9622	Beta-lactam resistance	Acinetobacter bereziniae plasmid pNDM-40-1, complete sequence
NC_024959	blaNDM-1	100	813/813	100	1..813	8615..9427	Beta-lactam resistance	Acinetobacter calcoaceticus plasmid pNDM-WS2, complete sequence
NC_024999	blaNDM-1	100	813/813	100	1..813	8615..9427	Beta-lactam resistance	Acinetobacter junii plasmid pNDM-WS1, complete sequence
NC_025000	blaNDM-1	100	813/813	100	1..813	9068..9880	Beta-lactam resistance	Acinetobacter lwoffii plasmid pNDM-Iz4b, complete sequence
NC_025116	blaNDM-1	100	813/813	100	1..813	17669..18481	Beta-lactam resistance	Acinetobacter sp. M131 plasmid pM131_NDM1, complete sequence
NZ_AP023079	blaNDM-1	100	813/813	100	1..813	17685..18497	Beta-lactam resistance	Acinetobacter baumannii strain OCU_Ac16a plasmid pOCU_Ac16a_2, complete sequence
NZ_CP010370	blaNDM-1	100	813/813	100	1..813	16430..17242	Beta-lactam resistance	Acinetobacter nosocomialis strain 6411 plasmid p6411-9.012kb, complete sequence
NZ_CP010399	blaNDM-1	100	766/813	94.21894	1..766	46509..47274	Beta-lactam resistance	Acinetobacter baumannii strain 6200 plasmid p6200-47.274kb, complete sequence
NZ_CP010399	blaNDM-16	100	766/813	94.21894	1..766	46509..47274	Beta-lactam resistance	Acinetobacter baumannii strain 6200 plasmid p6200-47.274kb, complete sequence
NZ_CP014478	blaNDM-1	100	813/813	100	1..813	69791..70603	Beta-lactam resistance	Acinetobacter pittii strain AP_882 plasmid pNDM-AP_882, complete sequence
NZ_CP026127	blaNDM-1	100	813/813	100	1..813	12011..12823	Beta-lactam resistance	Acinetobacter baumannii strain ABNIH28 plasmid pNDM-0285, complete sequence
NZ_CP026425	blaNDM-1	100	813/813	100	1..813	23465..24277	Beta-lactam resistance	Acinetobacter sp. ACNIH1 plasmid pNDM-9c17, complete sequence
NZ_CP026617	blaNDM-1	100	813/813	100	1..813	9753..10565	Beta-lactam resistance	Acinetobacter sp. SWBY1 plasmid pSWBY1, complete sequence
NZ_CP027532	blaNDM-1	100	813/813	100	1..813	27410..28222	Beta-lactam resistance	Acinetobacter baumannii strain AR_0088 plasmid unnamed2, complete sequence
NZ_CP028560	blaNDM-1	100	813/813	100	1..813	57370..58182	Beta-lactam resistance	Acinetobacter sp. WCHA45 plasmid pNDM1_010045, complete sequence
NZ_CP032132	blaNDM-1	100	813/813	100	1..813	20893..21705	Beta-lactam resistance	Acinetobacter chinensis strain WCHAc010005 plasmid pNDM1_010005, complete sequence
NZ_CP032142	blaNDM-1	100	813/813	100	1..813	20196..21008	Beta-lactam resistance	Acinetobacter sp. WCHAc010052 plasmid pNDM1_010052, complete sequence
NZ_CP032278	blaNDM-1	100	813/813	100	1..813	27148..27960	Beta-lactam resistance	Acinetobacter sp. WCHAc010034 plasmid pNDM1_010034, complete sequence
NZ_CP032284	blaNDM-1	100	813/813	100	1..813	54853..55665	Beta-lactam resistance	Acinetobacter sp. WCHA55 plasmid pNDM1_010055, complete sequence
NZ_CP035935	blaNDM-1	100	813/813	100	1..813	33751..34563	Beta-lactam resistance	Acinetobacter cumulans strain WCHAc060092 plasmid pNDM1_060092, complete sequence
NZ_CP041229	blaNDM-1	100	813/813	100	1..813	43595..44407	Beta-lactam resistance	Acinetobacter haemolyticus strain AN54 plasmid pAhaeAN54e, complete sequence
NZ_CP043053	blaNDM-1	100	813/813	100	1..813	84638..85450	Beta-lactam resistance	Acinetobacter pittii strain AP43 plasmid pAP43-OXA58-NDM1, complete sequence
NZ_CP043309	blaNDM-1	100	813/813	100	1..813	250228..251040	Beta-lactam resistance	Acinetobacter johnsonii strain Acsw19 plasmid pAcsw19-2, complete sequence
NZ_CP044446	blaNDM-1	100	813/813	100	1..813	78851..79663	Beta-lactam resistance	Acinetobacter indicus strain CMG3-2 plasmid pCMG3-2-1, complete sequence
NZ_CP044451	blaNDM-1	100	813/813	100	1..813	86444..87256	Beta-lactam resistance	Acinetobacter indicus strain MMS9-2 plasmid pMMS9-2-1, complete sequence
NZ_CP044464	blaNDM-1	100	813/813	100	1..813	98889..99701	Beta-lactam resistance	Acinetobacter schindleri strain HZE23-1 plasmid pHZE23-1-1, complete sequence
NZ_CP044475	blaNDM-1	100	813/813	100	1..813	13913..14725	Beta-lactam resistance	Acinetobacter schindleri strain HZE33-1 plasmid pHZE33-1-1, complete sequence
NZ_CP044484	blaNDM-1	100	813/813	100	1..813	23995..24807	Beta-lactam resistance	Acinetobacter schindleri strain HZE30-1 plasmid pHZE30-1-1, complete sequence
NZ_CP045128	blaNDM-1	100	813/813	100	1..813	22880..23692	Beta-lactam resistance	Acinetobacter indicus strain XG03 plasmid pXG03-X3, complete sequence
NZ_CP045130	blaNDM-1	100	813/813	100	1..813	32161..32973	Beta-lactam resistance	Acinetobacter indicus strain TQ04 plasmid p4TQ-NDM, complete sequence
NZ_CP045133	blaNDM-1	100	813/813	100	1..813	7221..8033	Beta-lactam resistance	Acinetobacter indicus strain TQ18 plasmid p18TQ-NDM, complete sequence
NZ_CP045136	blaNDM-1	100	813/813	100	1..813	22880..23692	Beta-lactam resistance	Acinetobacter indicus strain XG01 plasmid pXG01-X3, complete sequence
NZ_CP045197	blaNDM-1	100	813/813	100	1..813	39140..39952	Beta-lactam resistance	Acinetobacter indicus strain TQ23 plasmid p23TQ-NDM, complete sequence
NZ_CP045561	blaNDM-1	100	813/813	100	1..813	70735..71547	Beta-lactam resistance	Acinetobacter nosocomialis strain AC1530 plasmid pAC1530, complete sequence
NZ_CP046043	blaNDM-1	100	813/813	100	1..813	30617..31429	Beta-lactam resistance	Acinetobacter towneri strain 19110F47 plasmid p19110F47-1, complete sequence
NZ_CP047975	blaNDM-1	100	813/813	100	1..813	61381..62193	Beta-lactam resistance	Acinetobacter baumannii strain DETAB-P2 plasmid pDETAB2, complete sequence
NZ_CP048828	blaNDM-1	100	813/813	100	1..813	201603..202415	Beta-lactam resistance	Acinetobacter baumannii strain ABF9692 plasmid pABF9692, complete sequence
NZ_CP050416	blaNDM-1	100	813/813	100	1..813	57875..58687	Beta-lactam resistance	Acinetobacter baumannii strain PM193665 plasmid pPM193665_1, complete sequence
NZ_CP050426	blaNDM-1	100	813/813	100	1..813	57875..58687	Beta-lactam resistance	Acinetobacter baumannii strain PM194188 plasmid pPM194122_1, complete sequence
NZ_CP053220	blaNDM-1	100	813/813	100	1..813	58992..59804	Beta-lactam resistance	Acinetobacter baumannii strain DT01139C plasmid unnamed2, complete sequence
NZ_CP054138	blaNDM-1	100	813/813	100	1..813	101397..102209	Beta-lactam resistance	Acinetobacter pittii strain JXA13 plasmid pHNJXA13-1, complete sequence
NZ_CP059301	blaNDM-1	100	813/813	100	1..813	73124..73936	Beta-lactam resistance	Acinetobacter baumannii strain AC1633 plasmid pAC1633-1, complete sequence
NZ_CP059559	blaNDM-1	99.88	813/813	100	1..813	36031..36843	Beta-lactam resistance	Acinetobacter junii strain YR7 plasmid pNDM-YR7, complete sequence
NZ_CP067021	blaNDM-1	100	813/813	100	1..813	17039..17851	Beta-lactam resistance	Acinetobacter sp. CS-2 plasmid unnamed2, complete sequence
NZ_CP072528	blaNDM-1	100	813/813	100	1..813	79595..80407	Beta-lactam resistance	Acinetobacter baumannii strain DETAB-E227 plasmid pDETAB5, complete sequence
NZ_JQ739157	blaNDM-1	100	813/813	100	1..813	2312..3124	Beta-lactam resistance	Acinetobacter pittii strain ABCA95 plasmid pABCA95, complete sequence
NZ_KJ003839	blaNDM-1	100	813/813	100	1..813	25918..26730	Beta-lactam resistance	Acinetobacter pittii strain Acinetobacter pitti plasmid pNDM-AP, complete sequence
NZ_KM210086	blaNDM-14	100	813/813	100	1..813	9068..9880	Beta-lactam resistance	Acinetobacter lwoffii strain JN49-1 plasmid pNDM-JN01, complete sequence
NZ_KM210088	blaNDM-1	100	813/813	100	1..813	9068..9880	Beta-lactam resistance	Acinetobacter sp. JN247 plasmid pNDM-JN02, complete sequence
NZ_KM923969	blaNDM-1	100	813/813	100	1..813	9068..9880	Beta-lactam resistance	Acinetobacter lactucae strain JVAP01 plasmid pNDM-JVAP01, complete sequence
NZ_KT965092	blaNDM-1	100	813/813	100	1..813	14253..15065	Beta-lactam resistance	Acinetobacter towneri strain G165 plasmid pNDM-GJ01, complete sequence
NZ_KT965093	blaNDM-1	100	813/813	100	1..813	5229..6041	Beta-lactam resistance	Acinetobacter towneri strain G295 plasmid pNDM-GJ02, complete sequence
NZ_LC483156	blaNDM-1	100	813/813	100	1..813	31134..31946	Beta-lactam resistance	Acinetobacter pittii strain SU1805 plasmid pSU1805NDM, complete sequence

NZ_LC537594	blaNDM-1	100	813/813	100	1..813	33772..34584	Beta-lactam resistance	Acinetobacter lwoffii strain SU1904 plasmid pSU1904NDM, complete sequence
NZ_LC591943	blaNDM-1	100	813/813	100	1..813	64552..65364	Beta-lactam resistance	Acinetobacter variabilis strain RYU24 plasmid pRYU24, complete sequence
NZ_LN833432	blaNDM-1	100	813/813	100	1..813	46686..47498	Beta-lactam resistance	Acinetobacter baumannii isolate CHI-32 plasmid pNDM-32, complete sequence
NZ_MK053932	blaNDM-1	100	813/813	100	1..813	9068..9880	Beta-lactam resistance	Acinetobacter baumannii strain IEC383 plasmid pIEC383
NZ_MK053934	blaNDM-1	99.88	813/813	99.877	1..813	31208..32019	Beta-lactam resistance	Acinetobacter nosocomialis strain IEC38057 plasmid pIEC38057, complete sequence
NZ_MT002974	blaNDM-1	100	813/813	100	1..813	31208..32020	Beta-lactam resistance	Acinetobacter baumannii strain AB17 plasmid pAB17, complete sequence

Accession	Description	Max Score	Total Score	Query Cover	E value	Per. ident	Acc. Len
CP059559.1	Acinetobacter junii strain YR7 plasmid pNDM-YR7, complete sequence	8268	8507	100%	0	100	45911
MT002974.1	Acinetobacter baumannii strain AB17 plasmid pAB17, complete sequence	8263	8501	100%	0	99.98	41087
CP044446.1	Acinetobacter indicus strain CMG3-2 plasmid pCMG3-2-1, complete sequence	8263	12714	100%	0	99.98	120957
CP044475.1	Acinetobacter schindleri strain HZE33-1 plasmid pHZE33-1-1, complete sequence	8263	12714	100%	0	99.98	132305
LR697132.1	Klebsiella pneumoniae isolate 836ac8ea-b38d-11e9-8998-68b599768938 genome assembly, plasmid: p14ARS_VSM0843-1	8263	8501	100%	0	99.98	95284
LR697099.1	Klebsiella pneumoniae isolate 8329a5f4-b38d-11e9-8998-68b599768938 genome assembly, plasmid: p13ARS_GMH0099	8263	8501	100%	0	99.98	273158
CP020524.1	Escherichia coli strain 190 plasmid unnamed1, complete sequence	8263	8501	100%	0	99.98	177196
MH445382.1	Acinetobacter baylyi strain NB01 plasmid pNDM-NB01, partial sequence	8263	8501	100%	0	99.98	32131
MG462728.1	Escherichia coli strain AMA1416 plasmid pAMA1416, complete sequence	8263	8501	100%	0	99.98	190059
CP027532.1	Acinetobacter baumannii strain AR_0088 plasmid unnamed2, complete sequence	8263	8501	100%	0	99.98	41087
CP026425.1	Acinetobacter sp. ACNIH1 plasmid pNDM-9c17, complete sequence	8263	8501	100%	0	99.98	39361
CP026127.1	Acinetobacter baumannii strain ABNIH28 plasmid pNDM-0285, complete sequence	8263	8501	100%	0	99.98	39359
KX832928.1	Providencia rettgeri strain 06-1619 plasmid p06-1619-NDM, complete sequence	8263	8501	100%	0	99.98	54712
CP021961.1	Klebsiella pneumoniae strain AR_0139 plasmid tig00000006, complete sequence	8263	8501	100%	0	99.98	97389
AP018143.1	Escherichia coli plasmid pM214_AC2 DNA, complete genome, isolate: M214	8263	10487	100%	0	99.98	176026
CP021210.1	Escherichia coli strain strain Z247 plasmid p2474-NDM1, complete sequence	8263	8501	100%	0	99.98	75553
KP770032.1	Citrobacter freundii strain Pn5 genomic sequence	8263	8501	100%	0	99.98	12951
KP347609.1	Acinetobacter soli strain Cu244 blaNDM-1 gene cluster, partial sequence	8263	8501	100%	0	99.98	7937
KM210088.1	Acinetobacter sp. JN247 plasmid pNDM-JN02, complete sequence	8263	8501	100%	0	99.98	41084
CP010370.2	Acinetobacter nosocomialis strain 6411 plasmid p6411-9.012kb, complete sequence	8263	10498	100%	0	99.98	47274
KP009590.1	Acinetobacter baumannii strain GB661 transposases, AphA6 (aphA6), beta-lactamase NDM-1 (blaNDM-1), Ble (ble), phosphoribosylanthranilate isomerase (trpF), and twin-arginine translocation pathway signal sequence domain protein (dsbC) genes, complete cds	8263	8501	100%	0	99.98	7622
CP010391.1	Klebsiella pneumoniae strain 6234 plasmid p6234-178.193kb, complete sequence	8263	10493	100%	0	99.98	178193
OK500124.1	Providencia rettgeri strain PROV275 plasmid pPROV275-2NDM, complete sequence	8263	8501	100%	0	99.98	273271
CP098041.1	Providencia rettgeri strain 18004577 plasmid p18001477_NDM, complete sequence	8263	8501	100%	0	99.98	273271
CP095676.1	Providencia stuartii strain dmpro_s193a plasmid p_dmpro_s193a_NDM1, complete sequence	8263	8501	100%	0	99.98	287607
CP095673.1	Enterobacter hormaechei strain dmeb_c388 plasmid p_dmeb_c388_NDM1, complete sequence	8263	8501	100%	0	99.98	158245
CP095599.1	Klebsiella pneumoniae strain dm186b plasmid p_dm186b_NDM1, complete sequence	8263	10487	100%	0	99.98	174692
CP095597.1	Klebsiella pneumoniae strain dm12b plasmid p_dm12b_NDM1, complete sequence	8263	10482	100%	0	99.98	173482
CP095593.1	Providencia stuartii strain bpro_s46a plasmid p_bpro_s46a_NDM1, complete sequence	8263	8501	100%	0	99.98	296291
CP095592.1	Providencia stuartii strain bpro_s12 plasmid p_bpro_s12_NDM1, complete sequence	8263	8501	100%	0	99.98	296291
CP095591.1	Providencia stuartii strain bpro_s102a plasmid p_bpro_s102a_NDM1, complete sequence	8263	8501	100%	0	99.98	296291
CP095677.1	Providencia stuartii strain p_dmpro_s749a_NDM1	8263	8501	100%	0	99.98	111974
MZ342958.1	Citrobacter werkmanii strain LYYSPS2 plasmid pLYYSPS2-3, complete sequence	8263	8501	100%	0	99.98	87232
CP091361.1	Acinetobacter baumannii strain AB177-VUB chromosome, complete genome	8263	32703	100%	0	99.98	4014141
CP090384.1	Acinetobacter towneri strain SCLZS30 plasmid pNDM_SCLZS30, complete sequence	8263	8501	100%	0	99.98	47845
CP089137.1	Escherichia coli strain XD35 plasmid pXD35004, complete sequence	8263	8501	100%	0	99.98	85891
JX072963.1	Acinetobacter sp. M131 plasmid pM131_NDM1, complete sequence	8263	8501	100%	0	99.98	47271
KF295828.1	Providencia rettgeri strain 09ACRGNY2001 plasmid pPrY2001, complete sequence	8263	8501	100%	0	99.98	113295
JN377410.2	Acinetobacter baumannii ZW85-1 plasmid pAbNDM-1, complete sequence	8263	12461	100%	0	99.98	48368
MW073138.1	Acinetobacter sp. strain FL51 plasmid pNDM-FL51, complete sequence	8263	8501	100%	0	99.98	41068
KC503911.1	Acinetobacter baumannii strain GF216 plasmid pNDM-AB, complete sequence	8263	10414	100%	0	99.98	47098
JX441323.1	Acinetobacter johnsonii plasmid pNDM-XC1, partial sequence	8263	8501	100%	0	99.98	12376
CP060013.1	Acinetobacter baumannii strain TP3 chromosome, complete genome	8263	19058	100%	0	99.98	3871732
CP060011.1	Acinetobacter baumannii strain TP2 chromosome, complete genome	8263	18999	100%	0	99.98	3870560
JN616388.1	Acinetobacter lwoffii strain SGC-HZ9 plasmid pAL-1, partial sequence	8263	8501	100%	0	99.98	6451
JQ060896.1	Acinetobacter lwoffii strain WJ10659 plasmid pNDM-BJ02, complete sequence	8263	8501	100%	0	99.98	46165
JQ001791.1	Acinetobacter lwoffii strain WJ10621 plasmid pNDM-BJ01, complete sequence	8263	10476	100%	0	99.98	47274
MT897966.1	Citrobacter freundii plasmid pCf638, partial sequence	8263	8501	100%	0	99.98	16444
LR697127.1	Escherichia coli isolate 83804cd8-b38d-11e9-8998-68b599768938 genome assembly, plasmid: p14ARS_NMC0074-2	8257	10487	100%	0	99.96	146817
CP035935.1	Acinetobacter cumulans strain WCHAc060092 plasmid pNDM1_060092, complete sequence	8257	12688	100%	0	99.96	48560
CP032142.1	Acinetobacter sp. WCHAc010052 plasmid pNDM1_010052, complete sequence	8257	8630	100%	0	99.96	39365
KM210086.1	Acinetobacter lwoffii strain JN49-1 plasmid pNDM-JN01, complete sequence	8257	8496	100%	0	99.96	41084
KJ003839.1	Acinetobacter pittii strain Acinetobacter pitti plasmid pNDM-AP, complete sequence	8257	8630	100%	0	99.96	39364

KJ547696.1	Acinetobacter lwoffii strain lz4b plasmid pNDM-lz4b, complete sequence	8257	10482	100%	0	99.96	46570
CP079750.1	Acinetobacter johnsonii strain MR1 plasmid pMRB, complete sequence	8257	8496	100%	0	99.96	41087
MK053934.1	Acinetobacter nosocomialis strain IEC38057 plasmid pIEC38057, complete sequence	8255	8494	100%	0	99.96	41085
CP021962.1	Klebsiella pneumoniae strain AR_0139 plasmid tig00000008, complete sequence	8255	8494	100%	0	99.96	132217
CP044451.1	Acinetobacter indicus strain MMS9-2 plasmid pMMS9-2-1, complete sequence	8251	12703	100%	0	99.93	121603
CP045136.1	Acinetobacter indicus strain XG01 plasmid pXG01-X3, complete sequence	8251	8490	100%	0	99.93	103629
CP053220.1	Acinetobacter baumannii strain DT01139C plasmid unnamed2, complete sequence	8251	12885	100%	0	99.93	63650
AP023079.1	Acinetobacter baumannii OCU_Ac16a plasmid pOCU_Ac16a_2 DNA, complete sequence	8251	8490	100%	0	99.93	41087
CP095609.1	Klebsiella pneumoniae strain dm330 plasmid p_dm330_NDM1, complete sequence	8250	10474	100%	0	99.93	154399
CP032278.1	Acinetobacter sp. WCHAc010034 plasmid pNDM1_010034, complete sequence	8246	14635	100%	0	99.91	49649
KM923969.1	Acinetobacter dijkshoorniae strain JVAP01 plasmid pNDM-JVAP01, complete sequence	8246	10471	100%	0	99.91	47268
CP078046.1	Acinetobacter lwoffii strain AL_065 plasmid pAL_065-2, complete sequence	8246	16101	100%	0	99.91	284005
CP095567.1	Klebsiella pneumoniae strain b193b plasmid p_b193b_NDM1, complete sequence	8242	10458	100%	0	99.91	173564
CP032132.1	Acinetobacter chinensis strain WCHAc010005 plasmid pNDM1_010005, complete sequence	8240	10449	100%	0	99.89	39357
MK053932.1	Acinetobacter baumannii strain IEC383 plasmid pIEC383	8235	10449	100%	0	99.87	47283
CP078043.1	Acinetobacter junii strain AJ_068 plasmid pAJ_068-2, complete sequence	8235	22929	100%	0	99.87	264416
CP038644.1	Acinetobacter baumannii strain ACN21 chromosome, complete genome	8233	21576	100%	0	99.84	3827138
CP095620.1	Klebsiella pneumoniae strain dm478b plasmid p_dm478b_NDM1, complete sequence	8233	10009	100%	0	99.87	174558
CP065392.1	Acinetobacter baumannii strain AbBAS-1 chromosome	8227	33804	100%	0	99.84	4007838
KX503323.1	Escherichia coli strain HNEC46 plasmid PHNEC46, complete sequence	8218	8457	100%	0	99.82	74046
KF856624.1	Proteus mirabilis strain PEL ThdF (thdF) gene, partial cds, Salmonella genomic island 1, complete sequence, intI1 (intI1), AacA4 (aacA4), AadB (aadb), DhfrA1 (dhfrA1), OrfC (orfC), QacEdelta1 (qacEdelta1), and SulI (sulI) genes, complete cds; tetA pseudogene, complete sequence; VEB-6 (blaVEB-6), QacEdelta1 (qacEdelta1), SulI (sulI), and ISCR1 (orf513) genes, complete cds; orfX pseudogene, complete sequence; OrfY (orfY) and ISAba14, TnpA (tnpA) genes, complete cds; tnpB pseudogene, complete sequence; AphA6 (aphA6), transposase (tnpA), NDM-1 (blaNDM-1), bleMBL (bleMBL), TrpF (trpF), DHA-1 (blaDHA-1), AmpR (ampR), QacEdelta1 (qacEdelta1), SulI (sulI), ISCR1 TnpA (tnpA), AmpR (ampR), HybF (hybF), QacEdelta1 (qacEdelta1), and SulI (sul1) genes, complete cds; acetyltransferase domain protein pseudogene, complete sequence; and TnpA transposition transposase (tnpA), TnpR (tnpR), MerR (merR), MerF (merF), MerP (merP), MerT (merT), MerA (merA), MerD (merD), MerE (merE), TniR (tnpR), TniQ (tniQ), TniB (tniB), TniA (tniA), hypothetical protein, HipB (hipB), HipA (hipA), and hypothetical protein genes, complete cds	8218	8457	100%	0	99.8	67496
LC483156.1	Acinetobacter pittii SU1805 plasmid pSU1805NDM DNA, complete sequence	8202	8440	100%	0	99.69	41022
LN611576.1	Acinetobacter haemolyticus plasmid pNDM-69122 partial sequence, strain 69122-EW	8196	8435	99%	0	99.95	8519
CP045133.1	Acinetobacter indicus strain TQ18 plasmid p18TQ-NDM, complete sequence	8183	8401	100%	0	99.66	40439
CP045130.1	Acinetobacter indicus strain TQ04 plasmid p4TQ-NDM, complete sequence	8183	8414	100%	0	99.66	41086
CP067021.1	Acinetobacter sp. CS-2 plasmid unnamed2, complete sequence	8165	21074	100%	0	99.98	283930
CP078027.1	Acinetobacter variabilis strain AV_175 chromosome, complete genome	8080	34022	100%	0	99.24	3252197
KJ018153.1	Acinetobacter calcoaceticus strain NDM-WS2 plasmid pNDM-WS2 sequence	8072	8311	97%	0	99.93	13940
KF702385.1	Acinetobacter bereziniae strain CHI-40-1 plasmid pNDM-40-1, complete sequence	8037	10262	97%	0	99.98	45826
LC537594.1	Acinetobacter lwoffii SU1904 plasmid pSU1904NDM DNA, complete sequence	7952	8191	100%	0	98.62	43651
CP045540.1	Proteus mirabilis strain CRE14IB plasmid pIB_NDM_1, complete sequence	7749	7988	93%	0	99.98	99278
JN687470.1	Providencia stuartii plasmid pMR0211, complete sequence	7749	7988	93%	0	99.98	178277
CP010399.1	Acinetobacter baumannii strain 6200 plasmid p6200-47.274kb, complete sequence	7494	10494	100%	0	99.98	47274
JF714412.2	Escherichia coli strain N10-2337 plasmid pNDM102337, complete sequence	7280	7885	88%	0	99.97	165974
LR822055.1	Citrobacter werkmanii isolate BB1472 genome assembly, plasmid: pCW-NDM-1_	7107	7345	86%	0	99.97	161908
LR822058.1	Escherichia coli isolate BB1471 genome assembly, plasmid: plasmoidNDM-1_	7105	7343	85%	0	99.97	9301
CP077964.1	Proteus mirabilis strain 6Pmi283 plasmid p6Pmi283-NDM, complete sequence	7020	7258	85%	0	99.95	211006
CP030876.1	Klebsiella pneumoniae strain JNM10C3 plasmid pKJNM10C3.1	6841	7079	83%	0	99.81	190163
MZ836799.1	Escherichia coli strain EC-13-49 plasmid pEC-13-49-NDM-1, complete sequence	6820	7059	82%	0	99.97	214323
MZ836796.1	Escherichia coli strain EC-13-22 plasmid pEC-13-22-NDM-1, complete sequence	6820	7059	82%	0	99.97	212551
KU764665.1	Enterobacter aerogenes strain HN0711 plasmid pHN-NDM0711, partial sequence	6815	7053	82%	0	99.95	18622
KP282691.1	Acinetobacter bereziniae strain ABCA242 plasmid pABCA242 aphA6 (aphA6), transposase (ABCA242_0002), NDM-1 (blaNDM-1), bleomycin resistance protein (ABCA242_0004), TrpF (trpF), DsbC (dsbC), CutA1 (cuA1), GroES (groES), and GroEL (groEL) genes, complete cds; ABAC242_0011 gene, complete sequence; and Resolvase (ABCA242_0012) gene, complete cds	6445	6684	78%	0	99.97	12866
JQ739157.2	Acinetobacter pittii strain ABCA95 plasmid pABCA95, complete sequence	6445	6684	78%	0	99.97	6544
KP282692.1	Acinetobacter baumannii strain ABC3229 plasmid pABC3229 AphA6 (aphA6), hypothetical protein (ABC3229_0002), BlaNDM-1 (blaNDM-1), bleomycin resistance protein (ABC3229_0004), TrpF (trpF), DsbC (dsbC), CutA1 (cutA1), GroES (groES), GroEL (groEL), and resolvase (ABC3229_0010) genes, complete cds	6440	6678	78%	0	99.94	11777

LR822056.1	Providencia rettgeri isolate BB1487 genome assembly, plasmid: pPR-NDM-1B_	6423	6662	77%	0	99.97	210081
LR822049.1	Providencia rettgeri isolate BB1467 genome assembly, plasmid: pPR-NDM-1A_	6423	6662	77%	0	99.97	110068
JQ080305.2	Acinetobacter haemolyticus plasmid pABC7926, partial sequence	6423	8648	78%	0	99.86	12863
